# Gut microbiota depletion and FXR inhibition exacerbates zonal hepatotoxicity of sunitinib

**DOI:** 10.7150/thno.99926

**Published:** 2024-10-28

**Authors:** Qi Zhao, Yingmei Lu, Jingyi Duan, Dan Du, Qianlun Pu, Fei Li

**Affiliations:** 1 Department of Gastroenterology & Hepatology, Laboratory of Hepatointestinal Diseases and Metabolism, Frontiers Science Center for Disease-related Molecular Network, West China Hospital, Sichuan University, Chengdu, 610041, China.; 2 Huaxi Joint Centre for Gastrointestinal Cancer, West China Hospital, Sichuan University, Chengdu, 610041, China.; 3 Advanced Mass Spectrometry Center, Research Core Facility, Frontiers Science Center for Disease-related Molecular Network, West China Hospital, Sichuan University, Chengdu, 610041, China.; 4 Huaxi Joint Centre for Gastrointestinal Cancer, State Key Laboratory of Respiratory Health and Multimorbidity, West China Hospital, Sichuan University, Chengdu, 610041, China.

**Keywords:** FXR, gut microbiota, spatial metabolomics, sunitinib, zonal hepatotoxicity.

## Abstract

**Rationale:** Sunitinib is a small-molecule tyrosine kinase inhibitor associated with the side-effect of liver injury. The impaired cell type in liver and the hepatotoxicity mechanisms is still unclear.

**Methods:** Spatial metabolomics, transmission electron microscopy, immunofluorescence co-staining, and isolation of bile duct cells and liver sinusoidal endothelial cells (LSECs) were used to evaluate the zonated hepatotoxicity of sunitinib. Farnesoid X receptor (FXR) conditional knockout mice, metagenomics analysis, bacteria clearance, bacterial culture, *Parabacteroides distasonis* and 3-oxolithocholic acid supplementation were used to evaluate the hepatotoxicity mechanisms of sunitinib.

**Results:** Phenotype analysis found that hepatic autophagy, apoptosis, and mitochondrial injury were observed *in vivo* or *in vitro* after sunitinib treatment. By using spatial metabolomics and isolation of bile duct cells and LSECs, the zonated drug toxicity was observed around the portal vein. Hepatocytes, bile duct cells, and LSECs were damaged after sunitinib treatment. FXR inhibition and gut microbiota depletion aggravated sunitinib-induced liver injury. For diurnal variation, sunitinib-induced liver injury was enhanced at night compared with that at day, and FXR and gut microbiota participated in circadian rhythmic hepatotoxicity induced by sunitinib.

**Conclusions:** Our data suggested activation of FXR and *Parabacteroides distasonis* supplementation may be used to improve sunitinib-induced hepatotoxicity.

## Introduction

Sunitinib, a small-molecule tyrosine kinase inhibitor (TKI), was approved by the Food and Drug Administration (FDA) for the treatment of advanced renal cell carcinoma, and gastrointestinal stromal tumor in 2006. However, sunitinib was labeled as a black box warning by the FDA because of its severe and life-threatening liver injury as soon as it was put on the market. Liver function impairment (any alanine aminotransferase (ALT) elevation) was found in 23-40% tumor patients after sunitinib treatment 1, 2. Liver disorder, hepatitis, hepatic steatosis, hepatic failure, and hepatic cirrhosis happened after sunitinib treatment in clinical practice 2. The rate of grade 1 and grade 2 ALT elevation (ALT > 3 fold and > 3-5 fold, respectively) is higher (39-61% frequency) in clinical reports 3. Severe liver damage such as hepatic failure (0.3-5% frequency) was also induced by sunitinib in clinical reports with the increased aspartate aminotransferase (AST) and ALT in the thousands of units 4. The mechanism of sunitinib-induced hepatotoxicity is still obscure with a lack of effective intervention strategy.

The gut microbiome, the second largest genome of the host, is reported to impact the physiological function of liver, heart, immune cells, and brain. Various probiotics, such as *Parabacteroides distasonis* (*P. distasonis*) 5, *Bifidobacterium bifidum* (*B. bifidum*) 6, *Lactobacillus rhamnosus GG* 7, *Akkermansia muciniphila* 8 improved alcoholic and non-alcoholic liver disease or hepatic fibrosis in mice. Various gut microbiota-related metabolites were influenced by erlotinib (e.g., spermidine, ornithine, butyrate, and trimethylamine N-oxide), sorafenib (e.g., trimethyl-N-oxide and trimethylamine N-oxide), sunitinib (e.g., bile acid, hippuric acid, hydroxyindoleacetic acid, indoleacrylic acid, and indolelactic acid), anlotinib (e.g., indole) through metabolomics analysis 9. Gut microbiota also participated in the side-effect of various TKIs. The 16S rRNA or metagenomics analysis found that gut microbiota participated in sunitinib-, sorafenib-, regorafenib-, lenvatinib, and neratinib-induced diarrhea 10-14, sorafenib-induced hand-foot syndrome and skeletal muscle atrophy 11, 15 in mice or clinical reports. The relationship between gut microbiota and hepatotoxicity induced by TKIs has not been reported. Studying the role of gut microbiota in managing sunitinib-induced hepatotoxicity is still a fascinating topic.

The farnesoid X receptor (FXR) plays a vital role in bile acid and lipid homeostasis which has been recognized as an important target for drug-induced liver injury (DILI). FXR knockout mice aggravated acetaminophen (APAP)- and triptolide-induced hepatotoxicity and FXR agonist obeticholic acid (OCA) or GW4064 improved liver injury 16-18. Hepatic FXR decreased bile acid synthesis in a feedback mechanism via small heterodimer partner (SHP) 19. Activation of hepatic FXR can also induce the expression of bile salt export pump (BSEP) for the secretion of bile acids 19. Ileum FXR regulates the bile acid reabsorption process through activating organic solute transporter (OST), fibroblast growth factor 15 (FGF15), and ileal bile acid binding protein (IBABP) 19. FGF15 is secreted in the portal blood and signals to the liver to inhibit bile acid synthesis 19. The hepatic and ileum FXR inhibited hepatic bile acid synthesis. The relationship between FXR and TKIs is poorly studied.

In this study, spatial metabolomics and transmission electron microscopy (TEM) found that zonated drug toxicity induced by sunitinib was observed, and sunitinib impaired hepatocytes, bile duct cells, and liver sinusoids endothelial cells (LSECs) around the hepatic portal vein. Liver-specific Fxr-null (*Fxr^ΔL^*) mice aggravated sunitinib-induced liver injury indicating hepatic FXR-autophagy pathway played an important role in sunitinib-induced side effects. Gut microbiota depletion also aggravated sunitinib-induced liver injury, and *P. distasonis* and 3-oxolithocholic acid (3oxoLCA) improved sunitinib-induced liver injury. Finally, activation of FXR or *P. distasonis* supplementation may be used to improve sunitinib-induced liver injury.

## Methods

### Animals

Male FXR knockout mice (C57BL/6J background, 6-week-old, from Dr. Frank J. Gonzalez laboratory in National Cancer Institute (NCI), USA) and their wild-type mice (FXR+/+) were previously described 20. *Fxr^fl/fl^* mice were from Frank J. Gonzalez laboratory 21. Male 6-week- old wide-type C57BL/6J mice were purchased from the GemPharmatech Co., Ltd. (China). Mice were co-housed on a 12-hour light/dark cycle with standard water and rodent chow *ad libitum*. All animal experiments were approved by the Institutional Animal Care and Use Committee of the West China Hospital, Sichuan University.

To investigate the hepatotoxicity of sunitinib, mice were divided into three groups (n = 6): (1) control; (2) 75 mg/kg sunitinib; (3) 150 mg/kg sunitinib. Sunitinib groups were treated with sunitinib for 5 days (i.g., 75 and 150 mg/kg dissolved in physiologic saline solution) 1. To investigate the time-dependent effect, 1, 3, and 5 day animal samples were collected after 150 mg/kg sunitinib treatment (n = 5). The mice were killed at 24 h after sunitinib treatment.

FXR knockout mice and their wild-type mice (FXR+/+) were used to evaluate the effect of FXR on sunitinib-induced hepatotoxicity. Mice were divided into four groups (n = 6): (1) control; (2) sunitinib; (3) FXR-/- control; (4) FXR-/- sunitinib. Sunitinib and FXR-/- sunitinib groups were treated with 150 mg/kg sunitinib for 5 days. *Fxr^fl/fl^* mice, intestine-specific Fxr-null mice (*Fxr^ΔIE^*), and liver-specific Fxr-null mice (*Fxr^ΔL^*) were used to evaluate the effect of FXR on sunitinib-induced hepatotoxicity. Mice were divided into six groups (n = 6): (1) *Fxr^fl/fl^* control; (2) *Fxr^fl/fl^* sunitinib; (3) *Fxr^ΔL^* control; (4) *Fxr^ΔL^* sunitinib; (5) *Fxr^ΔIE^* control; (6) *Fxr^ΔIE^* sunitinib. Sunitinib groups were treated with 150 mg/kg sunitinib for 5 days.

To determine the role of gut microbiota in sunitinib-induced hepatotoxicity, mice were divided into four groups (n = 6): (1) control; (2) sunitinib; (3) antibiotics+sunitinib (A+Sun); (4) antibiotics+sunitinib+microbiota transplantation (A+Sun+Recon). The antibiotics treatment and fecal microbiota transplantation (FMT) were carried out based on previous study 5. Mice were treated with antibiotics for 7 days. FMT and 150 mg/kg sunitinib treatment were conducted for 5 days after antibiotics treatment.

To evaluate the effect of 3oxoLCA in sunitinib-induced liver injury, mice were divided into four groups (n = 6): (1) control; (2) 3oxoLCA; (3) sunitinib; (4) 3oxoLCA+sunitinib. 3oxoLCA and 3oxoLCA+sunitinib were treated with 3oxoLCA for 8 days (i.g., 10 mg/kg dissolved in corn oil) 22. After 3oxoLCA treatment for 3 days, mice were treated with 150 mg/kg sunitinib for 5 days. To investigate the role of isolithocholic acid (isoLCA) and 3oxoLCA in sunitinib-induced hepatotoxicity, mice were divided into three groups (n = 6): (1) sunitinib; (2) isoLCA+sunitinib; (3) 3oxoLCA+sunitinib. IsoLCA and 3oxoLCA groups were orally treated with isoLCA and 3oxoLCA for 8 days (i.g., 10 mg/kg dissolved in corn oil) 22. After isoLCA and 3oxoLCA treatment for 3 days, mice were treated with 150 mg/kg sunitinib for 5 days.

To investigate the role of *P. distasonis* and *B. bifidum* in sunitinib-induced hepatotoxicity, mice were divided into six groups (n = 6): (1) control; (2) sunitinib; (3) *P. distasonis*+sunitinib; (4) *B. bifidum*+sunitinib; (5) *P. distasonis*; (6) *B. bifidum*. Mice were treated with antibiotics for 7 days. The *P. distasonis* (ATCC8503) and *B. bifidum* (ATCC11863) were given for 2 weeks (i.g., 2×10^8^ CFU dissolved in PBS, once a day) after antibiotics treatment. In the last five days, mice were treated with 150 mg/kg sunitinib for 5 days. *P. distasonis* was cultured in brain-heart infusion liquid medium (Huankai, China). *B. bifidum* were cultured in reinforced Clostridial liquid medium (Hopebio, China).

To evaluate the role of diurnal variation in sunitinib-induced hepatotoxicity, mice were divided into four groups (n = 8): (1) control 0 h; (2) sunitinib 0 h; (3) control 12 h; (4) sunitinib 12 h. Mice were gavaged with 150 mg/kg sunitinib at 0 h (8:00 AM when the light is on-start of resting period) or 12 h (8:00 PM when the light is off-start of active period) for 5 days. To investigate FXR in the hepatotoxicity of diurnal variation, mice were randomly assigned into four groups (n = 5): (1) FXR-/- control 0 h; (2) FXR-/- sunitinib 0 h; (3) FXR-/- control 12 h; (4) FXR-/- sunitinib 12 h. FXR knockout mice were gavaged with 150 mg/kg sunitinib at 0 h (8:00 AM) or 12 h (8:00 PM) for 5 days. To investigate gut microbiota in the hepatotoxicity of diurnal variation, mice were divided into 4 group (n = 6): (1) control 0 h; (2) A+Sun 0 h; (3) control 12 h; (4) A+Sun 12 h. Mice were treated with antibiotics for 7 days. 150 mg/kg sunitinib treatment were conducted for 5 days at 0 h or 12 h after antibiotics treatment.

### Metagenomic analysis of microbiome data

Microbial genomic DNA was obtained using the Stool Genomic DNA kit (TIANGEN, China). Analysis was performed by the Beijing Genomics Institute (Shenzhen, China) with DNBSEQ platform. Copies of gut microbiota in cecum content were quantified by qPCR.

### Bacterial culture, growth curve and biofilm assay

*P. distasonis* (ATCC8503) and *B. bifidum* (ATCC11863) were obtained from American Type Culture Collection (ATCC) and cultured in anaerobic incubation system. *P. distasonis* was cultured in brain-heart infusion liquid medium (Huankai, China) and columbia blood agar base solid medium (Hopebio, China) with 5% sleep blood (Hopebio, China) for 48 h. *B. bifidum* were cultured in reinforced Clostridial liquid medium (Hopebio, China) and columbia blood agar base solid medium (Hopebio, China) with 5% sleep blood (Hopebio, China) for 48 h. Gram stain (BKMAN, China) was measured based on the manufacturer's instructions.

To evaluate the effect of sunitinib on the growth of *P. distasonis* and *B. bifidum*, 2% (v/v) medium containing *P. distasonis* (7.2×10^4^ CFU/μl) or *B. bifidum* (4×10^7^ CFU/μl) was added to medium containing sunitinib (0.44 μM-1.4 mM based on the maximum solubility and drug concentration in animal experiment) for 48 h. Absorbance value of growth curve was measured at 600 nm 5. Crystal violet was used to evaluate biofilm formation, and absorbance value of biofilm assay was measured at 590 nm 5. To determine that *P. distasonis* and *B. bifidum* had 3α hydroxysteroid dehydrogenase (3αHSDH) and 3βHSDH activity *in vitro*, lithocholic acid (LCA, 75 μM) or 3oxoLCA (75 μM) was added into the medium including 2% *P. distasonis* or *B. bifidum* for 48 h. LCA, isoLCA, and 3oxoLCA levels were evaluated by liquid chromatography-mass spectrometry (LC-MS) 23.

### UHPLC-Q Exactive plus MS analysis

Serum, liver, cecum content, duodenum, jejunum, and ileum samples were prepared based on previous study 5. The liquid chromatography system was a Thermo Fisher Vanquish Flex System combined with a T3 column (ACQUITY UPLC HSS, 100×2.1 mm, 1.8 μm, Waters, USA). The mobile phase was acetonitrile and H_2_O containing 0.1% formic acid, respectively. The flow rate was 0.3 ml/min. The eluent gradient was from 2% to 98% acetonitrile in a 17 min run. The Mass spectrum system was Thermo Fisher Vanquish UHPLC-Q Exactive plus MS. The parameters were set as follows: sheath gas and aux gas were 40 arb and 5 arb, respectively; spray voltage was 3.5 kV; capillary temperature and auxiliary gas heater temperature were 350 °C and 220 °C, respectively. Principal component analysis (PCA) was analyzed using SIMCAP 13.0 (Umetrics, Kinnelon, NJ). The chemical structures of the metabolites were searched in HMDB database and identified by MS/MS fragmentation in Table S2-S4.

### QPCR, western blot, immunohistochemical and immunofluorescence analysis

Primer sequence was shown in Table S5. The following antibodies were used: platelet endothelial cell adhesion molecule 1 (CD31, ab7388, Abcam, UK), CD31 (ab24590, Abcam, UK), cytokeratin 19 (CK19, ab254186, Abcam, UK), microtubule- associated protein light chain 3B (LC3B, ab192890, Abcam, UK), zonula occludens-1 (ZO-1, ab96587, Abcam, UK), FXR (NB400-153, Novus), FXR (sc-25309, Santa Cruz Biotechnology, USA), BSEP (PB0379, BOSTER), sequestonsome1 (SQSTM1/P62, ab109012, Abcam, UK), Caspase3 (ab184787, Abcam, UK). Sunitinib antofluorescence showed strong fluorescence with a maximum at 540 nm 24.

### Biochemical and histological assay

AST, ALT, alkaline phosphatase (ALP), diamine oxidase (DAO), malonaldehyde (MDA), total bile acid (TBA), total cholesterol (TC), total triglycerides (TG) (Nanjing Jiancheng Bioengineering Institute, China), cell autophagy detection assay kit (Solarbio, China), terminal deoxynucleotidyl transferase dUTP nick end labeling (TUNEL, Beyotime, China), mitochondrial membrane potential (Beytime, China), and 3-[4,5- dimethylthiazol-2-yl]-2,5-diphenyl tetrazolium bromide (MTT, Solarbio, China) were measured following the manufacturer's instructions. Evans blue (3 mg/ml in phosphate buffer saline, Ruitaibio, China) was intravenously injected into mice based on previous study 25. After 2 h, the tissues of mice including duodenum, jejunum, ileum, cecum content, colon, and liver were collected. Alcian blue (Leagene, China), H&E, and Oil red O staining were carried out.

### Laser speckle contrast imaging

RFLSI-ZW Laser Speckle Contrast Imaging System (RWD Life Science, China) was used to continuously record the intensity of microcirculatory blood flow in liver and intestinal tract (ileum, cecum, and colon) 26, 27. Mice (n = 3) were used to evaluate the effect of 150 mg/kg sunitinib (5 days) on blood flow.

### Clinical and RNA-Seq data from database

Clinical feces LC-MS data and RNA-Seq data were obtained from database in previous studies 5, 28. To evaluate the isoLCA and 3oxoLCA levels in the feces of liver injury patient, clinical feces LC-MS data in MetaboLights database (MTBLS6742) was re-analysis. The clinical feces samples of severe hepatic fibrosis patient (n = 17) and healthy people (n = 10) were included in MetaboLights database (MTBLS6742). The patient characteristics in MetaboLights database could be observed in our previous study 5. The study protocol was approved by the Conjoint Health Research Ethics Board of West China Hospital (ChiCTR2200067222). To evaluate the relationship between autophagy and FXR, RNA-Seq data from wild type and FXR knockout mice in Gene Expression Omnibus database (GSE216375) was re-analysis.

### Isolation of primary hepatocytes, bile duct cells, and liver sinusoidal endothelial cells

Primary hepatocytes were obtained from mouse liver tissue by two-step collagenase perfusion (hanks and 2 g/l collagenase IV, 10 min). Primary hepatocyte was cultured in William's Medium E (Gibco, USA) containing 10% fetal bovine serum (FBS) and 1% penicillin/streptomycin. Bile duct cells were obtained from mouse liver tissue by two-step collagenase perfusion (3 g/l collagenase IV, 10 min) 29, 30. Bile duct was removed, washed with medium and digested with 20 U/ml DNase I and 0.5 g/l collagenase IV for 40 min in cell incubator. Bile duct cells were cultured in DMEM/F12 medium containing 20% FBS, 10 ng/ml epidermal growth factor (EGF, GenScript, China), and 1% penicillin/streptomycin. Bile duct cells were identified with CK19 antibody. LSECs were obtained based on previous study 31. LSECs were isolated by collagenase perfusion (2 g/l collagenase IV, 10 min), percoll gradient isopycnic centrifugation (25% and 50% percoll), and selective adherence based on adhesion time. Through selective adherence, Kupffer cells were isolated from LSECs. LSECs were cultured in DMEM containing 15% FBS, 10 ng/ml endothelial cell growth factor (ECGF, macgene, China), and 1% penicillin/streptomycin. Primary hepatocytes were harvested 24 h after incubation with sunitinib (2.5-40 μM) 32, 3-methyladenine (3-MA, 0.0097-2.5 mM) 33, 34, NH_4_Cl (0.0097-2.5 mM) 33, isoLCA (6.25 μM), and 3oxoLCA (6.25 μM) 23. Primary bile duct cells were harvested 24 h after incubation with sunitinib (10, 20, and 40 μM). Primary LSECs were harvested 24 h after incubation with 10, 20, and 40 μM sunitinib.

### Cell culture and treatment

HepG2 cells (ATCCHB-8065) were maintained in DMEM containing 10% FBS and 1% penicillin/streptomycin. HepG2 cells were exposed with 10, 20, and 40 μM sunitinib for 24 h. Human umbilical vein endothelial cells (HUVECs) were maintained in endothelial cell medium (Shanghai Zhong Qiao Xin Zhou Biotechnology Co., Ltd, China) containing ECGF, 5% FBS, and 1% penicillin/streptomycin. HUVECs were exposed with 10, 20, and 40 μM sunitinib for 24 h.

### Transmission electron microscopy (TEM)

Mice liver after 150 mg/kg sunitinib treatment (5 days) was fixed in phosphate buffer (0.1 M, 1% osmium tetroxide and 3% glutaraldehyde). The liver was dehydrated in series acetone after rinsing in water. Then the liver was embedded in SPI-Pon812. Ultrathin sections were attached to copper grids. Sections were strained with methylene blue. Ultrathin sections were cut with diamond knife, stained with uranyl acetate and lead citrate. TEM was observed using a JEOL transmission electron microscope (JEM-1400-FLASH).

### Luciferase assays

The plasmids renilla-luciferase, FXR, tk-EcRE-luciferase were transfected into HepG2 cells using Lipofectamine 2000 (Invitrogen, Grand Island, NY) 5. HepG2 cells were treated with chenodeoxycholic acid (CDCA, a FXR agonist, 50 μM), isoLCA (50 μM), and 3oxoLCA (50 μM) for 24 h. Luciferase activities were measured with multimode plate reader (EnVision 2105, Perkin Elmer, USA).

### Spatial metabolomics study

The frozen liver tissue was cut to a 10 μm thickness and dried. Mass spectrometry imaging (MSI) experiments were performed using an air flow-assisted desorption electrospray ionization (AFADESI) platform coupled to a Q-Orbitrap mass spectrometer (Q Exactive, Thermo Scientific, Waltham, Mass). The mass spectrometry data was analyzed by Massimager Pro (Version 1.0, Beijing, China) 35. MSI has been used to identify various endogenous metabolites (e.g., bile acids taurocholic acid (TCA), sulfolithocholylglycine, and taurodeoxycholic acid (TDCA)) and xenobiotic metabolites (e.g., olanzapine) 36-38.

### Molecular docking

Schrödinger 2021, Maestro version 12.8 (Schrödinger LLC, New York, USA), was used for molecular docking to elucidate the affinities between CDCA (FXR agonist), isoLCA, 3oxoLCA and FXR. The crystal structure of FXR (PDB ID: 6HL1) was obtained from the protein data bank. Three-dimensional structure of compound was obtained from Pubchem (isoLCA CID164853, 3oxoLCA CID5283906, and CDCA CID10133).

### Statistics

MetaboAnalyst 4.0 was used to evaluate pathway enrichment. Data analyses were performed with GraphPad Prism 8 (GraphPad, USA), ImageGP (a data visualization web), and OriginPro 9.0 (OriginLab, USA). Differences among multiple groups were tested with one-way ANOVA followed by Dunnett's post hos comparisons. Differences between two groups were tested with the Student's t-test.

## Results

### Sunitinib caused autophagy, apoptosis and mitochondrial dysfunction *in vivo* or *in vitro*

**Figure 1:**
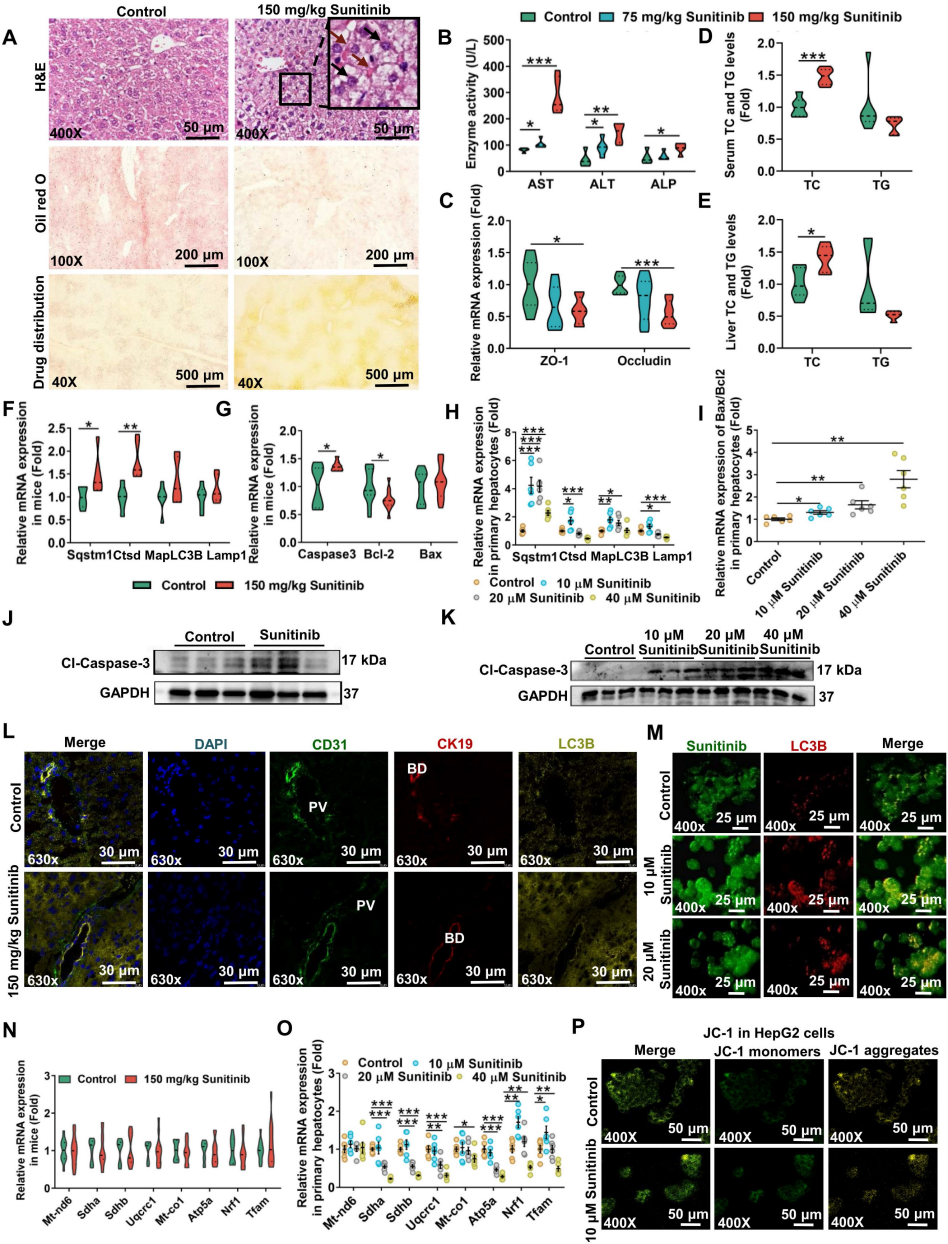
Sunitinib caused autophagy, apoptosis, and mitochondrial dysfunction. (**A**) H&E staining, Oil red O staining, and drug distribution in mouse liver. Red arrow: bleeding; Black arrow: nuclear shrinkage. (**B**) Serum AST, ALT, and ALP enzyme activities. (**C**) Tight junction gene expression in mouse liver. (**D**) TC and TG levels in mouse serum. (**E**) TC and TG levels in mouse liver. (**F-G**) Autophagy (left, **F**) and apoptosis (right, **G**) gene expression in mouse liver. (**H-I**) Autophagy (left, **H**) and apoptosis (right,** I**) gene expression in mouse primary hepatocytes. (**J-K**) Cl-caspase-3 protein expression in mouse liver (**J**) and in mouse primary hepatocytes (**K**). Cl-Caspase-3 was the active form of the protein. (**L**) Co-staining CD31 (marker of LSECs), CK19 (marker of bile duct cells), and LC3B protein level in mouse liver. BD: bile duct; PV: portal vein. (**M**) Co-localization of sunitinb and LC3B protein in mouse primary hepatocytes. Sunitinib antofluorescence showed strong fluorescence with a maximum at 540 nm. (**N-O**) Mitochondrial respiratory chain gene expression in mouse liver (**N**) and mouse primary hepatocytes (**O**). (**P**) JC-1 staining of HepG2 cells after 10 μM sunitinib treatment for 24 h. Yellow fluorescence represented JC-1 aggregates in healthy mitochondria, while green fluorescence represented mitochondrial membrane potential collapse. *P < 0.05, **P < 0.01, ***P < 0.001.

Drug concentration was positively correlated with degree of injury. The yellow sunitinib has been observed in skin (37%) in clinical reports 39. Drug distribution showed sunitinib was accumulated around hepatic portal vein in mice after giving 150 mg/kg sunitinib for 5 days (Figure 1A). Sunitinib induced liver injury as revealed by bleeding and nuclear shrinkage in mouse liver (Figure 1A), decreased hepatic lipid accumulation (Figure 1A), increased serum AST, ALT, and ALP enzyme activities (Figure 1B), decreased tight junction gene expression (ZO-1 and occludin) in liver (Figure 1C), increased TC in serum and liver (Figure 1D-E), decreased body weight, and increased hepatic MDA level (Figure S1A-B). Sunitinib-induced liver injury was dose-dependent and time-dependent (Figure 1B and Figure S2A-B). Sunitinib also decreased cell viability and increased AST and ALT levels in mouse primary hepatocytes and HepG2 cells (Figure S1C-D). These results showed that sunitinib induced hepatotoxicity *in vivo* and *in vitro*.

**Figure 2:**
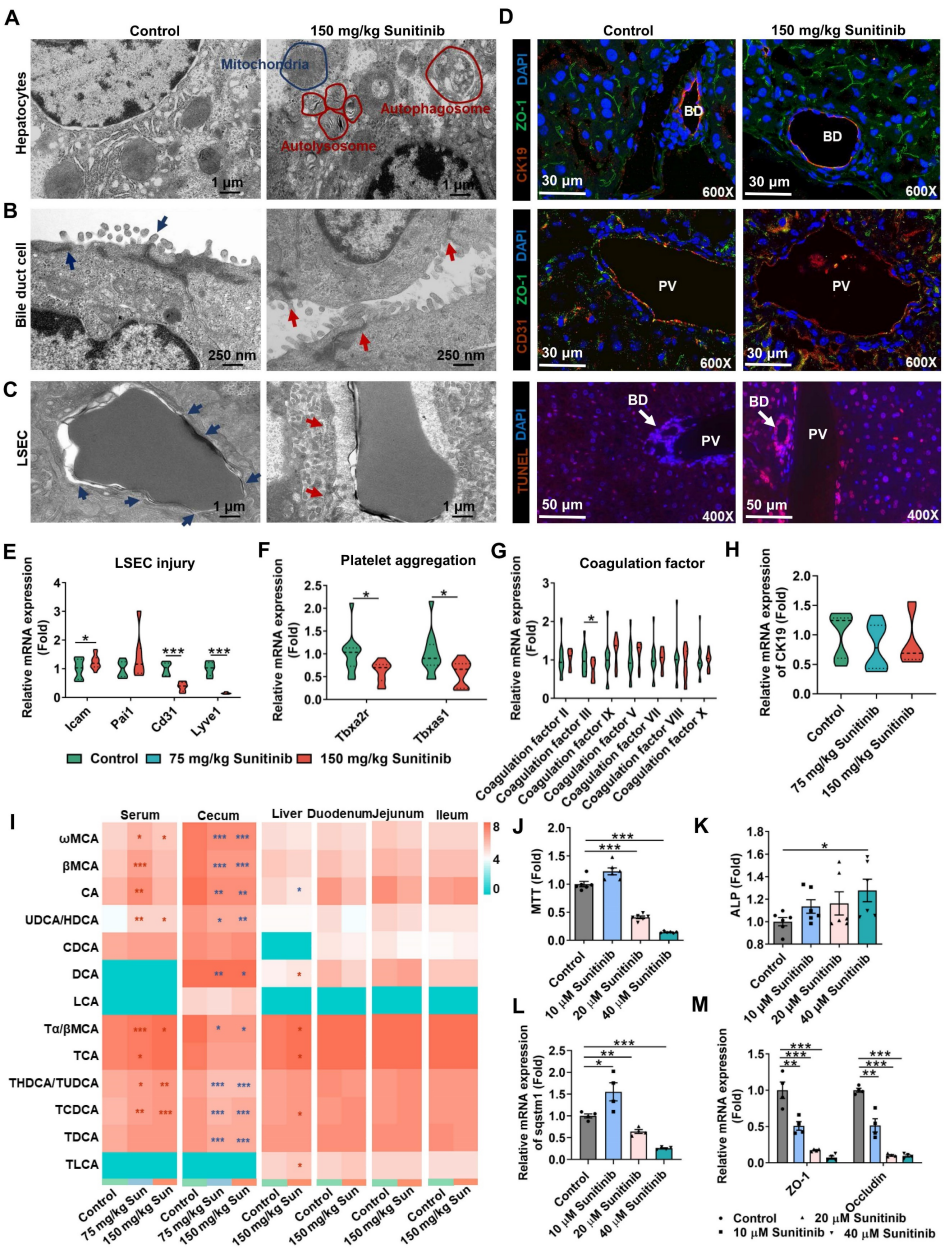
Sunitinib induced mouse bile duct cell and LSEC injury. (**A**) TEM analysis of hepatocyte toxicity. Autophagosome and autolysosome were labeled in sunitinib group. (**B**) TEM analysis of bile duct cell toxicity. Intercellular space was increased in sunitinib group (red arrow). (**C**) TEM analysis of LSEC toxicity. LSECs from sunitinib group showed a loss of fenestrae in comparison with healthy mice (arrow). (**D**) Co-staining of CK19 (marker of bile duct cells) and ZO-1 (up), CD31 (marker of LSECs) and ZO-1 (middle) in mouse liver. BD: bile duct; PV: portal vein. TUNEL staining (down) in mouse liver. (**E**) Vascular injury gene expression in mouse liver. (**F**) Platelet aggregation gene expression in mouse liver. (**G**) Coagulation factor gene expression in mouse liver. (**H**) CK19 gene expression, marker of bile duct cells, in mouse liver. (**I**) Serum, cecum content, liver, duodenum, jejunum, ileum bile acid levels after sunitinib treatment. (**J-M**) Cell viability (**J**), ALP level (**K**), autophagy gene expression (**L**), and tight junction gene expression (**M**) in primary bile duct cells. *P < 0.05, **P < 0.01, ***P < 0.001.

Sunitinib-induced autophagy was observed in mouse liver and in mouse primary hepatocytes. Autophagy gene expression was increased in mouse liver (*Sqstm1* and cathepsin D (*Ctsd*)) and in mouse primary hepatocytes (*Sqstm1*, *Ctsd*, and microtubule associated protein 1 light chain 3 beta (*MapLC3b*)) after sunitinib treatment (Figure 1F, H). Immunofluorescence staining showed that LC3B protein (marker of autophagy) was increased in hepatocytes, bile duct cells, and LSECs of mouse liver (Figure 1L), and sunitinib also decreased CK19 (marker of bile duct cells) and CD31 (marker of LSECs) fluorescence partially (Figure 1L). Autolysosome and autophagosome were also revealed by TEM in mouse hepatocytes (Figure 2A). Autophagy was co-localized with the sunitinib autofluorescence in 10 and 20 μM sunitinib-treated primary hepatocytes (Figure 1M), which was consistent with APAP-induced hepatotoxicity through co-locating APAP and autophagy 40. Sunitinib antofluorescence in cells has been observed in previous studies 41, 42.

Sunitinib-induced apoptosis was observed in mice liver and *in vitro* (Figure 1G, I-K and Figure S1F-G). The increased *Caspase3* and the decreased B-cell lymphoma-2 (*Bcl2*) gene expression and the increased Cl-Caspase-3 protein level showed apoptosis happened in mouse liver (Figure 1G, J), which was consistent with regorafenib-induced hepatotoxicity 43. Apoptosis was also observed in mouse primary hepatocyte after sunitinib treatment (Figure 1I, K and Figure S1F-G). Nuclear shrinkage was also observed by TEM in mouse hepatocytes apart from H&E staining (Figure 1A and Figure 2A).

Sunitinib-induced mitochondrial injury was observed in mouse primary hepatocytes and HepG2 cells. Changed mitochondrial respiratory chain gene expression and JC-1 staining revealed that sunitinib induced mitochondrial injury in mouse primary hepatocytes and HepG2 cells (Figure 1O-P). But mitochondrial respiratory chain gene expression was unchanged in mouse liver (Figure 1N). Furthermore, inflammatory factor, pyroptosis, ferroptosis, and fibrosis were not affected by sunitinib in mouse liver (Figure S1H).

Sunitinib was accumulated in mouse ileum compared with duodenum, jejunum, and colon (Figure S3A). Sunitinib induced weak intestinal injury as revealed by the increased serum DAO activity (an indicator of the intestinal mechanical barrier damage) and the decreased *Cd31* and lymphatic vessel endothelial hyaluronan receptor 1 (*Lyve1*) gene expressions (indicator of vascular damage) in mouse ileum (Figure S3B-C). Vascular leakage was examined by Evans blue staining (Figure S3D-F). Leakage of Evans blue was observed into the intestine and liver tissues (Figure S3D-F). Goblet cells, secreting mucus, are important for maintaining the mucosal barrier. Alcian blue staining showed that sunitinib decreased ileum goblet cells (Figure S3G). These results showed that sunitinib induced weak intestinal injury.

### Sunitinib impaired LSECs and bile duct cells *in vivo* and *in vitro*

The accumulation of the yellow drug sunitinib *in vitro* has been observed in previous study 42. Drug distribution of sunitinib *in vivo* was also observed, and sunitinib was scattered around LSECs and bile duct in mouse liver (Figure 1A). The yellow color of sunitinib observed *in vitro* and *in vivo* come from its strong light-absorption (340 nm-480 nm) and fluorescence emission (540 nm) 24. At the same time, bleeding was observed through H&E staining implying the impaired LSECs (Figure 1A). Therefore, we concluded that LSECs and bile duct in mouse liver were injured.

Sunitinib induced LSEC injury in mouse liver. TEM imaging analysis showed that, compared with controls, there are less fenestrae among LSECs in the liver of mice treated with sunitinib (Figure 2C, arrow). Co-staining of CD31 (marker of LSECs) and ZO-1 in mouse liver showed that tight junction of LSECs was decreased after sunitinib treatment (Figure 2D and Figure S4B). TUNEL staining showed that apoptosis happened around the LSECs in mouse liver (Figure 2D). Intercellular adhesion molecule (*Icam*) and plasminogen-activated inhibitor-1 (*Pai1*) gene expressions were increased and *Cd31* and *Lyve1* gene expressions were decreased in mouse liver also indicating the impaired LSECs (Figure 2E). Furthermore, hepatic platelet aggregation gene expressions (thromboxane A2 receptor (*Tbxa2r*) and thromboxane-A synthase 1 (*Tbxas1*)) and coagulation factor III gene expressions were decreased indicating coagulation system was inhibited by sunitinib (Figure 2F-G). Sunitinib also decreased cell viability, increased autophagy (*Sqstm1*), and changed vascular injury gene expression (*Icam* and *Cd31*) in mouse primary LSECs (Figure S5A-C) and HUVECs (Figure S5D-F). Laser speckle contrast imaging showed that the blood flow volume was not affected by sunitinib in mouse liver, and sunitinib increased blood flow volume in mouse ileum (Figure S5H) which might be consistent with the side effect (hypertension) of sunitinib in clinical reports 44. All this result showed that sunitinib induced LSEC injury and impaired coagulation system and blood circulation system partially.

Sunitinib also induced bile duct cell injury in mouse liver. Results of TEM analysis showed that the intercellular space of bile duct cells was increased due to the loosed tight junction in the sunitinib group (Figure 2B). Co-staining of CK19 (marker of bile duct cells) and ZO-1 in mouse liver also showed that the tight junction of bile duct cells was weakly decreased (Figure 2D and Figure S4A). TUNEL staining showed that apoptosis happened around the bile duct cells in mouse liver (Figure 2D). CK19 gene expression was unchanged (Figure 2H). Serum and hepatic bile acids were increased and cecum content bile acids were decreased implying cholestasis (Figure 2I). Mouse primary bile duct cells were separated and identified by CK19 (Figure S5G). Sunitinib decreased cell viability, increased ALP level in supernatant, induced autophagy (*Sqstm1* gene expression), and decreased tight junction in primary bile duct cells (Figure 2J-M). All these results showed that sunitinib impaired bile duct cells.

**Figure 3:**
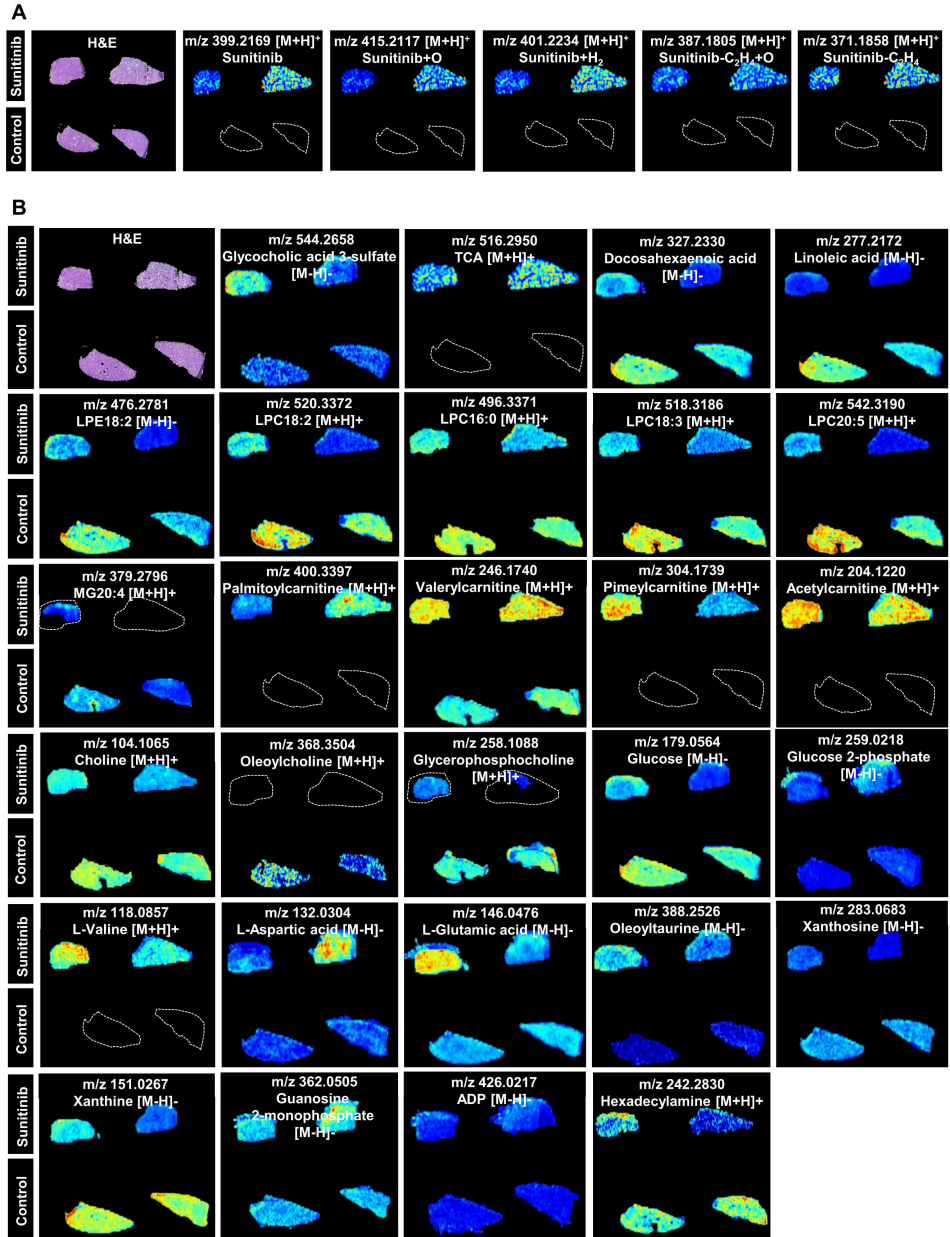
AFADESI-based visualization of metabolite distribution in mouse liver tissue. (**A**) Xenobiotic metabolites (sunitinib and its metabolites). (**B**) Endogenous metabolites.

Spatial metabolomics analysis presented the region-specific distribution for various metabolites, and this was consistent with the impaired LSECs and bile duct (Figure 3). Sunitinib and its xenobiotic metabolites (sunitinib+O, sunitinib+H_2_, sunitinib-C_2_H_4_+O, sunitinib-C_2_H_4_) showed region-specific distribution (Figure 3A). The spatial metabolomics analysis found that various endogenous metabolites were changed including bile acid (glycocholic acid 3-sulfate and TCA), lipid (docosahexaenoic acid, linoleic acid, lysophosphatidylethanolamine18:2 (LPE18:2), lysophosphatidyl-choline18:2 (LPC18:2), LPC16:0, LPC18:3, LPC20:5, and monoglyceride20:4 (MG20:4)), acylcarnitine (palmitoylcarnitine, valerylcarnitine, pimeylcarnitine, and acetylcarnitine), choline (choline, oleoylcholine, and glycerophosphocholine), glucose (glucose and glucose 2-phosphate), and amino acid (L-valine, L-aspartic acid, L-glutamic acid, and oleoyltaurine) (Figure 3B and Figure S6A) which was consistent with the result obtained through metabolomics analysis in mouse liver (Figure S6B-C and Figure S7).

### FXR-autophagy pathway participated in sunitinib-induced liver injury

**Figure 4:**
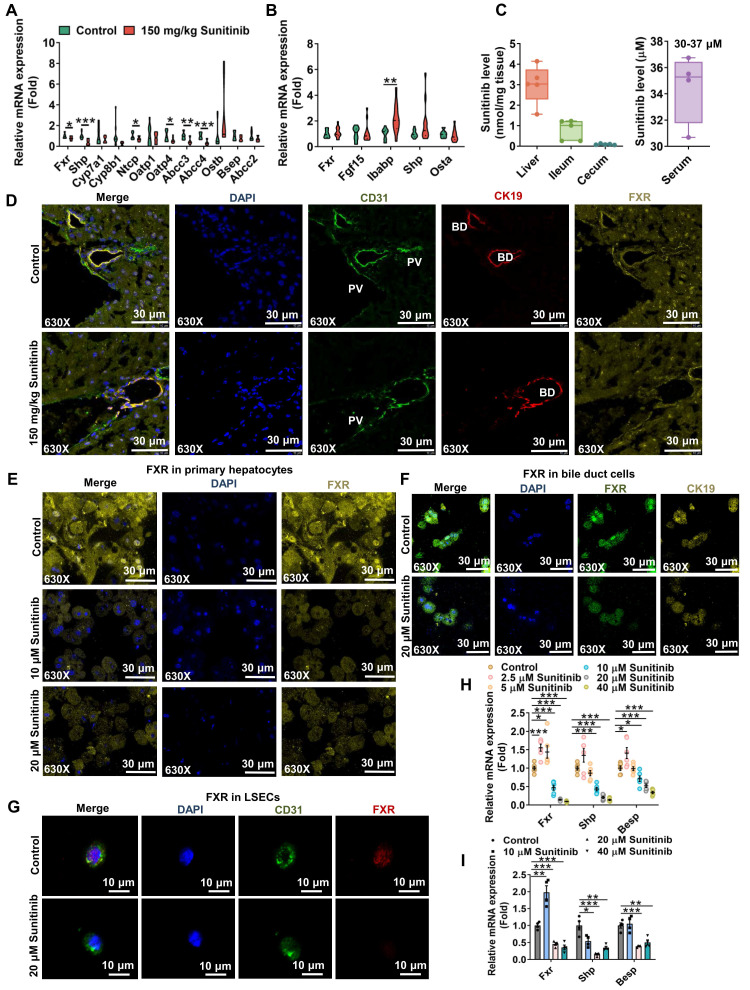
Hepatic FXR pathway was inhibited after sunitinib treatment *in vivo* and *in vitro*. (**A**) FXR- and bile acid-related gene expression in mouse liver. (**B**) FXR-related gene expression in mouse ileum. (**C**) Sunitinib level in mouse liver, ileum, cecum content, and serum. (**D**) Co-staining of CD31 (marker of LSECs), CK19 (marker of bile duct cells) and FXR in mouse liver. (**E**) FXR protein level in mouse primary hepatocytes. (**F**) Co-staining of FXR and CK19 in primary bile duct cells. (**G**) Co-staining of CD31 and FXR in primary LSECs. (**H**) FXR-related gene expression in mouse primary hepatocytes. (**I**) FXR-related gene expression in primary bile duct cells. *P < 0.05, **P < 0.01, ***P < 0.001.

**Figure 5:**
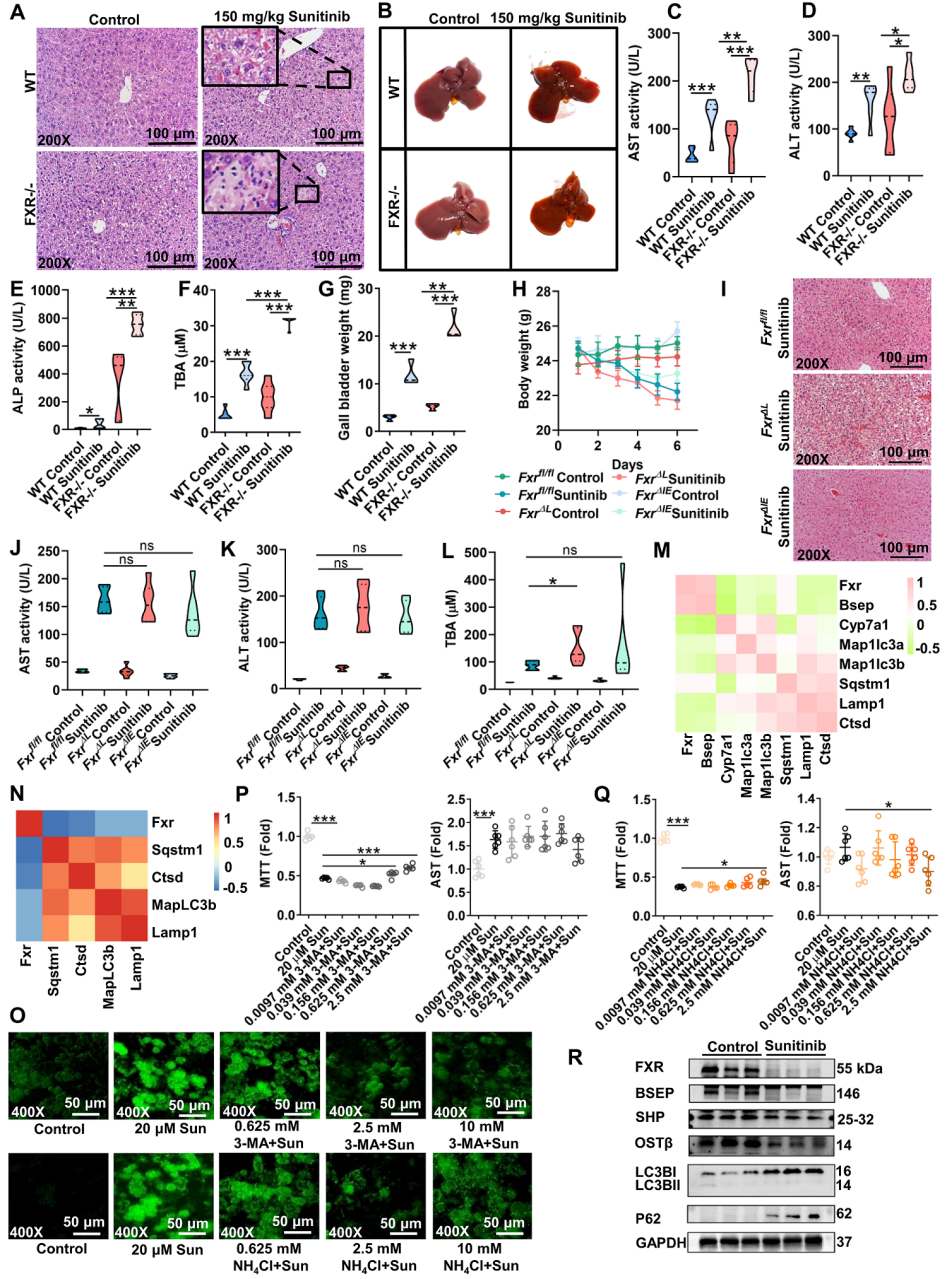
FXR-autophagy pathway participated in sunitinib-induced hepatotoxicity. (**A**) H&E staining of mouse liver. (**B**) Representative image of liver. (**C-G**) Serum AST (**C**), ALT (**D**), ALP (**E**), TBA (**F**), and gall bladder weight (**G**) in FXR knockout mice. (**H-L**) Body weight (**H**), H&E staining of mouse liver (**I**), AST (**J**), ALT (**K**), and TBA (**L**) in *Fxr^fl/fl^*, *Fxr^ΔL^*, and *Fxr^ΔIE^* mice. (**M**) Correlation analysis between FXR and autophagy gene expression through RNA-Seq analysis in wide type and FXR knockout mice. (**N**) Correlation analysis between FXR and autophagy gene expression in mouse liver. (**O**) Autophagy level using cell autophagy detection assay kit after 3-MA and NH_4_Cl treatment. (**P**) Cell viability and AST level after 3-MA treatment in mouse primary hepatocytes. (**Q**) Cell viability and AST level after NH_4_Cl treatment in mouse primary hepatocytes. (**R**) FXR (FXR, BSEP, SHP, and OSTβ) and autophagy (LC3BI/II and P62) protein expression in mouse liver after sunitinib treatment. *P < 0.05, **P < 0.01, ***P < 0.001.

With the increase of bile acid in serum and liver, bile acid-related gene expression was evaluated in mouse liver after 3 days and 5 days of sunitinib treatment (Figure 4A-B and Figure S2C). Sunitinib inhibited hepatic *Fxr* and its target gene *Shp* rather than ileum FXR pathway (*Fgf15*, *Ibabp*, *Shp* and *Ostα* in ileum mucosa), because sunitinib was mainly accumulated in liver (Figure 4A-C). Co-staining of CK19 (marker of bile duct), CD31 (marker of LSECs) and FXR showed that sunitinib decreased FXR protein levels in hepatocytes, bile ducts, and LSECs in mice liver (Figure 4D). Sunitinib also decreased FXR protein and gene expression in mouse primary hepatocytes, primary bile duct cells, and primary LSECs (Figure 4E-I). FXR knockout mice aggravated sunitinib-induced liver injury as shown by H&E staining, image of liver (bigger gall bladder), serum AST, ALT, ALP, TBA levels, and gall bladder weight (Figure 5A-G). *Fxr^ΔL^* mice aggravated sunitinib-induced liver injury as shown by body weight, H&E staining, and serum TBA levels compared with *Fxr^ΔIE^* mice (Figure 5H-L). The inhibition effect of sunitinib on FXR was not direct, and it may come from the toxicity effect as sunitinib increased FXR level at low dose in primary hepatocytes (2.5 μM, Figure 4H), bile duct cells (10 μM, Figure 4I) or mouse ileum (Figure 4B). These results showed that FXR inhibition aggravated sunitinib-induced liver injury.

FXR inhibited autophagy through direct and indirect ways: firstly, FXR bound to sites in autophagic gene promoters and inhibited autophagy 45; secondly, FXR trans-repressed autophagy genes by disrupting the functional cAMP response element binding protein (CREB) 46. Previous study found that FXR knockout and BSEP (a FXR target gene) knockout increased autophagy 45, 47. We predict that the inhibited FXR pathway increase autophagy in sunitinib group. As expected, FXR pathway gene expression (*Fxr* and *Bsep*) was negative correlation with autophagy pathway gene expression (*Map1lc3a*, *Map1lc3b*, *Sqstm1*, lysosomal-associated membrane protein 1 (*Lamp1*), and *Ctsd*) through transcriptomics analysis in FXR knockout and WT mice (Figure 5M). *Fxr* gene expression was negative correlation with autophagy pathway gene expression (*Sqstm1*, *Ctsd*, *MapLC3b*, and *Lamp1*) after sunitinib treatment in mouse liver (Figure 5N). Autophagy inhibitors 3-MA and NH_4_Cl improved sunitinib-induced cell injury in mouse primary hepatocytes (Figure 5O-Q). The protein levels of FXR and autophagy were also evaluated in Figure 5R. These results showed that FXR-autophagy pathway participated in sunitinib-induced liver injury.

### Gut microbiota participated in sunitinib-induced liver injury

**Figure 6:**
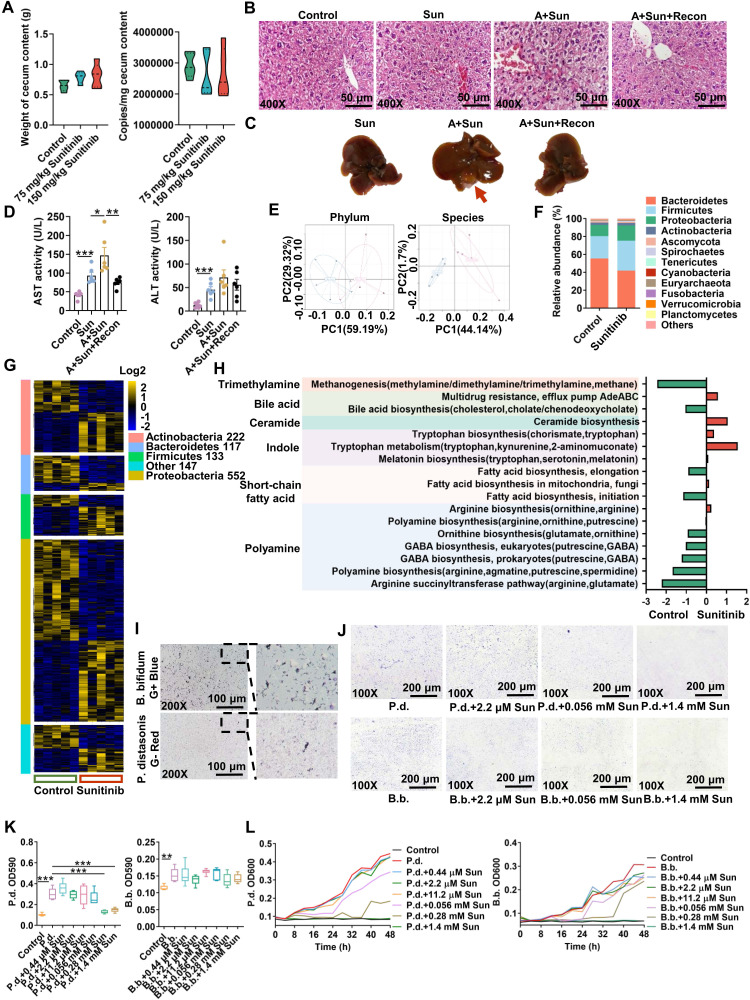
Gut microbiota participated in sunitinib-induced liver injury. (**A**) Weight of cecum content and copies of gut microbiota in mouse cecum content. (**B**) H&E staining. (**C**) Representative image of liver. (**D**) Serum AST and ALT enzyme activities. (**E**) PCA score plot of gut microbiota through Metagenomic analysis. (**F**) Relative abundance of phylum in the mouse cecum content. (**G**) 1171 species in the mouse cecum content. (**H**) Metagenomic pathway analysis. (**I**) Gram stain for *P. distasonis* and *B. bifidum*. (**J-K**) Biofilm formation of *P. distasonis* and *B. bifidum* was shown by microscopy stained with crystal violet (**J**). OD value stained with crystal violet (**K**). (**L**) Growth curve of *P. distasonis* and *B. bifidum* for 48 h in anaerobic incubator. *P < 0.05, **P < 0.01, ***P < 0.001.

**Figure 7:**
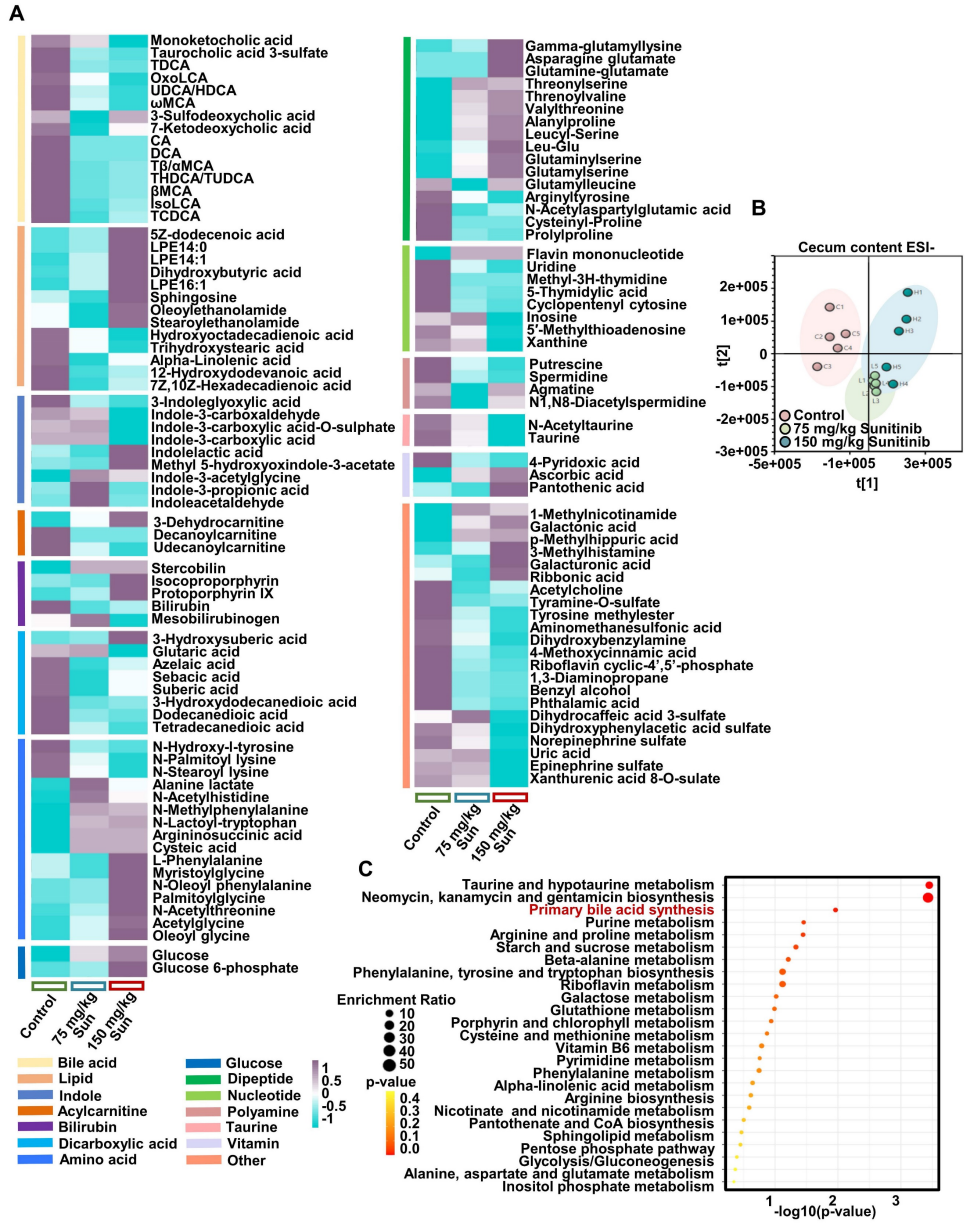
Changed metabolites and pathways in cecum content using metabolomics. (**A**) Changed metabolites in cecum content using non-target metabolomics analysis. (**B**) PCA score plot for cecum content metabolome in ESI- model. (**C**) Metabolomics pathway analysis using MetaboAnalyst 4.0.

As gut microbiota participated in sunitinib-induced diarrhea 10, we hypothesized that gut microbiota may play a vital role in sunitinib-induced liver injury. As expected, antibiotic aggravated sunitinib induced liver injury as shown by increased bleeding and nuclear shrinkage through H&E staining, bigger gall bladder, and increased serum AST level (Figure 6B-D). Fecal microbiota transplantation improved liver injury as shown by H&E staining and decreased AST level (Figure 6B-D). Metabolomics was carried out for mouse cecum content after sunitinib treatment (Figure 7). Various metabolites were changed including bile acid, lipid, indole, acylcarnitine, bilirubin, dicarboxylic acid, amino acid, glucose, dipeptide, nucleotide, polyamine, taurine, and vitamin (Figure 7A). Pathway analysis for metabolomics data was carried out and bile acid pathway was changed in cecum content in a forward position (Figure 7C).

Metagenomics analysis was carried out for mouse cecum content, and sunitinib decreased *Bacteroidetes* and increased *Firmicutes* (Figure 6E-F). In clinical reports, sunitinib and neratinib treatment decreased *Bacteroidetes* 10, 14. 1171 out of 6230 species were changed after sunitinib treatment (Figure 6G). The changed metabolic pathways included trimethylamine, bile acid, ceramide, indole, short-chain fatty acid, polyamine through metagenomics analysis (Figure 6H). Through metabolomics and metagenomics analysis, bile acid synthesis was inhibited in cecum content after sunitinib treatment (Figure 6H and Figure 7C). Serum and hepatic metabolomics analysis also found that bile acid pathway was influenced after sunitinib treatment (Figure S6C and S8B).

### Biological activity of 3oxoLCA through activating ileum FXR and inhibiting pathogens

**Figure 8:**
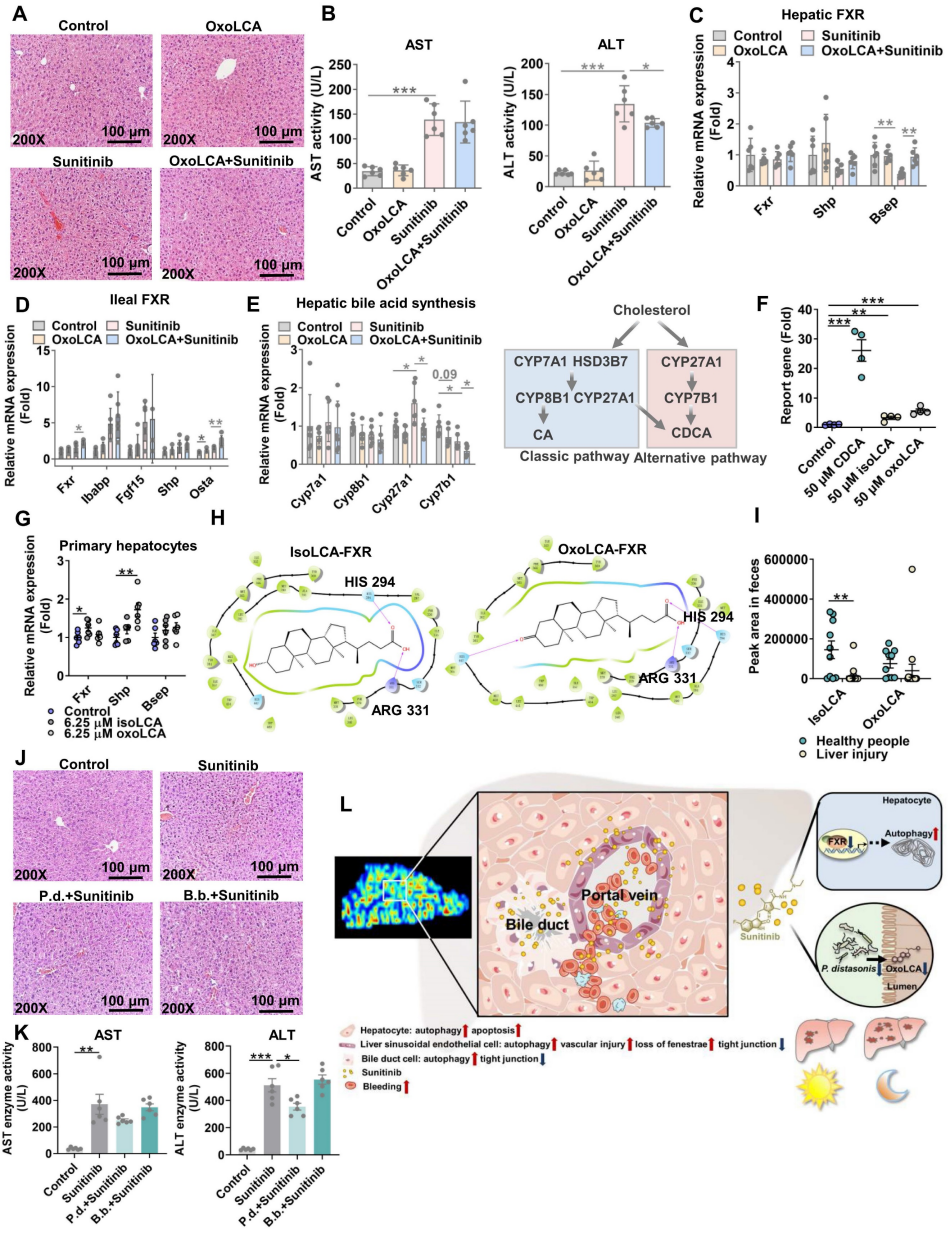
3oxoLCA regulated ileum FXR and hepatic bile acid synthesis. (**A**) H&E staining and representative image of liver. (**B**) Serum AST and ALT levels. (**C**) FXR gene expression in mouse liver. (**D**) FXR gene expression in mouse ileum. (**E**) Bile acid synthesis gene expression in mouse liver. Bile acid synthesis included classic pathway and alternative pathway. (**F**) Luciferase assay of the activation of FXR in HepG2 cells using FXR agonist CDCA, isoLCA, and 3oxoLCA treatment. (**G**) IsoLCA and 3oxoLCA activated FXR in mouse primary hepatocytes. (**H**) Docking showed that isoLCA and 3oxoLCA enter into the human FXR ligand-binding pocket. (**I**) IsoLCA and 3oxoLCA levels in the feces of clinical liver injury patients. (**J**) H&E staining of liver. (**K**) Serum AST and ALT levels. (**L**) Through spatial metabolomics study, sunitinib induced bleeding and spatially injured LSECs, bile duct cells and hepatocytes around the hepatic portal vein because the concentration of sunitinib is heterogeneity in mouse liver. FXR inhibition and gut microbiota depletion aggravated sunitinib-induced liver injury. Sunitinib-induced liver injury was enhanced at 12 h compared with 0 h, and FXR and gut microbiota also participated in circadian rhythmic hepatotoxicity. *P < 0.05, **P < 0.01, ***P < 0.001.

As bile acid synthesis pathway was followed with interest through metabolomics and metagenomics, the decreased gut-residing bacteria produce isoLCA and 3oxoLCA was focused in mouse cecum content after sunitinib treatment because of its antibacterial and anti-inflammation activity (Figure 7A). The decreased isoLCA and 3oxoLCA were also observed in the feces of clinical liver injury patients (Figure 8I). 3oxoLCA showed weaker protective effect for sunitinib-induced liver injury compared with isoLCA (Figure S9A). 3oxoLCA improved sunitinib-induced liver injury as shown by H&E staining and decreased serum ALT level (Figure 8A-B). The biological activity of 3oxoLCA and isoLCA may come from activating ileum FXR rather than liver FXR, as only a small quantity of 3oxoLCA could be detected in mouse liver (Figure 8C-D and Figure S9B). Then the activated ileum FXR inhibited bile acid synthesis (alternative pathway, cytochrome P450 27A1 (*Cyp27a1*) and oxysterol 7-alpha hydroxylase (*Cyp7b1*)) and finally decreased bile acid level in serum (Figure 8E and Figure S9B). IsoLCA and 3oxoLCA activated FXR pathway directly: luciferase reporter gene assays revealed that isoLCA and 3oxoLCA activated FXR signaling (Figure 8F); in mouse primary hepatocytes, isoLCA and 3oxoLCA increased *Fxr* mRNA and target gene mRNA *Shp* after a 24 h exposure (Figure 8G); molecular docking revealed that 3oxoLCA, isoLCA, and CDCA were found in the pocket of FXR, and hydrogen bonding was formed between 3oxoLCA, isoLCA, or CDCA and amino acid residues (His294 and Arg331) at the FXR ligand-binding site (Figure 8H and Figure S9C). Furthermore, 3oxoLCA also inhibited gram-positive pathogens *Streptococcus uberis* (Figure S9D), and previous study found that 3oxoLCA and isoLCA inhibited 9 gram-positive pathogens including *Clostridium difficile*, *Streptococcus dysgalactiae*, and *Streptococcus pyogenes* 48. Therefore, it indicated that 3oxoLCA protected sunitinib-induced liver injury through activating ileum FXR and inhibiting gram-positive pathogens.

### 3oxoLCA-producting bacteria *P. distasonis* improved sunitinib-induced liver injury

*P. distasonis* could produce isoLCA and 3oxoLCA in previous studies and in our experiment (Figure S10A-C) 22, 23, 48. Furthermore, *B. faecale*, *B. longum*, *B. pseudocatenulatum* could produce isoLCA and 3oxoLCA 23, 49. It was predicted that the decreased *B. bifidum* after sunitinib treatment through metagenomics analysis may produce isoLCA and 3oxoLCA (Figure 6G). 3oxoLCA was positively correlated with *P. distasonis* and *B. bifidum* (Figure S10E). Therefore, *P. distasonis* and *B. bifidum* were chosen and identified in our study (Figure 6I). Qualitative and quantitative experiments revealed that sunitinib inhibited biofilm formation *in vitro* (Figure 6J-K). *In vitro* analysis found that sunitinib from 0.28 mM to 1.4 mM effectively inhibited the growth curve of *P. distasonis* and *B. bifidum* (Figure 6L). These results showed that sunitinib could inhibit the growth of *P. distasonis* and *B. bifidum in vitro*. *P. distasonis* improved sunitinib-induced liver injury as shown by H&E staining and the decreased serum ALT level after *P. distasonis* treatment (Figure 8J-K).

### Hepatic FXR and gut microbiota participated in diurnal variation of sunitinib-induced liver injury

**Figure 9:**
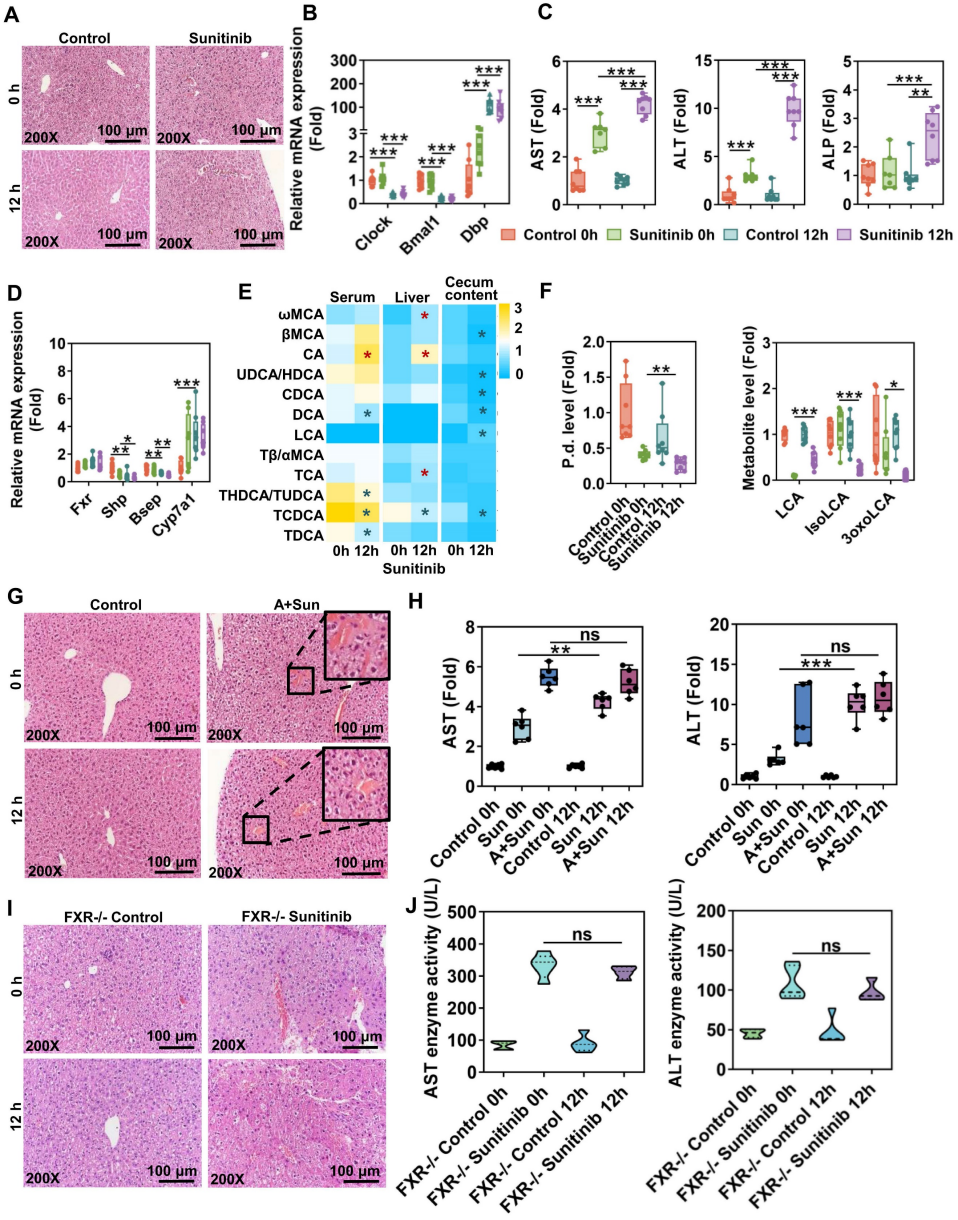
The diurnal variation of sunitinib-induced hepatotoxicity was associated with gut microbiota and FXR pathway. (**A**) H&E staining in mouse liver. (**B**) Clock gene expression in mouse liver. (**C**) Serum AST, ALT, and ALP levels. (**D**) FXR gene expression in mouse liver. (**E**) Bile acid level in mouse serum, liver and cecum content. (**F**) *P. distasonis* level in the cecum content of mice. LCA, isoLCA, and 3oxoLCA levels in cecum content of mice. (**G**) H&E staining of mouse liver. (**H**) Serum AST and ALT levels. (**I**) H&E staining of mouse liver. (**J**) Serum AST and ALT levels. *P < 0.05, **P < 0.01, ***P < 0.001.

Circadian rhythm plays an important role in DILI. Circadian timing can modify 2-10-fold the tolerability of anticancer medications in clinical cancer patients 50. Therefore, circadian rhythm of sunitinib, an anticancer medication, was evaluated. Sunitinib-induced mouse liver injury was enhanced at 12 h compared with 0 h as shown by H&E staining and increased serum AST, ALT, and ALP levels (Figure 9A, C), which was consistent with APAP-induced liver injury 51. Clock gene expression was different in liver sample between 0 h and 12 h. *Clock* and brain and muscle ARNT-like 1 (*Bmal1*) gene expression was decreased and *Dbp* gene expression was increased at 12 h (Figure 9B). FXR target genes *Shp* and *Bsep* were decreased and some bile acids were increased in serum and liver at 12 h (Figure 9D-E). Gut microbiota was influenced by diurnal variation: *P. distasonis* level was decreased and gut-residing bacteria produce isoLCA and 3oxoLCA were also decreased at 12 h (Figure 9F). The diurnal variation disappeared after antibiotic treatment or using FXR knockout mice (Figure 9G-J) indicating that gut microbiota and FXR pathway played an important role in diurnal variation.

## Discussion

Liver function impairment was found in 23-40% tumor patients after sunitinib treatment 1, 2. Sunitinib induced obvious liver injury and intestinal disorder in our study in mice. Spatial metabolomics, TEM, and immunofluorescence co-staining found that various hepatic cells (e.g., hepatocytes, bile duct cells, and LSECs) were impaired after sunitinib treatment (Figure 8L). FXR inhibition and gut microbiota depletion aggravated sunitinib-induced liver injury. Therefore, FXR activation and intestinal probiotics supplementation may be used to improve TKI-induced liver injury in clinical trials. The relationship between gut microbiota and TKI-induced side effect will be the future research directions.

The clinical dose of sunitinib was 50 mg/d-150 mg/d, which amounted to 6.5-19.5 mg/kg in mice, in renal cell carcinoma, gastrointestinal, neuroendocrine, and stromal tumors in clinical data, and the administration method was 4 weeks on and 2 weeks off 52. 40 mg/kg sunitinib (i.g., 4 days) improved malignant melanoma in female BALB/c *nu/nu* mice 53. 40 mg/kg sunitinib (1 week) improved breast cancer in C57BL/6 female mice 54. 120 mg/kg sunitinib (i.g., 14 days) induced liver injury in C57BL/6J mice 55. In our study, 75 mg/kg and 150 mg/kg sunitinib (i.g., 5 days) induced weak liver injury in C57BL/6J mice. As the impaired clearance of sunitinib in mouse liver with continuous administration 1, the sunitinib-induced hepatotoxicity may happen in therapeutic dose.

Liver injury is process achieved by the coordinated action of multiple cell types including hepatocytes, bile duct cells, LSECs, hepatic stellate cells, and Kupffer cells. Zonal liver injury could be observed in alcoholic- and non-alcoholic fatty liver diseases, drug-induced hepatotoxicity, or hepatocellular carcinoma. In our study, the concentration of sunitinib established gradients from hepatic portal vein to surrounding hepatocytes (Figure 1A), which spatially injured LSECs, bile duct cells, and hepatocytes around the hepatic portal vein. The hepatotoxicity of sunitinib in specific liver zonation was evaluated through H&E (bleeding), co-location of immunofluorescence, TEM for different cells, primary cell separation (hepatocytes, bile duct cells, and LSECs), spatial metabolome in our study. The zonated hepatotoxicity of *Mdr2-/-* mice was evaluated using co-location of immunofluorescence, spatial metabolome, intravital imaging after intravenous injection of cholyl-lysyl-fluorescein 38. Liver regeneration in specific liver zonation was evaluated using spatial transcriptomics and co-location of immunofluorescence 56.

At the single-cell level, spatial metabolomics was used to explore the spatial metabolic profile and tissue histology 57. Matrix-assisted laser desorption/ionization mass spectrometry imaging (MALDI-MSI) 38 and AFADESI-MSI 37, 58 were used to evaluate bile acid, amino acid, xanthine, carnitine, choline, MG, glucose, LPE, nucleic acid, and xenobiotic metabolite in mice liver or HepG2 spheroids. In our study, bile acid, lipid, acylcarnitine, choline, glucose, amino acid, nucleic acid, and sunitinib xenobiotic metabolites were observed after sunitinib treatment base on AFADESI-MSI.

LSECs are the first line of defense against toxins and metabolites 59, therefore we believe LSECs are the focus in sunitinib-induced liver injury. The loss of transcellular pores in LSEC injury has been reported in the previous study, which played a crucial role in the progression of liver injury 59. Of course, there are cross-talks between LSECs, bile duct cells and hepatocytes: (1) The metabolic function (e.g., iron homeostasis) and zonation of hepatocytes are regulated by LSECs through Wnt signaling and releasing bone morphogenetic protein 2/6 59. (2) The cross-talks between LSECs and bile duct cells have been poorly studied. LSECs can secrete the Notch signaling ligands, and the Notch signaling pathway is important for biliary repair 59. (3) Transdifferentiation between hepatocytes and bile duct cells has been recognized upon severe liver injury through Notch and Wnt signaling 60.

Autophagy was observed in carbon tetrachloride (CCl_4_)-, ischemia reperfusion-, or bile duct ligation (BDL)-induced liver injury, and concanavalin A-induced hepatitis 61. Various TKIs induced autophagy such as gefitinib, crizotinib, dasatinib, regorafenib, and sunitinib. Autophagy aggravated gefitinib- and crizotinib-induced liver injury 34, 62 and improved dasatinib- and regorafenib-induced liver injury 63, 64. Knocking down Beclin 1 to inhibit autophagy in H9c2 cells 65, using autophagy inhibitors 3-MA, bafilomycin A1, NH_4_Cl in H9c2 cells 33, and blocking autophagy using cardiomyocyte specific heterozygous autophagy-related protein 7 (*Atg7*)-deficient mice 66 improved sunitinib-induced cardiotoxicity. Sunitinib-induced autophagy has been observed in L02 cells (a hepatocyte) after 10 μM sunitinib treatment 67, but sunitinib-induced autophagy has not been observed in mouse liver in previous study. Sunitinib displayed a punctate pattern and was co-localized with LC3B protein in sunitinib-treated primary hepatocytes (Figure 1M), suggesting autophagy was closely related to the accumulation of sunitinib.

In our study, inhibiting autophagy using 3-MA and NH_4_Cl improved sunitinib-induced hepatic cell toxicity (Figure 5O-Q). FXR inhibited autophagy through binding to sites in autophagic gene promoter 45. Another research found that FXR-CREB regulated the hepatic autophagy 46. In our study, FXR was inhibited and autophagy was activated after sunitinib treatment in mice, and FXR was negative correlated with autophagy. Nearly all TKIs induced cell apoptosis. 400 mg/kg regorafenib induced apoptosis and mitochondrial dysfunction in liver as shown by H&E and TUNEL staining, and MitoTracker staining 43. Our study found that sunitinib also induced hepatocyte apoptosis and mitochondrial damage in mice which was consistent with previous study 68.

Circadian rhythm plays an important role in DILI through regulating multiple steps including drug transport, metabolism, diet, and molecular targets. Key clock genes *Clock* and *Bmal1* were decreased and *Dbp* was increased at 12 h in our study (Figure 9B) 69. Circadian timing can modify 2-10-fold the tolerability of anticancer medications in clinical patients 50. APAP-induced liver injury was increased at 12 h compared with 0 h 51. Gut microbiota *Saccharomyces cerevisiae* and bacterial metabolite (1-phenyl-1,2-propanedione) played an important role in modulating APAP-induced circadian rhythmic hepatotoxicity 51. CCl_4_ given in the morning produced no toxicity, and given in the evening resulted in elevation of toxicity in mice 70. Chloroform-induced hepatotoxicity was higher after the evening administration compared to the morning one in mice 71. Circadian rhythmic hepatotoxicity of *Tripterygium wilfordii* was observed in mice; with the highest level of hepatotoxicity at 2 h and the lowest at 14 h 72. The altered pharmacokinetics of triptolide participated in its toxicity 72. Morning administration of cis-platin was associated with worse nausea and vomiting than afternoon administration in clinical practice 73. Bile acid-related genes also displayed distinct circadian variations: *Shp* gene expression was decreased and *Cyp7a1* gene expression was increased at 12 h compared with 0 h (Figure 9D) 69. Our study found that gut microbiota and FXR pathway involved in circadian rhythmic hepatotoxicity induced by sunitinib. The difference in bedtime and feeding time between mice and human should be considered.

IsoLCA and 3oxoLCA were decreased in feces of submassive hepatic necrosis patients 74 and increased in centenarians 48. IsoLCA and 3oxoLCA could protect inflammatory arthritis 22, colitis 23, 75, and psoriasiform dermatitis 76 in previous studies. LCA improves *Klebsiella pneumonia*-induced liver abscess 77 and CCl_4_-induced inflammatory and liver fibrosis in mice 78. IsoLCA and 3oxoLCA exerted potent antimicrobial effects against gram-positive pathogens, including *Clostridioides difficile* and *Enterococcus feacium* 48. Another mechanism found that isoLCA and 3oxoLCA inhibited the differentiation of TH17 cells through inhibiting retinoic acid receptor-related orphan nuclear receptor-γt 23. Finally, 3oxoLCA inhibited the production of interleukin-17 (IL-17A) and blockade chemokine (C-C motif) ligand 20 (CCL20)-mediated trafficking 76. The relationship between 3oxoLCA and ileum FXR has not been evaluated. Our study found that 3oxoLCA protected sunitinib-induced liver injury through activating ileum FXR, inhibited bile acid alternative pathway (*Cyp27a1* and *Cyp7b1* gene expression), and finally decreased serum bile acid level. Ileum FGF15 protein was secreted into serum and inhibited hepatic bile acid synthesis alternative pathway (*Cyp7b1*) compared with bile acid synthesis classic pathway (*Cyp7a1*) 79. Activation of ileum FXR signaling using melatonin protected against aflatoxin B1-induced liver injury 80, and using the probiotic *Lactobacillus rhamnosus GG* treatment alleviated *Mdr2-/-* induced hepatic fibrosis 7. Therefore, activation of ileum FXR and inhibition of bile acid synthesis may improve sunitinib-induced liver injury.

*P. distasonis* improved hepatic fibrosis by increasing intestinal bile salt hydrolase (BSH) activity 5, improved non-alcoholic steatohepatitis through regulating metabolite pentadecanoic acid 81, improved metabolic dysfunctions through regulating secondary bile acid 82. *B. bifidum* protected against liver injury in animal and clinical experiments: *B. bifidum* improved non-alcoholic fatty liver in mice through regulating intestinal sterol biosynthesis 6; *B. bifidum* improved human alcohol-induced liver injury 83 and non-cirrhotic hepatitis C virus patient 84. Although 3αHSDH and 3βHSDH activities have not been reported in *B. bifidum*, the activities have been observed in *B. faecale*, *B. longum*, and *B. pseudocatenulaum* 23, 49. In our study, sunitinib decreased *B. bifidum* and *P. distasonis* probiotics *in vitro* (Figure 6I-L) and only *P. distasonis* could protect sunitinib-induced liver injury (Figure 8J-K).

The nuclear receptor FXR is a bile acid sensor that regulates bile acid homeostasis, lipid homeostasis and drug metabolism. FXR improved chronic and acute liver injury, such as cholestasis, alcoholic and non-alcoholic liver disease, and hepatic fibrosis 85. Various FXR agonists such as OCA have been development in clinical research to improve liver injury. FXR regulated bile acid synthesis, transport, and reabsorption process through hepatic SHP, hepatic BSEP and intestinal FGF19/15 19. In our study, metabolomics analysis found that bile acids were disordered in enterohepatic circulation (Figure 2I), so FXR pathway in sunitinib-induced liver injury was evaluated.

*Fxr^ΔL^* and *Fxr^ΔIE^* mice were used to demonstrate the role of hepatic and intestinal FXR in sunitinib-induced hepatotoxicity. In our study, the global FXR disruption is required for sunitinib-induced hepatotoxicity (Figure 5C-D). Both liver-specific and intestine-specific FXR disruption in mice resulted in a very low incidence of liver injury induced by sunitinib (Figure 5H-L). Previous study also observed the weak liver injury in tissue-specific Fxr-null mice compared with global FXR knockout mice: (1) The incidence of hepatic tumors was 90%, 20%, and 5% in 20-month-old global FXR knockout, *Fxr^ΔL^* and *Fxr^ΔIE^* mice, respectively 86. Serum AST and ALT activities and liver bile acids were increased in 20-month-old mice global FXR knockout but not in *Fxr^ΔL^* and *Fxr^ΔIE^* mice 86. (2) Serum bile acid levels were increased in global FXR knockout mice but not in *Fxr^ΔL^* and *Fxr^ΔIE^* mice; and TCA levels in bile acid pool (liver, gallbladder, and small intestinal) increased 2.6-, 1.4-, and 1.2-fold in global FXR knockout, *Fxr^ΔL^*, and *Fxr^ΔIE^* mice, respectively 21. Furthermore, FXR could also influence various non-gastrointestinal organs, such as immune system, central nervous system, kidney, cardiovascular system, and pancreatitis 87. Therefore, FXR in various organs may jointly induce the liver injury of sunitinib.

The limitations of the study included the following aspects: (1) Various TKIs including imatinib, erlotinib, sorafenib, sunitinib, pazopanib, anlotinib, and loratinib influenced lipid metabolism 9. Sunitinib influenced TG, TC, and decreased various lipid metabolites (e.g., LPC, LPE, MG, docosahexaenoic acid, and linoleic acid) (Figure 1D-E, Figure 3B), therefore lipidomics would be the further directions. (2) 16 bile acids in Figure 2I are not comprehensive as amino-conjugated bile acids 88, 3- succinylated bile acids 89, and 3-O-acyl-bile acids 90 are gradually discovered in recent years. The standards of these new bile acids are still noncommercial, therefore, these new bile acids would be the further directions. (3) Metagenomics analysis found that 1171 species were changed after sunitinib treatment (Figure 6G), and the function of other species apart from *P. distasonis* and *B. bifidum* would be the further directions.

## Conclusion

Sunitinib spatially injured LSECs, bile duct cells and hepatocytes around the hepatic portal vein because the concentration of sunitinib is heterogeneity in mouse liver. Inhibition of FXR-autophagy pathway and gut microbiota depletion aggravated sunitinib-induced liver injury. Circadian rhythmic hepatotoxicity induced by sunitinib was markedly enhanced at night compared with that at day, which was regulated by gut microbiota and FXR pathway.

## Supplementary Material

Supplementary figures and tables.

## Abbreviations

3oxoLCA: 3oxolithocholic acid
3-MA: 3-methyladenine
ALP: alkaline phosphatase
ALT: alanine aminotransferase
APAP: acetaminophen
AST: aspartate aminotransferase
*B. bifidum*: *Bifidobacterium bifidum*
CD31: platelet endothelial cell adhesion molecule 1
CDCA: chenodeoxycholic acid
FBS: fetal bovine serum
FXR: farnesoid X receptor
IsoLCA: isolithocholic acid
LC-MS: liquid chromatography- mass spectrometry
LSEC: liver sinusoids endothelial cell
*P. distasonis*: *Parabacteroides distasonis*
Sqstm1: sequestonsome1
TBA: total bile acid
TC: total cholesterol
TEM: transmission electron microscopy
TG: total triglycerides
TKI: tyrosine kinase inhibitor
TUNEL: terminal deoxynucleotidyl transferase dUTP nick end labeling
ZO-1: zonula occludens-1

## References

[1] Zhao Q, Zhang T, Xiao XR, Huang JF, Wang Y, Gonzalez FJ (2019). Impaired clearance of sunitinib leads to metabolic disorders and hepatotoxicity. Br J Pharmacol.

[2] Tang M, Wu ZE, Li F (2024). Integrating network pharmacology and drug side-effect data to explore mechanism of liver injury-induced by tyrosine kinase inhibitors. Comput Biol Med.

[3] Viganò M, La Milia M, Grassini MV, Pugliese N, Giorgio MD, Fagiuoli S (2023). Hepatotoxicity of small molecule protein kinase inhibitors for cancer. Cancers (Basel).

[4] Mueller EW, Rockey ML, Rashkin MC (2008). Sunitinib-related fulminant hepatic failure: case report and review of the literature. Pharmacotherapy.

[5] Zhao Q, Dai MY, Huang RY, Duan JY, Zhang T, Bao WM (2023). Parabacteroides distasonis ameliorates hepatic fibrosis potentially via modulating intestinal bile acid metabolism and hepatocyte pyroptosis in male mice. Nat Commun.

[6] Kim G, Yoon Y, Park JH, Park JW, Noh MG, Kim H (2022). Bifidobacterial carbohydrate/nucleoside metabolism enhances oxidative phosphorylation in white adipose tissue to protect against diet-induced obesity. Microbiome.

[7] Liu Y, Chen K, Li F, Gu Z, Liu Q, He L (2020). Probiotic Lactobacillus rhamnosus GG prevents liver fibrosis through inhibiting hepatic bile acid synthesis and enhancing bile acid excretion in mice. Hepatology.

[8] Grander C, Adolph TE, Wieser V, Lowe P, Wrzosek L, Gyongyosi B (2018). Recovery of ethanol-induced Akkermansia muciniphila depletion ameliorates alcoholic liver disease. Gut.

[9] Zhao Q, Wu ZE, Li B, Li F (2022). Recent advances in metabolism and toxicity of tyrosine kinase inhibitors. Pharmacol Ther.

[10] Su Z, Lu L, Chen F, Chen J, Chen X (2021). Gut microbiota and sunitinib-induced diarrhea in metastatic renal cell carcinoma: a pilot study. Cancer Manag Res.

[11] Yamamoto K, Kuzuya T, Honda T, Ito T, Ishizu Y, Nakamura M (2020). Relationship between adverse events and microbiomes in advanced hepatocellular carcinoma patients treated with sorafenib. Anticancer Res.

[12] Ervin SM, Hanley RP, Lim L, Walton WG, Pearce KH, Bhatt AP (2019). Targeting regorafenib-induced toxicity through inhibition of gut microbial β-glucuronidases. ACS Chem Biol.

[13] Inukai Y, Yamamoto K, Honda T, Ito T, Imai N, Ishizu Y (2023). Differences in the intestinal microbiome associated with diarrhea during lenvatinib treatment for hepatocellular carcinoma. Dig Dis.

[14] Wong CW, Yost SE, Lee JS, Gillece JD, Folkerts M, Reining L (2021). Analysis of gut microbiome using explainable machine learning predicts risk of diarrhea associated with tyrosine kinase inhibitor neratinib: a pilot study. Front Oncol.

[15] Ren P, Yue H, Tang Q, Wang Y, Xue C (2024). Astaxanthin slows down skeletal muscle atrophy in H22 tumor-bearing mice during sorafenib treatment by modulating the gut microbiota. Food Funct.

[16] Yan T, Yan N, Wang H, Yagai T, Luo Y, Takahashi S (2021). FXR-deoxycholic acid- TNF-α axis modulates acetaminophen-induced hepatotoxicity. Toxicol Sci.

[17] Huang W, Cao Z, Wang W, Yang Z, Jiao S, Chen Y (2024). Discovery of LH10, a novel fexaramine-based FXR agonist for the treatment of liver disease. Bioorg Chem.

[18] Jin J, Sun X, Zhao Z, Wang W, Qiu Y, Fu X (2015). Activation of the farnesoid X receptor attenuates triptolide-induced liver toxicity. Phytomedicine.

[19] Modica S, Petruzzelli M, Bellafante E, Murzilli S, Salvatore L, Celli N (2012). Selective activation of nuclear bile acid receptor FXR in the intestine protects mice against cholestasis. Gastroenterology.

[20] Sinal CJ, Tohkin M, Miyata M, Ward JM, Lambert G, Gonzalez FJ (2000). Targeted disruption of the nuclear receptor FXR/BAR impairs bile acid and lipid homeostasis. Cell.

[21] Kim I, Ahn SH, Inagaki T, Choi M, Ito S, Guo GL (2007). Differential regulation of bile acid homeostasis by the farnesoid X receptor in liver and intestine. J Lipid Res.

[22] Sun H, Guo Y, Wang H, Yin A, Hu J, Yuan T (2023). Gut commensal Parabacteroides distasonis alleviates inflammatory arthritis. Gut.

[23] Paik D, Yao L, Zhang Y, Bae S, D'Agostino GD, Zhang M (2022). Human gut bacteria produce Τ(Η)17-modulating bile acid metabolites. Nature.

[24] Wong JJW, Berstad MB, Fremstedal ASV, Berg K, Patzke S, Sørensen V (2020). Photochemically-induced release of lysosomal sequestered sunitinib: obstacles for therapeutic efficacy. Cancers (Basel).

[25] Phanthanawiboon S, Limkittikul K, Sakai Y, Takakura N, Saijo M, Kurosu T (2016). Acute systemic infection with dengue virus leads to vascular leakage and death through tumor necrosis factor-α and Tie2/angiopoietin signaling in mice lacking type I and II interferon receptors. PLoS One.

[26] Wu CY, Chan KC, Cheng YJ, Yeh YC, Chien CT (2015). Effects of different types of fluid resuscitation for hemorrhagic shock on splanchnic organ microcirculation and renal reactive oxygen species formation. Crit Care.

[27] Yao L, Li C, Ge X, Wang H, Xu K, Zhang A (2014). Establishment of a rat model of portal vein ligation combined with *in situ* splitting. PLoS One.

[28] Yang G, Jena PK, Hu Y, Sheng L, Chen SY, Slupsky CM (2023). The essential roles of FXR in diet and age influenced metabolic changes and liver disease development: a multi-omics study. Biomark Res.

[29] Katayanagi K, Kono N, Nakanuma Y (1998). Isolation, culture and characterization of biliary epithelial cells from different anatomical levels of the intrahepatic and extrahepatic biliary tree from a mouse. Liver.

[30] Chai C, Zheng S, Feng J, Wu X, Yang J, Wei M (2010). A novel method for establishment and characterization of extrahepatic bile duct epithelial cells from mice. In Vitro Cell Dev Biol Anim.

[31] Braet F, De Zanger R, Sasaoki T, Baekeland M, Janssens P, Smedsrød B (1994). Assessment of a method of isolation, purification, and cultivation of rat liver sinusoidal endothelial cells. Lab Invest.

[32] Maillard M, Arellano C, Vachoux C, Chevreau C, Cabaton NJ, Pont F (2022). Biological role of pazopanib and sunitinib aldehyde derivatives in drug-induced liver injury. Metabolites.

[33] Kimura T, Uesugi M, Takase K, Miyamoto N, Sawada K (2017). Hsp90 inhibitor geldanamycin attenuates the cytotoxicity of sunitinib in cardiomyocytes via inhibition of the autophagy pathway. Toxicol Appl Pharmacol.

[34] Luo P, Yan H, Du J, Chen X, Shao J, Zhang Y (2021). PLK1 (polo like kinase 1)- dependent autophagy facilitates gefitinib-induced hepatotoxicity by degrading COX6A1 (cytochrome c oxidase subunit 6A1). Autophagy.

[35] Yang J, Shi N, Wang S, Wang M, Huang Y, Wang Y (2024). Multi-dimensional metabolomic profiling reveals dysregulated ornithine metabolism hallmarks associated with a severe acute pancreatitis phenotype. Transl Res.

[36] Liu D, Huang J, Gao S, Jin H, He J (2022). A temporo-spatial pharmacometabolomics method to characterize pharmacokinetics and pharmacodynamics in the brain microregions by using ambient mass spectrometry imaging. Acta Pharm Sin B.

[37] Jiang HY, Gao HY, Li J, Zhou TY, Wang ST, Yang JB (2022). Integrated spatially resolved metabolomics and network toxicology to investigate the hepatotoxicity mechanisms of component D of Polygonum multiflorum Thunb. J Ethnopharmacol.

[38] Schneider KM, Candels LS, Hov JR, Myllys M, Hassan R, Schneider CV (2021). Gut microbiota depletion exacerbates cholestatic liver injury via loss of FXR signalling. Nat Metab.

[39] Zhou AP, Bai Y, Song Y, Luo H, Ren XB, Wang X (2019). Anlotinib versus sunitinib as first-line treatment for metastatic renal cell carcinoma: a randomized phase II clinical trial. Oncologist.

[40] Ni HM, McGill MR, Chao X, Du K, Williams JA, Xie Y (2016). Removal of acetaminophen protein adducts by autophagy protects against acetaminophen-induced liver injury in mice. J Hepatol.

[41] Wu S, Huang L, Shen R, Bernard-Cacciarella M, Zhou P, Hu C (2020). Drug resistance-related sunitinib sequestration in autophagolysosomes of endothelial cells. Int J Oncol.

[42] Giuliano S, Cormerais Y, Dufies M, Grépin R, Colosetti P, Belaid A (2015). Resistance to sunitinib in renal clear cell carcinoma results from sequestration in lysosomes and inhibition of the autophagic flux. Autophagy.

[43] Yan H, Wu W, Hu Y, Li J, Xu J, Chen X (2023). Regorafenib inhibits EphA2 phosphorylation and leads to liver damage via the ERK/MDM2/p53 axis. Nat Commun.

[44] Aparicio-Gallego G, Afonso-Afonso FJ, León-Mateos L, Fírvida-Pérez JL, Vázquez-Estévez S, Lázaro-Quintela M (2011). Molecular basis of hypertension side effects induced by sunitinib. Anticancer Drugs.

[45] Lee JM, Wagner M, Xiao R, Kim KH, Feng D, Lazar MA (2014). Nutrient-sensing nuclear receptors coordinate autophagy. Nature.

[46] Seok S, Fu T, Choi SE, Li Y, Zhu R, Kumar S (2014). Transcriptional regulation of autophagy by an FXR-CREB axis. Nature.

[47] Ellis JL, Bove KE, Schuetz EG, Leino D, Valencia CA, Schuetz JD (2018). Zebrafish abcb11b mutant reveals strategies to restore bile excretion impaired by bile salt export pump deficiency. Hepatology.

[48] Sato Y, Atarashi K, Plichta DR, Arai Y, Sasajima S, Kearney SM (2021). Novel bile acid biosynthetic pathways are enriched in the microbiome of centenarians. Nature.

[49] Xiao F, Dong F, Li X, Li Y, Yu G, Liu Z (2022). Bifidobacterium longum CECT 7894 improves the efficacy of infliximab for DSS-induced colitis via regulating the gut microbiota and bile acid metabolism. Front Pharmacol.

[50] Lévi F, Okyar A, Dulong S, Innominato PF, Clairambault J (2010). Circadian timing in cancer treatments. Annu Rev Pharmacol Toxicol.

[51] Gong S, Lan T, Zeng L, Luo H, Yang X, Li N (2018). Gut microbiota mediates diurnal variation of acetaminophen induced acute liver injury in mice. J Hepatol.

[52] Faivre S, Delbaldo C, Vera K, Robert C, Lozahic S, Lassau N (2006). Safety, pharmacokinetic, and antitumor activity of SU11248, a novel oral multitarget tyrosine kinase inhibitor, in patients with cancer. J Clin Oncol.

[53] Gaustad JV, Simonsen TG, Andersen LMK, Rofstad EK (2017). Antiangiogenic agents targeting different angiogenic pathways have opposite effects on tumor hypoxia in R-18 human melanoma xenografts. BMC Cancer.

[54] Argyros O, Karampelas T, Varela A, Asvos X, Papakyriakou A, Agalou A (2017). Targeting of the breast cancer microenvironment with a potent and linkable oxindole based antiangiogenic small molecule. Oncotarget.

[55] Guo L, Gong H, Tang TL, Zhang BK, Zhang LY, Yan M (2021). Crizotinib and sunitinib induce hepatotoxicity and mitochondrial apoptosis in L02 cells via ROS and Nrf2 signaling pathway. Front Pharmacol.

[56] Ben-Moshe S, Veg T, Manco R, Dan S, Papinutti D, Lifshitz A (2022). The spatiotemporal program of zonal liver regeneration following acute injury. Cell Stem Cell.

[57] Santos AA, Delgado TC, Marques V, Ramirez-Moncayo C, Alonso C, Vidal-Puig A (2024). Spatial metabolomics and its application in the liver. Hepatology.

[58] Li L, Zang Q, Li X, Zhu Y, Wen S, He J (2023). Spatiotemporal pharmacometabolomics based on ambient mass spectrometry imaging to evaluate the metabolism and hepatotoxicity of amiodarone in HepG2 spheroids. J Pharm Anal.

[59] Gao J, Lan T, Kostallari E, Guo Y, Lai E, Guillot A (2024). Angiocrine signaling in sinusoidal homeostasis and liver diseases. J Hepatol.

[60] Huang R, Zhang X, Gracia-Sancho J, Xie WF (2022). Liver regeneration: Cellular origin and molecular mechanisms. Liver Int.

[61] Zheng J, Chen L, Lu T, Zhang Y, Sui X, Li Y (2020). MSCs ameliorate hepatocellular apoptosis mediated by PINK1-dependent mitophagy in liver ischemia/reperfusion injury through AMPKα activation. Cell Death Dis.

[62] Li M, Wang C, Yu Z, Lan Q, Xu S, Ye Z (2022). MgIG exerts therapeutic effects on crizotinib-induced hepatotoxicity by limiting ROS-mediated autophagy and pyroptosis. J Cell Mol Med.

[63] Yang X, Wang J, Dai J, Shao J, Ma J, Chen C (2015). Autophagy protects against dasatinib-induced hepatotoxicity via p38 signaling. Oncotarget.

[64] Weng Z, Luo Y, Yang X, Greenhaw JJ, Li H, Xie L (2015). Regorafenib impairs mitochondrial functions, activates AMP-activated protein kinase, induces autophagy, and causes rat hepatocyte necrosis. Toxicology.

[65] Zhao Y, Xue T, Yang X, Zhu H, Ding X, Lou L (2010). Autophagy plays an important role in sunitinib-mediated cell death in H9c2 cardiac muscle cells. Toxicol Appl Pharmacol.

[66] Xu Z, Jin Y, Gao Z, Zeng Y, Du J, Yan H (2022). Autophagic degradation of CCN2 (cellular communication network factor 2) causes cardiotoxicity of sunitinib. Autophagy.

[67] Tang TL, Yang Y, Guo L, Xia S, Zhang B, Yan M (2022). Sunitinib induced hepatotoxicity in L02 cells via ROS-MAPKs signaling pathway. Front Pharmacol.

[68] Paech F, Abegg VF, Duthaler U, Terracciano L, Bouitbir J, Krähenbühl S (2018). Sunitinib induces hepatocyte mitochondrial damage and apoptosis in mice. Toxicology.

[69] Yang Y, Zhang J (2020). Bile acid metabolism and circadian rhythms. Am J Physiol Gastrointest Liver Physiol.

[70] Skrzypińska-Gawrysiak M, Piotrowski JK, Sporny S (2000). Circadian variations in hepatotoxicity of carbon tetrachloride in mice. Int J Occup Med Environ Health.

[71] Skrzypińska-Gawrysiak M, Piotrowski JK, Bruchajzer E (1995). The diurnal rhythm of hepatotoxic action of chloroform. Int J Occup Med Environ Health.

[72] Zhao H, Tong Y, Lu D, Wu B (2020). Circadian clock regulates hepatotoxicity of Tripterygium wilfordii through modulation of metabolism. J Pharm Pharmacol.

[73] Hofmeister EN, Fisher S, Palesh O, Innominato PF (2020). Does circadian rhythm influence gastrointestinal toxicity?. Curr Opin Support Palliat Care.

[74] Bao Z, Wei R, Zheng X, Zhang T, Bi Y, Shen S (2023). Landscapes of gut microbiome and bile acid signatures and their interaction in HBV-associated acute-on- chronic liver failure. Front Microbiol.

[75] Ma Y, Shan K, Huang Z, Zhao D, Zhang M, Ke W (2023). Bile acid derivatives effectively prevented high-fat diet-induced colonic barrier dysfunction. Mol Nutr Food Res.

[76] Shi Z, Wu X, Wu CY, Singh SP, Law T, Yamada D (2022). Bile acids improve psoriasiform dermatitis through inhibition of IL-17A expression and CCL20-CCR6- mediated trafficking of T cells. J Invest Dermatol.

[77] Zheng Y, Yue C, Zhang H, Chen H, Liu Y, Li J (2021). Deoxycholic acid and lithocholic acid alleviate liver injury and inflammation in mice with Klebsiella pneumoniae- induced liver abscess and bacteremia. J Inflamm Res.

[78] Shao J, Ge T, Tang C, Wang G, Pang L, Chen Z (2022). Synergistic anti-inflammatory effect of gut microbiota and lithocholic acid on liver fibrosis. Inflamm Res.

[79] Kuang J, Wang J, Li Y, Li M, Zhao M, Ge K (2023). Hyodeoxycholic acid alleviates non-alcoholic fatty liver disease through modulating the gut-liver axis. Cell Metab.

[80] Liu S, Kang W, Mao X, Ge L, Du H, Li J (2022). Melatonin mitigates aflatoxin B1-induced liver injury via modulation of gut microbiota/intestinal FXR/liver TLR4 signaling axis in mice. J Pineal Res.

[81] Wei W, Wong CC, Jia Z, Liu W, Liu C, Ji F (2023). Parabacteroides distasonis uses dietary inulin to suppress NASH via its metabolite pentadecanoic acid. Nat Microbiol.

[82] Wang K, Liao M, Zhou N, Bao L, Ma K, Zheng Z (2019). Parabacteroides distasonis alleviates obesity and metabolic dysfunctions via production of succinate and secondary bile acids. Cell Rep.

[83] Kirpich IA, Solovieva NV, Leikhter SN, Shidakova NA, Lebedeva OV, Sidorov PI (2008). Probiotics restore bowel flora and improve liver enzymes in human alcohol- induced liver injury: a pilot study. Alcohol.

[84] Ashour Z, Shahin R, Ali-Eldin Z, El-Shayeb M, El-Tayeb T, Bakr S (2022). Potential impact of gut Lactobacillus acidophilus and Bifidobacterium bifidum on hepatic histopathological changes in non-cirrhotic hepatitis C virus patients with different viral load. Gut Pathog.

[85] Rausch M, Samodelov SL, Visentin M, Kullak-Ublick GA (2022). The farnesoid X receptor as a master regulator of hepatotoxicity. Int J Mol Sci.

[86] Takahashi S, Tanaka N, Fukami T, Xie C, Yagai T, Kim D (2018). Role of farnesoid X receptor and bile acids in hepatic tumor development. Hepatol Commun.

[87] Yan N, Yan T, Xia Y, Hao H, Wang G, Gonzalez FJ (2021). The pathophysiological function of non-gastrointestinal farnesoid X receptor. Pharmacol Ther.

[88] Rimal B, Collins SL, Tanes CE, Rocha ER, Granda MA, Solanki S (2024). Bile salt hydrolase catalyses formation of amine-conjugated bile acids. Nature.

[89] Nie Q, Luo X, Wang K, Ding Y, Jia S, Zhao Q (2024). Gut symbionts alleviate MASH through a secondary bile acid biosynthetic pathway. Cell.

[90] Liu C, Du MX, Xie LS, Wang WZ, Chen BS, Yun CY (2024). Gut commensal Christensenella minuta modulates host metabolism via acylated secondary bile acids. Nat Microbiol.

