# Supplementary material for: Gut microbiota depletion and FXR inhibition exacerbates zonal hepatotoxicity of sunitinib
Source: Theranostics. 2024 Oct 28;14(18):7219–40. doi: 10.7150/thno.99926 (PMC11610149; doi:10.7150/thno.99926)
Supplement: Supplementary file 1 — Supplementary figures and tables. [file thnov14p7219s1.pdf]

Gut microbiota depletion and FXR inhibition exacerbates zonal hepatotoxicity of sunitinib

Qi Zhao[1,2*], Yingmei Lu[1*], Jingyi Duan[1], Dan Du[3], Qianlun Pu[3], Fei Li[1,2,4]

[1]Department of Gastroenterology & Hepatology, Laboratory of Hepatointestinal Diseases and Metabolism, Frontiers Science Center for Disease-related Molecular Network, West China Hospital, Sichuan University, Chengdu, 610041, China

[2]Huaxi Joint Centre for Gastrointestinal Cancer, West China Hospital, Sichuan University, Chengdu, 610041, China

[3]Advanced Mass Spectrometry Center, Research Core Facility, Frontiers Science Center for Disease-related Molecular Network, West China Hospital, Sichuan University, Chengdu, 610041, China

[4]Huaxi Joint Centre for Gastrointestinal Cancer, State Key Laboratory of Respiratory Health and Multimorbidity, West China Hospital, Sichuan University, Chengdu, 610041, China

Figures

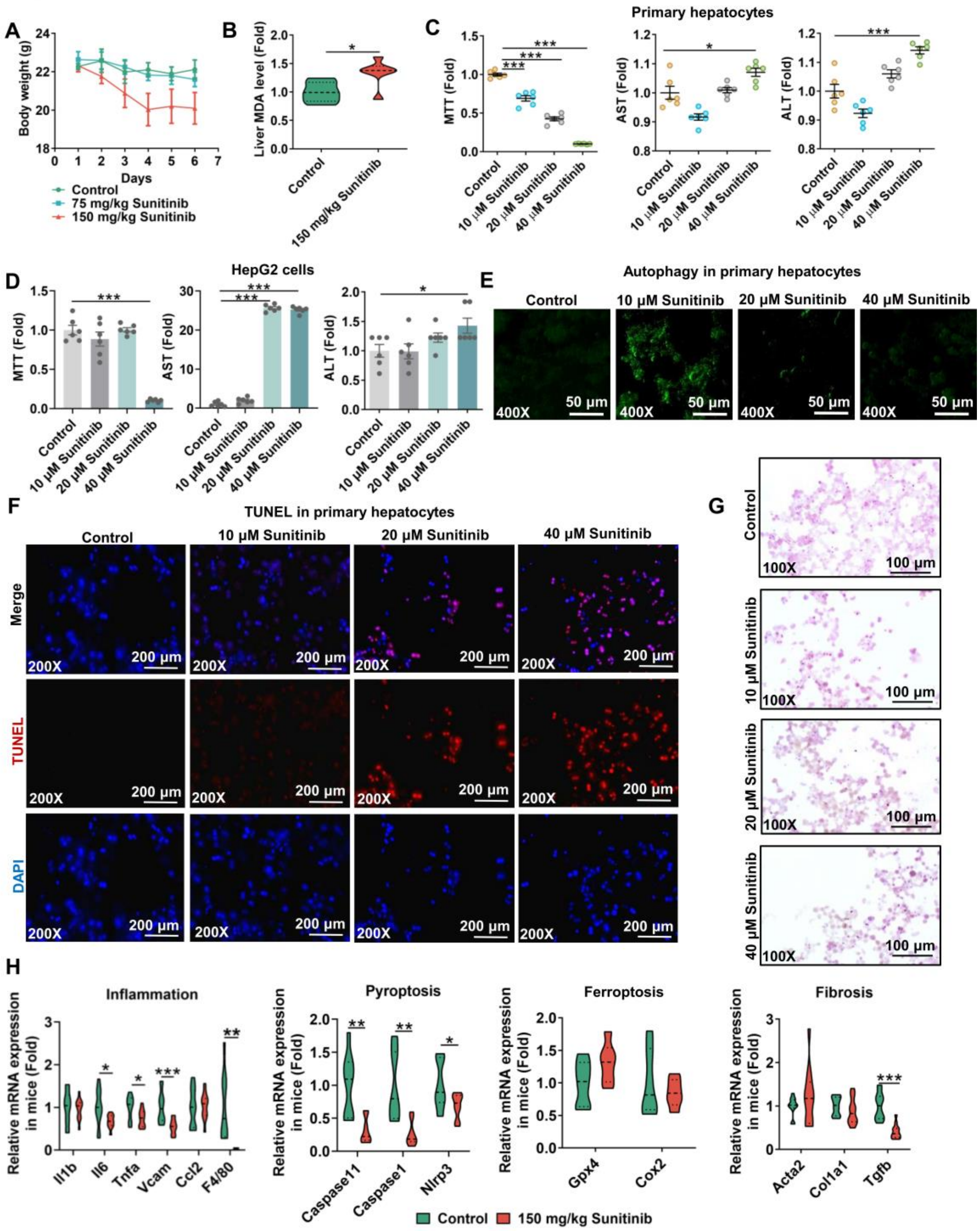

Figure S1. Hepatotoxicity of sunitinib in mice and cells. (**A**) Body weight. (**B**) Liver MDA level. (**C**) Cell viability, AST and ALT levels in primary hepatocytes. (**D**) Cell viability, AST and ALT levels in HepG2 cells. (**E**) Autophagy level in primary hepatocytes using cell autophagy detection assay kit. (**F**) TUNEL staining for primary hepatocytes. (**G**) Immunohistochemical analysis found that Caspase-3 protein expression was increased in mouse primary hepatocytes. (**H**) Inflammatory factors, pyroptosis, ferroptosis, and hepatic fibrosis gene expressions in mouse liver. **P* < 0.05, ***P* < 0.01, ****P* < 0.001.

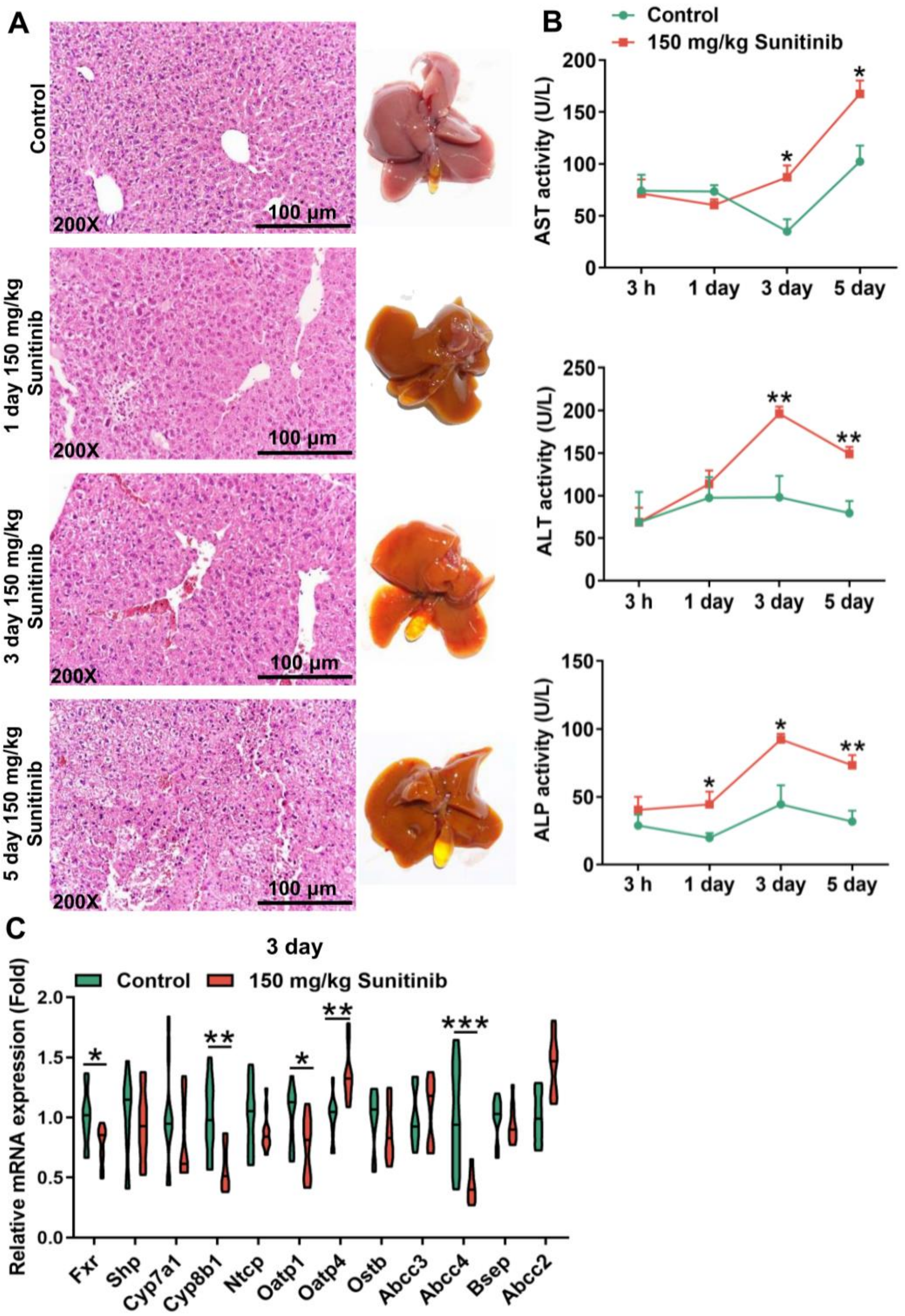

Figure S2. Time-dependent effect of sunitinib-induced liver injury. (**A**) H&E staining and representative image of liver. (**B**) Serum AST, ALT, and ALP levels. (**C**) FXR- and bile acid-related gene expressions after 3 day sunitinib treatment. *P < 0.05, **P < 0.01, ***P < 0.001.

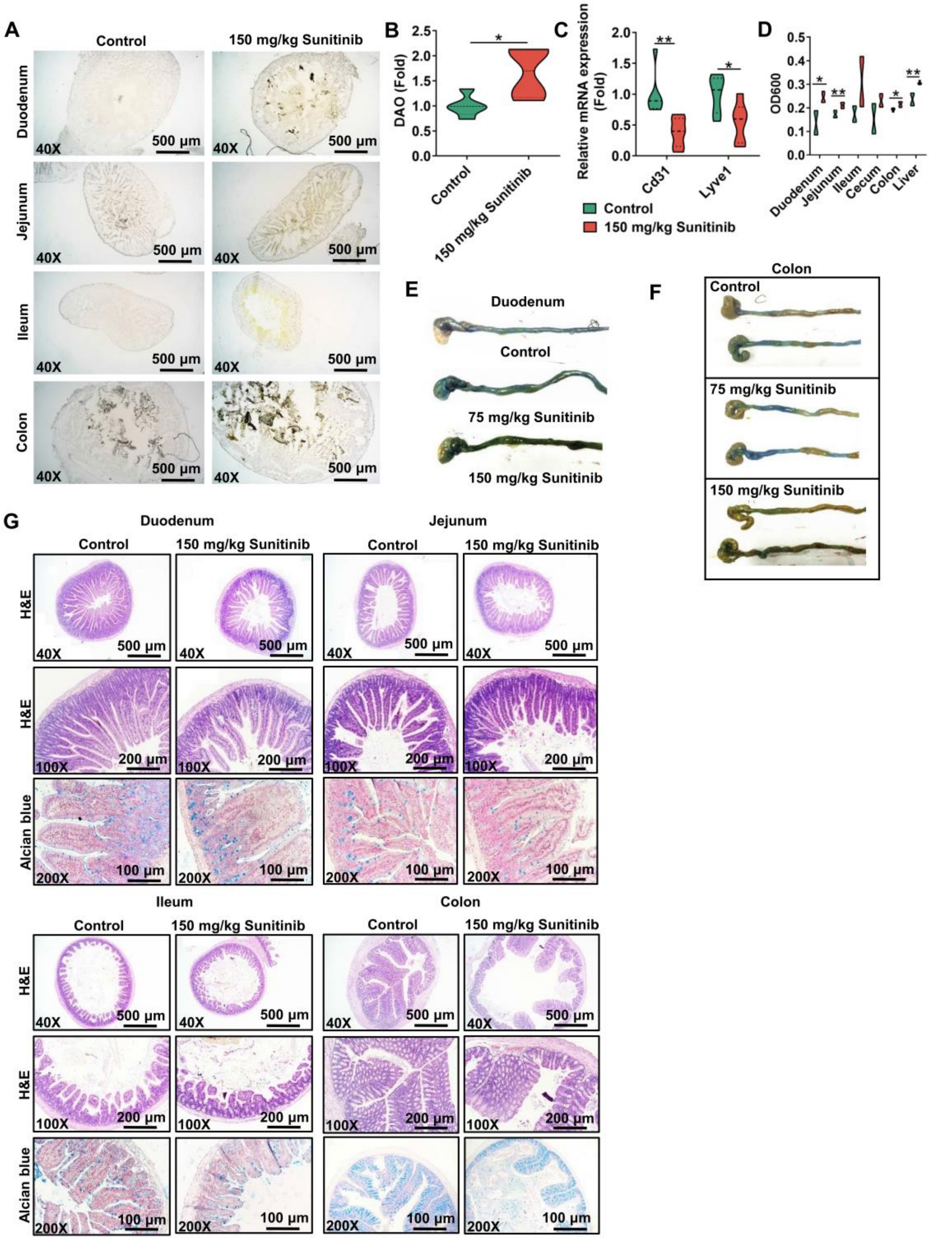

Figure S3. Weak intestinal injury of sunitinib was observed in mice. (**A**) Drug distribution of sunitinib in duodenum, jejunum, ileum, and colon. (**B**) DAO level in mouse serum. (**C**) Vascular injury gene expression in ileum tissue. (**D**) Evans blue staining, method to evaluate bleeding, was quantified by OD600 value. (**E-F**) Evans blue staining for duodenum and colon. (**G**) H&E and Alcian blue staining, method to evaluate goblet cell and vascular permeability, in mouse duodenum, jejunum, ileum, and colon. **P* < 0.05, ***P* < 0.01.

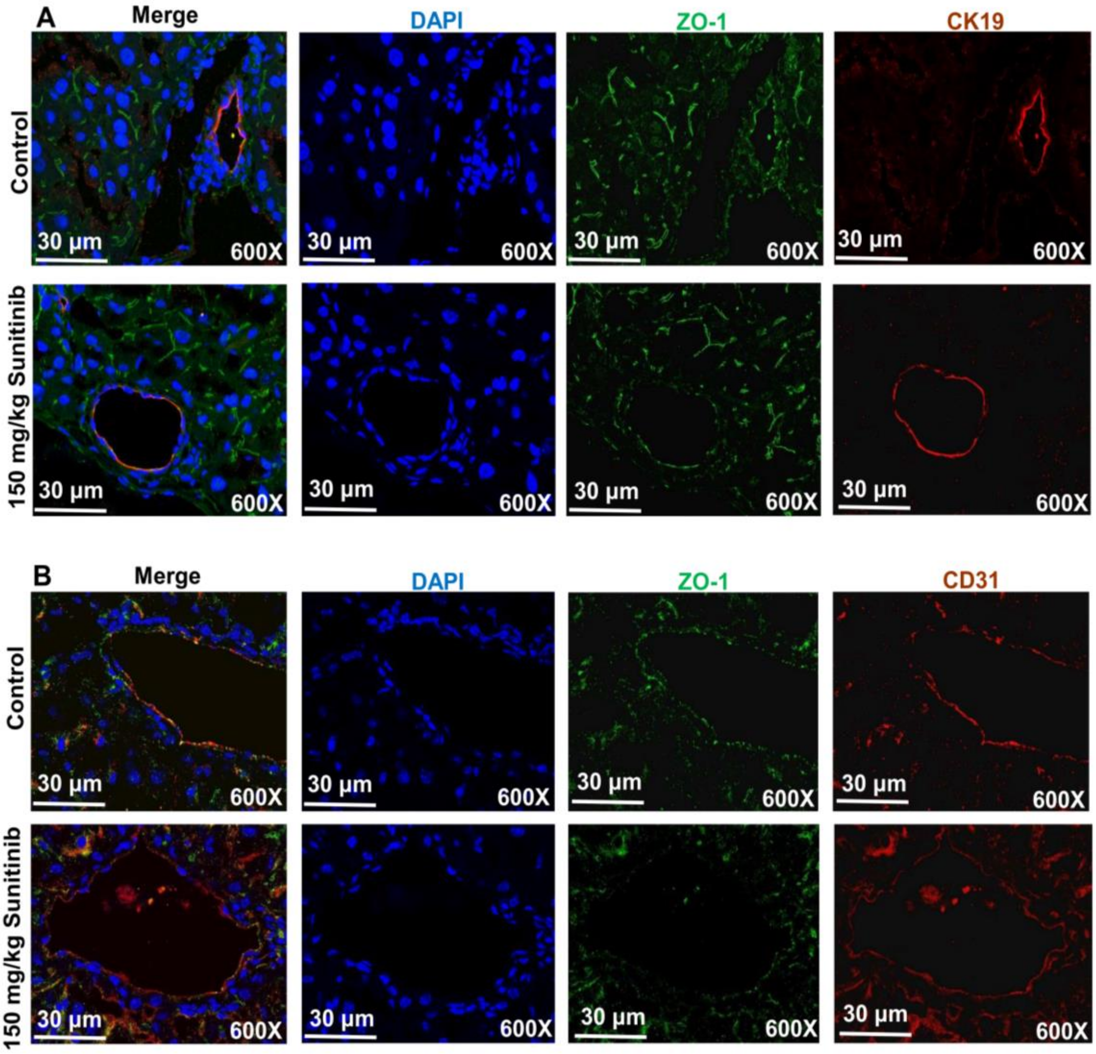

Figure S4. Co-staining of CK19 and ZO-1 (**A**), CD31 and ZO-1 (**B**) in mouse liver. The fluorescence of CK19 and CD31 was decreased because sunitinib impaired bile duct cells and LSECs, respectively.

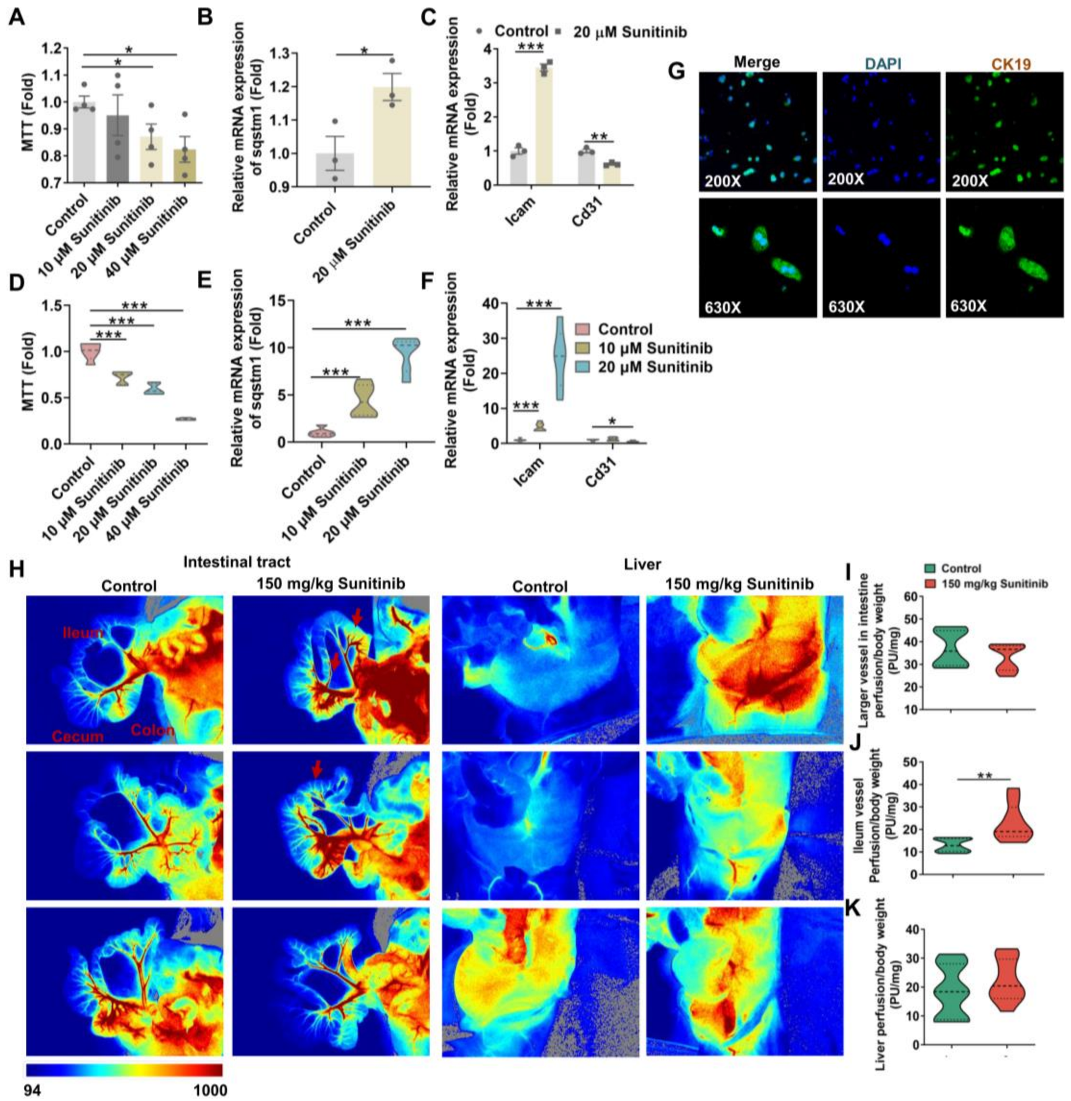

Figure S5. Bile duct cell and LSEC injury after sunitinib treatment. (**A**) Cell viability in mouse primary LSEC cells. (**B**) Autophagy gene expression in mouse primary LSEC cells. (**C**) Vascular injury gene expression in mouse primary LSEC cells. (**D**) Cell viability in HUVECs. (**E**) Autophagy gene expression in HUVECs. (**F**) Vascular injury gene expression in HUVECs. (**G**) Staining of CK19 in mouse primary bile duct cells. (**H**) Blood flow volume evaluated by Laser speckle contrast imaging in intestinal tract (left including ileum, cecum, and colon) and liver tissues (right). (**I**) Quantify of blood flow volume in large vessel of intestinal by Laser speckle contrast imaging. (**J**) Quantify of blood flow volume in ileum vessel by Laser speckle contrast imaging (arrow in Fig. S5H). (**K**) Quantify of blood flow volume in liver by Laser speckle contrast imaging. *P < 0.05, **P < 0.01, ***P < 0.001.

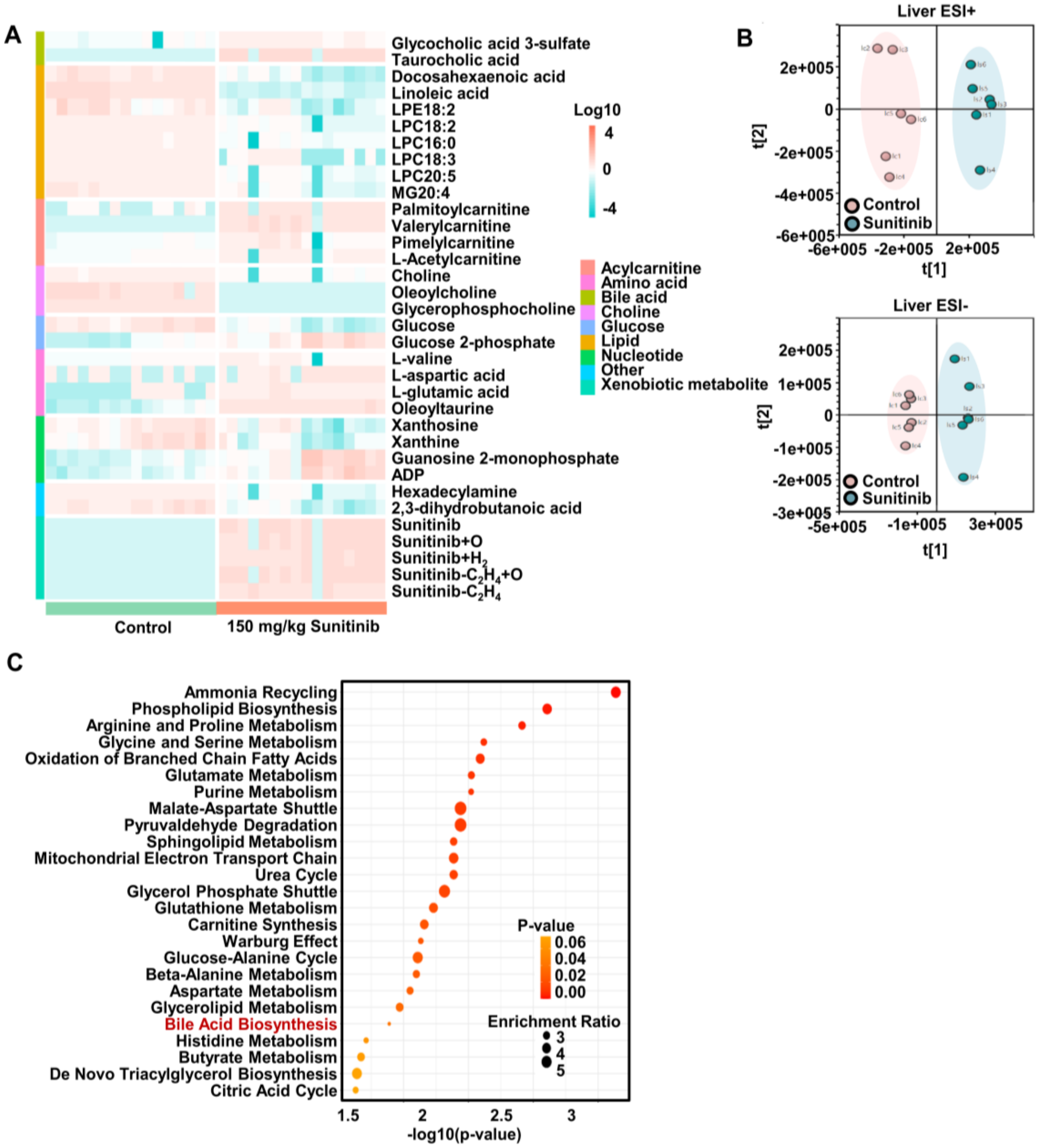

Figure S6. Changed metabolite and pathway in mouse liver using spatial metabolomics and non-target metabolomics. (**A**) Metabolite level in mouse liver using spatial metabolomics. This was the quantify of Figure 3. (**B**) Using metabolomics, PCA score plot derived from UPLC-MS data in liver ions in ESI+ (up) and ESI- (down) modes. Each point represented an individual mouse liver sample. (**C**) Using metabolomics, hepatic pathway analysis was carried out using MetaboAnalyst 4.0 and bile acid pathway was influenced after sunitinib treatment.

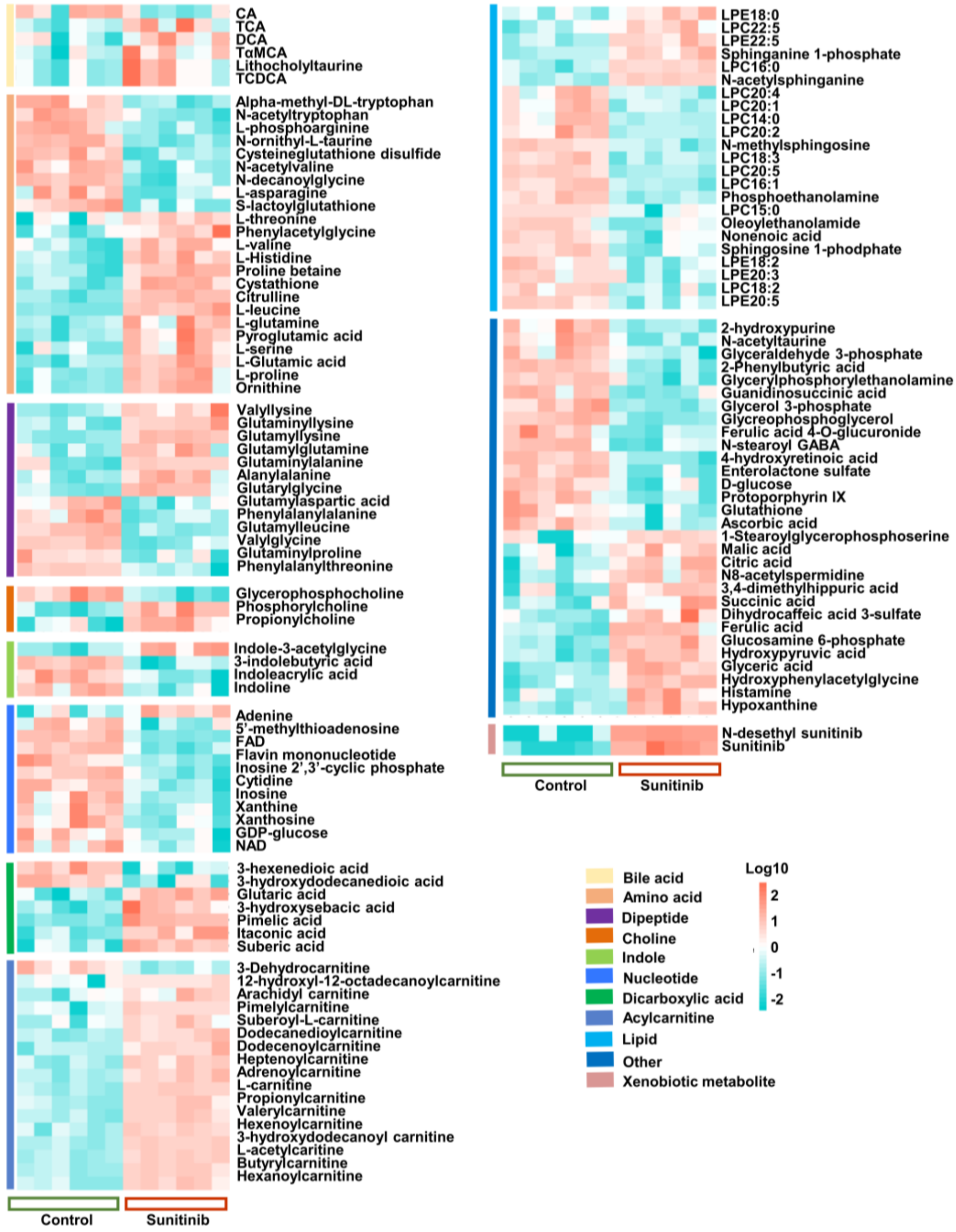

Figure S7. Metabolite level in mouse liver using non-target metabolomics.

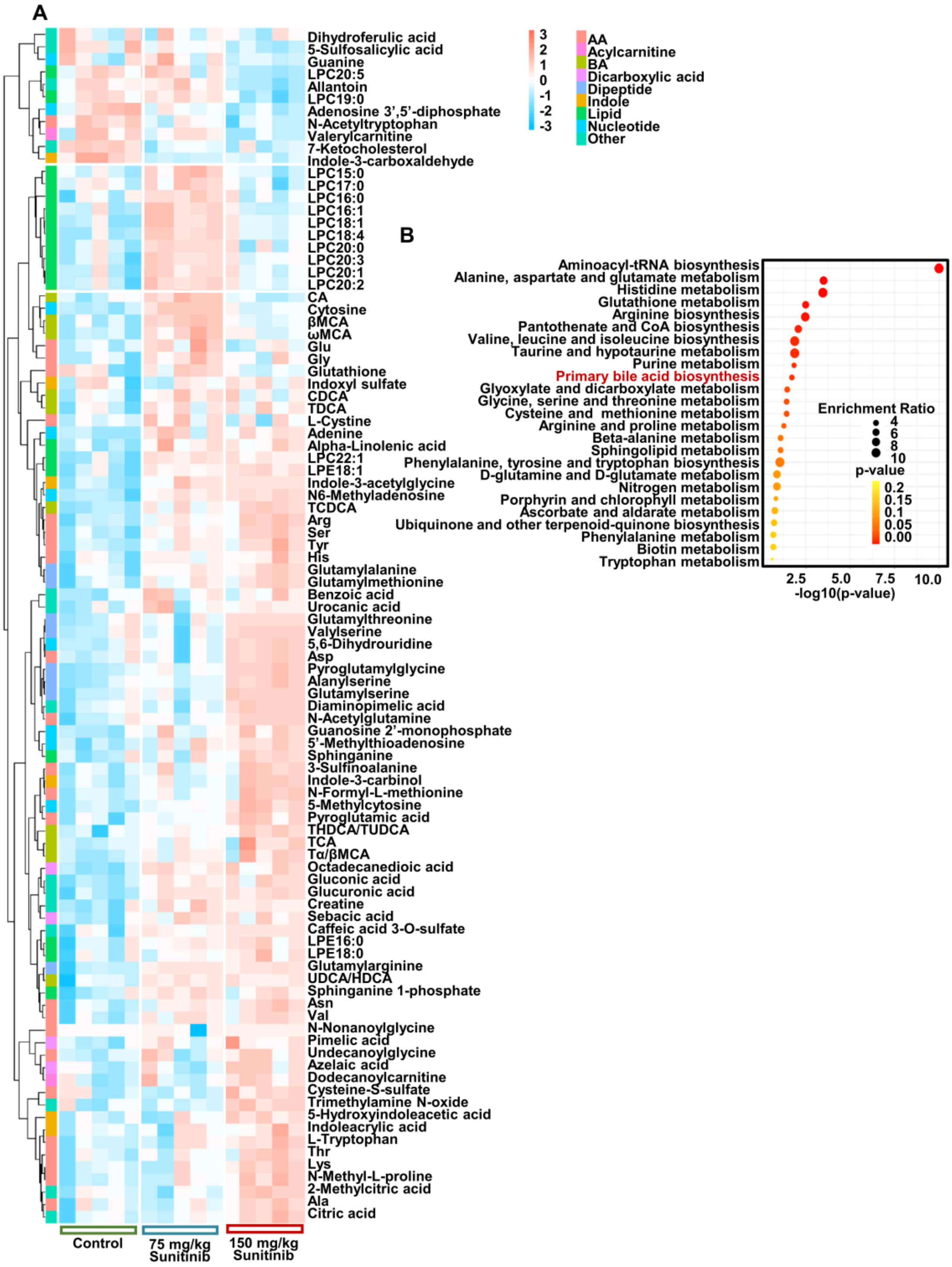

Figure S8. The changed metabolites (**A**) and pathway (**B**) in serum using non-target metabolomics analysis. Pathway analysis was carried out using MetaboAnalyst 4.0.

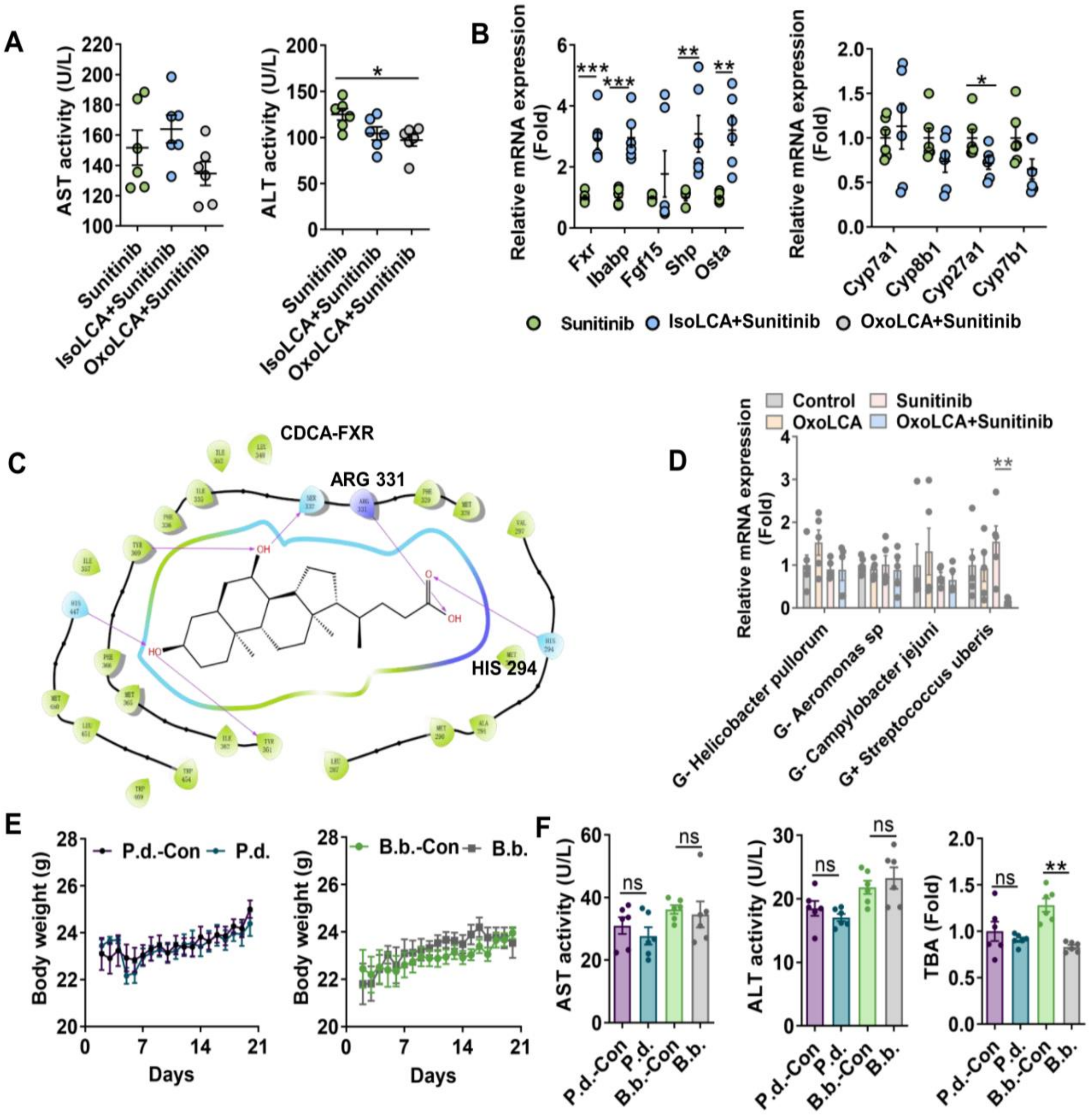

Figure S9. 3oxoLCA inhibited pathogens. (**A**) Serum AST and ALT levels after isoLCA and 3oxoLCA treatment. (**B**) Ileum FXR and hepatic bile acid synthesis gene expression after isoLCA and 3oxoLCA treatment. (**C**) Docking pose of CDCA into the human FXR ligand-binding pocket. (**D**) 3oxoLCA inhibited gram positive pathogen *Streptococcus uberis*. (**E**) Body weight after *P. distasonis* and *B. bifidum* treatment in mice. (**F**) Serum AST, ALT, and TBA levels after *P. distasonis* and *B. bifidum* treatment in mice. $^{*}P < 0.05$, $^{**}P < 0.01$, $^{***}P < 0.001$.

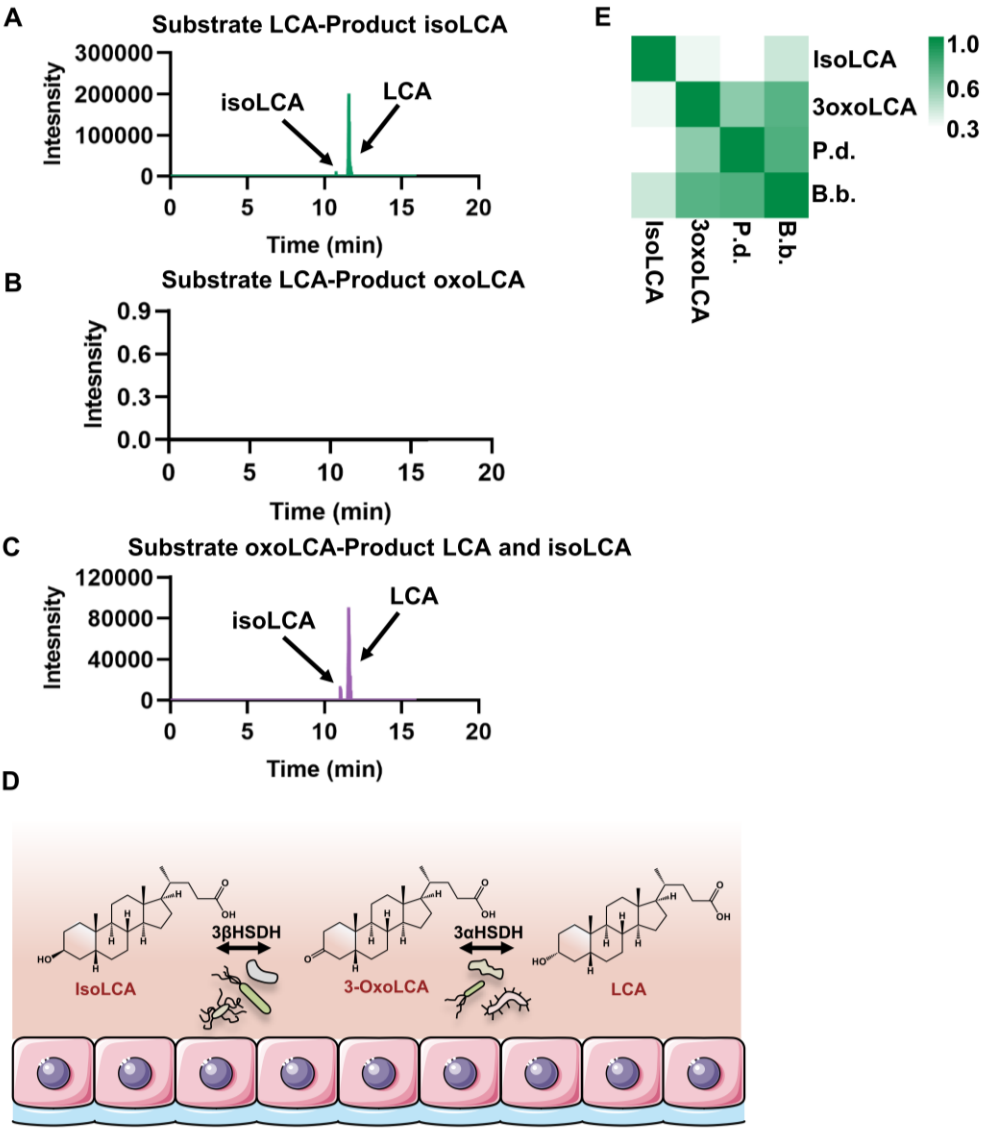

Figure S10. *P. distasonis* produced isoLCA and 3oxoLCA *in vitro*. (**A**) *P. distasonis* exposed to LCA produced isoLCA *in vitro*. (**B**) *P. distasonis* exposed to LCA not produced 3oxoLCA *in vitro*. (**C**) *P. distasonis* exposed to 3oxoLCA produced LCA and isoLCA *in vitro*. (**D**) 3αHSDH and 3βHSDH activity in gut microbiota. (**E**) Correlation analysis between isoLCA or 3oxoLCA and gut microbiota.

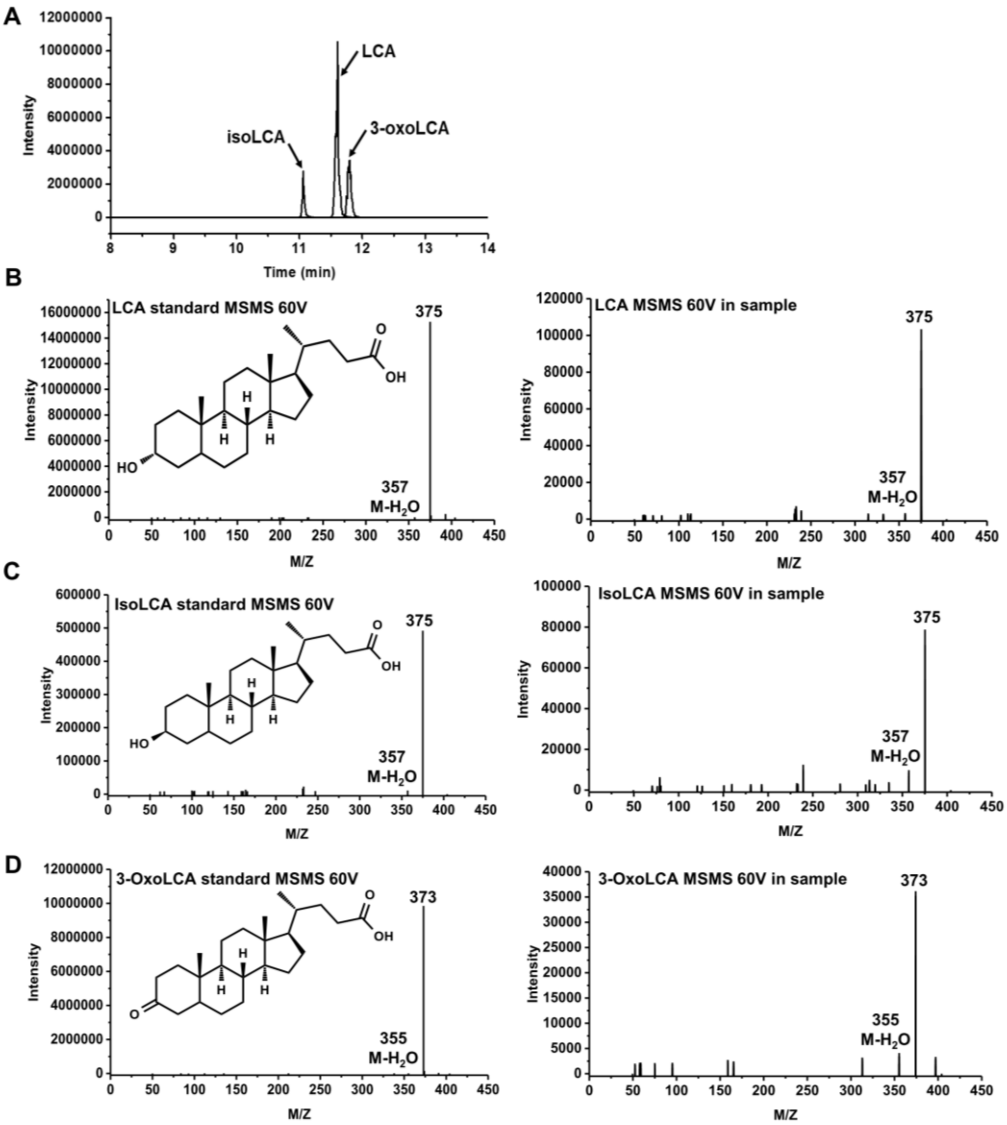

Figure S11. MSMS of LCA, isoLCA, and 3oxoLCA.

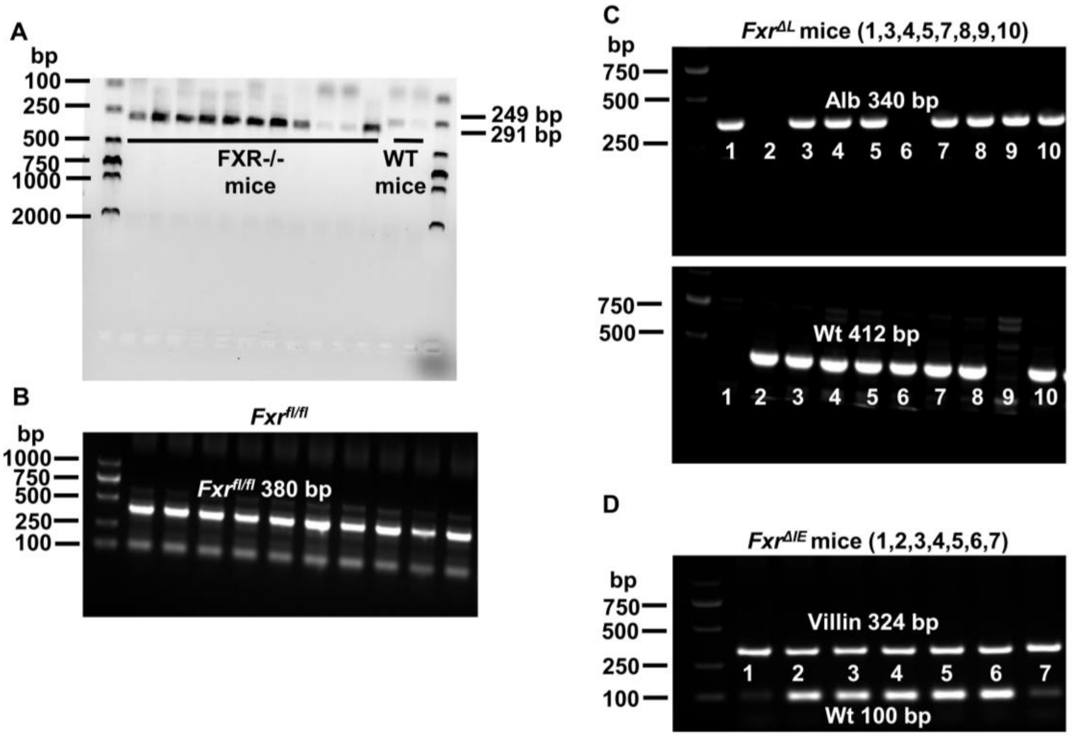

Figure S12. (**A**) PCR genotyping of conventional FXR knockout mice (249 bp for wild-type mice, 291 bp for conventional FXR knockout mice). (**B**) PCR genotyping of $Fxr^{fl/fl}$ mice (300 bp for wild-type mice, 380 bp for $Fxr^{fl/fl}$ mice). (**C**) Alb-Cre mice were used for the generation of $Fxr^{\Delta L}$ mice. 1, 3, 4, 5, 7, 8, 9,10 mice were $Fxr^{\Delta L}$ mice, and 2 and 6 mice were $Fxr^{fl/fl}$ mice (412 bp for wild-type mice, 340 bp for Alb-Cre mice). (**D**) Villin-Cre mice were used for the generation of $Fxr^{\Delta IE}$ mice. 1-7 mice were $Fxr^{\Delta IE}$ mice (100 bp for wild-type mice, 324 bp for Villin-Cre mice).

Table S1. Sunitinib influenced 34 mouse liver metabolites using spatial metabolomics analysis.

No.	Compound	Formula	m/z
1	Taurocholic acid	C26H45NO7S	516.2950 M+H
2	Oleoylcholine	C23H46NO2	368.3504 M+H
3	Choline	C5H14NO	104.1065 M+H
4	Glycerophosphocholine	C8H20NO6P	258.1088 M+H
5	Acetylcarnitine	C9H17NO4	204.1220 M+H
6	Hexadecylamine	C16H35N	242.2830 M+H
7	Valerylcarnitine	C12H23NO4	246.1740 M+H
8	Pimelylcarnitine	C14H25NO6	304.1739 M+H
9	Palmitoylcarnitine	C23H45NO4	400.3397 M+H
10	L-Valine	C5H11NO2	118.0857 M+H
11	LPC16:0	C24H50NO7P	496.3371 M+H
12	LPC18:3	C26H48NO7P	518.3186 M+H
13	LPC18:2	C26H50NO7P	520.3372 M+H
14	LPC20:5	C28H48NO7P	542.3190 M+H
15	MG20:4	C23H38O4	379.2796 M+H
16	Sunitinib+O	C22H27FN4O3	415.2117 M+H
17	Sunitinib+H_2	C22H29FN4O2	401.2234 M+H
18	Sunitinib	C22H27FN4O2	399.2169 M+H
19	Sunitinib-C_2H_4+O	C20H23FN4O3	387.1805 M+H
20	Sunitinib-C_2H_4	C20H23FN4O2	371.1858 M+H
21	Docosahexaenoicacid(DHA)	C22H32O2	327.2330 M-H
22	alpha-Linoleic acid	C18H30O2	277.2172 M-H
23	2,3-Dihydroxybutanoic acid	C4H8O4	119.0353 M-H
24	L-Aspartic acid	C4H7NO4	132.0304 M-H
25	L-Glutamic acid	C5H9NO4	146.0476 M-H
26	LPE18:2	C23H44NO7P	476.2781 M-H
27	Oleoyltaurine	C20H39NO4S	388.2526 M-H
28	Xanthosine	C10H12N4O6	283.0683 M-H
29	Guanosine 2-monophosphate	C10H14N5O8P	362.0505 M-H
30	Xanthine	C5H4N4O2	151.0267 M-H
31	ADP	C10H15N5O10P2	426.0217 M-H
32	Glycocholic acid 3-sulfate	C26H43NO9S	544.2658 M-H
33	D-Glucose	C6H12O6	179.0564 M-H
34	D-Glucose 2-phosphate	C6H13O9P	259.0218 M-H

Table S2. Sunitinib influenced 140 mouse liver metabolites using non-target metabolomics analysis.

No.	Compound	Mass M+H/M-H	Mode	Rt(min)	MSMS	Formula
1	N-Ornithyl-L-taurine	240.1012	ESI+	10.42	86/125	C7H17N3O4S
2	LPC20:5	542.3241	ESI+	10.23	86/104/184	C28H48NO7P
3	N-Decanoylglycine	230.1751	ESI+	10.83	127/184	C12H23NO3
4	4-Hydroxyretinoic acid	317.2111	ESI+	10.39	271/299	C20H28O3
5	2-Phenylbutyric acid	165.0910	ESI+	12.31	77/91/119	C10H12O2
6	FAD	786.1644	ESI+	4.53	136/348/439	C27H33N9O15P2
7	3-Indolebutyric acid	204.1019	ESI+	5.98	168/186	C12H13NO2
8	Glutamylleucine	261.1445	ESI+	1.04	114/215	C11H20N2O5
9	LPC15:0	482.3241	ESI+	10.55	86/104/184	C23H48NO7P
10	Valylglycine	175.1077	ESI+	0.95	59/74/100/115	C7H14N2O3
11	LPC16:1	494.3241	ESI+	9.83	86/104/184	C24H48NO7P
12	n-Methylsphingosine	328.2846	ESI+	13.18	88/283	C19H37NO3
13	LPC18:3	518.3241	ESI+	11.71	86/104/184	C26H48NO7P
14	LPE20:5	500.2772	ESI+	10.16	359/439	C25H42NO7P
15	Sphingosine 1-phosphate	396.2873	ESI+	11.39	81/298/378	C19H42NO5P
16	alpha-Methyl-DL-tryptophan	219.1128	ESI+	4.66	102/173/202	C12H14N2O2
17	Cytosine	112.0505	ESI+	0.83	95/67	C4H5N3O
18	N-Stearoyl GABA	370.3316	ESI+	12.54	87/268/352	C22H43NO3
19	Indoleacrylic acid	188.0706	ESI+	5.45	89/115/142/170	C11H9NO2
20	Glycerol 3-phosphate	173.0210	ESI+	0.82	75/87/89/101	C3H9O6P
21	L-Phosphoarginine	255.0853	ESI+	12.06	81/95/131/159/173	C6H15N4O5P
22	Nonenoic acid	157.1223	ESI+	10.71	58/98/111/139	C9H16O2
23	Cysteineglutathione disulfide	427.0952	ESI+	0.88	85/122/130/296/352	C13H22N4O8S2
24	N-Acetylvaline	160.0968	ESI+	4.08	58/101/114	C7H13NO3
25	S-Lactoylglutathione	380.1122	ESI+	1.47	84/130/148/162	C13H21N3O8S
26	3-Hexenedioic acid	145.0495	ESI+	0.84	58/71/99/104	C6H8O4
27	Guanidinosuccinic acid	176.0666	ESI+	0.86	60/89/112/117/130/158	C5H9N3O4
28	Indoline	120.0808	ESI+	5.37	103	C8H9N
29	LPE18:2	478.2928	ESI+	10.16	337/460	C23H44NO7P
30	Protoporphyrin IX	563.2653	ESI+	13.14	431/445/504	C34H34N4O4
31	LPC18:2	520.3397	ESI+	10.56	86/104/184	C26H50NO7P
32	LPC14:0	468.3084	ESI+	9.48	86/104/184	C22H46NO7P
33	2-Hydroxypurine	137.0458	ESI+	1.04	116	C5H4N4O
34	Glycerophosphocholine	258.1101	ESI+	0.95	60/86/104/125/184	C8H20NO6P
35	LPC20:2	548.3710	ESI+	11.3	86/104/184	C28H54NO7P
36	Ferulic acid 4-O-glucuronide	371.0973	ESI+	13.79	132/177/325	C16H18O10
37	L-Asparagine	133.0608	ESI+	0.80	74/88/116	C4H8N2O3
38	LPC20:1	550.3867	ESI+	12.17	86/104/184	C28H56NO7P
39	Oleoylethanolamide	326.3053	ESI+	12.74	62/308	C20H39NO2
40	Glutamylaspartic acid	263.0874	ESI+	0.93	102/117/130/132/217	C9H14N2O7
41	Glutaminylproline	244.1292	ESI+	0.92	114/130/141/198/199	C10H17N3O4
42	Xanthine	153.0407	ESI+	1.05	56/81/107/135	C5H4N4O2
43	5'-Methylthioadenosine	298.0968	ESI+	3.04	136/162	C11H15N5O3S
44	LPC20:4	544.3397	ESI+	10.96	86/104/184	C28H50NO7P
45	Glutamylglutamine	276.1190	ESI+	0.97	85/130/230	C10H17N3O6
46	Glutaminyllysine	275.1714	ESI+	0.93	99/130/144	C11H22N4O4
47	Arachidyl carnitine	456.4047	ESI+	11.98	295/397	C27H53NO4
48	1-Stearoylglycerophosphoserine	526.3139	ESI+	12.18	88/184/258/341	C24H48NO9P
49	Dodecenoylcarnitine	342.2639	ESI+	8.22	144/283	C19H35NO4
50	Adenine	136.0618	ESI+	0.91	67/82/92/94/109/119	C5H5N5
51	LPC22:5	570.3554	ESI+	10.81	86/104/184	C30H52NO7P
52	LPE18:0	482.3241	ESI+	11.80	341/421/464	C23H48NO7P
53	Suberoyl-L-carnitine	318.1911	ESI+	4.70	144/157/259	C15H27NO6
54	Sunitinib	399.2191	ESI+	6.12	238/255/283/326	C22H27FN4O2
55	L-Glutamine	147.0764	ESI+	0.81	84/101/130	C5H10N2O3
56	Hypoxanthine	137.0458	ESI+	6.65	94/110/119	C5H4N4O
57	Alanylalanine	161.0921	ESI+	0.91	72/88/115/116	C6H12N2O3
58	12-Hydroxy-12-octadecanoylcarnitine	444.3683	ESI+	10.51	144/385	C25H49NO5
59	Dodecanedioylcarnitine	374.2537	ESI+	6.33	144/213/315	C19H35NO6
60	N8-Acetylspermidine	188.1757	ESI+	0.77	71/131/143/170	C9H21N3O
61	Indole-3-acetylglycine	233.0921	ESI+	5.10	102/130	C12H12N2O3
62	Pimelylcarnitine	304.1755	ESI+	3.07	143/144/245	C14H25NO6
63	Sphinganine 1-phosphate	382.2717	ESI+	9.61	266/284/364	C18H40NO5P
64	Ornithine	133.0971	ESI+	0.71	70/116	C5H12N2O2
65	L-Valine	118.0863	ESI+	0.84	55/57/59/72	C5H11NO2
66	L-Proline	116.0706	ESI+	0.71	70	C5H9NO2
67	Valyllysine	246.1812	ESI+	0.78	72/100/130	C11H23N3O3
68	Pyroglutamic acid	130.0499	ESI+	0.81	56/84	C5H7NO3
69	L-Histidine	156.0767	ESI+	0.78	56/68/83/93/110	C6H9N3O2
70	L-Glutamic acid	148.0604	ESI+	0.83	84/102/131	C5H9NO4
71	Hexanoylcarnitine	260.1856	ESI+	5.20	99/144/201	C13H25NO4
72	Propionylcarnitine	218.1387	ESI+	1.04	57/144/159	C10H19NO4
73	Adrenoylcarnitine	476.3734	ESI+	10.82	144/417	C29H49NO4
74	Proline betaine	144.1019	ESI+	0.93	55/98	C7H13NO2
75	LPC16:0	480.3448	ESI+	11.12	86/104/184	C24H50NO6P
76	Hexenoylcarnitine	258.1700	ESI+	4.78	97/144/199	C13H23NO4
77	Butyrylcarnitine	232.1543	ESI+	3.42	71/144/173	C11H21NO4
78	Valerylcarnitine	246.1700	ESI+	4.14	85/144/187	C12H23NO4
79	N-Desethyl sunitinib	371.1878	ESI+	5.26	238/255/283/326	C20H23FN4O2
80	Heptenoylcarnitine	272.1856	ESI+	5.32	144/213	C14H25NO4
81	N-Acetylsphinganine	344.3159	ESI+	10.00	58/102/132/326	C20H41NO3
82	L-Leucine	132.1019	ESI+	0.91	86	C6H13NO2
83	Glutamyllysine	276.1554	ESI+	0.84	102/145/230	C11H21N3O5
84	Citrulline	176.1030	ESI+	0.98	70/114/117/159	C6H13N3O3
85	L-Carnitine	162.1125	ESI+	0.84	60/85/102/103	C7H15NO3
86	L-Acetylcarnitine	204.1230	ESI+	1.04	57/60/85/145	C9H17NO4
87	3-Hydroxydodecanoyl carnitine	360.2744	ESI+	7.03	144/301	C19H37NO5
88	Phenylalanylthreonine	263.1037	ESI-	5.45	77/118/163	C13H16N2O4
89	Glycerophosphoglycerol	245.0432	ESI-	0.82	155/171/229	C6H15O8P
90	Flavin mononucleotide	455.0973	ESI-	4.88	79/97	C17H21N4O9P
91	Glycerylphosphorylethanolamine	214.0486	ESI-	0.79	60/140/155/171	C5H14NO6P
92	Cytidine	242.0782	ESI-	0.82	153/182/198	C9H13N3O5
93	Enterolactone sulfate	377.0700	ESI-	6.34	97/107/189/297	C18H18O7S

94	Phosphoethanolamine	140.0118	ESI-	0.78	79	C2H8NO4P
95	N-Acetyltryptophan	245.0932	ESI-	5.46	58/74/98/116/203	C13H14N2O3
96	Ascorbic acid	175.0248	ESI-	1.03	56/69/87/113	C6H8O6
97	3-Dehydrocarnitine	158.0823	ESI-	4.16	100/115	C7H13NO3
98	3-Hydroxydodecanedioic acid	245.1394	ESI-	6.57	139/184/201	C12H22O5
99	N-Acetyltaurine	166.0180	ESI-	0.96	58/80/124	C4H9NO4S
100	Inosine	267.0735	ESI-	1.05	103/135/231	C10H12N4O5
101	D-Glucose	179.0561	ESI-	0.85	59/71/89/113	C6H12O6
102	Glyceraldehyde 3-phosphate	168.9907	ESI-	0.82	79/97/151	C3H7O6P
103	Phenylalanylalanine	235.1088	ESI-	3.26	88/148/163	C12H16N2O3
104	Xanthosine	283.0684	ESI-	2.71	108/151	C10H12N4O6
105	Inosine 2',3'-cyclic phosphate	329.0293	ESI-	3.67	135/178	C10H11N4O7P
106	Glutathione	306.0765	ESI-	1.05	59/128/143/179/254/272	C10H17N3O6S
107	GDP-glucose	604.0699	ESI-	0.91	150/442	C16H25N5O16P2
108	LPE20:3	502.2939	ESI-	10.6	154/305	C25H46NO7P
109	NAD	662.1018	ESI-	0.97	159/211/272/328/346/408/426	C21H27N7O14P2
110	Succinic acid	117.0193	ESI-	0.84	73/99	C4H6O4
111	L-Threonine	118.0510	ESI-	0.83	56/72/74	C4H9NO3
112	Histamine	110.0724	ESI-	0.78	66/80/81	C5H9N3
113	Dihydrocaffeic acid 3-sulfate	261.0074	ESI-	3.67	81/136/181	C9H10O7S
114	3,4-Dimethylhippuric acid	206.0823	ESI-	5.53	59/102/162	C11H13NO3
115	L-Serine	104.0353	ESI-	0.78	72/74	C3H7NO3
116	3-Hydroxysebacic acid	217.1081	ESI-	3.80	111/155/173	C10H18O5
117	Malic acid	133.0142	ESI-	0.84	59/71/73/89/115	C4H6O5
118	Citric acid	191.0197	ESI-	0.88	57/85/87/111	C6H8O7
119	Propionylcholine	159.1265	ESI-	6.81	57/73/103	C8H18NO2
120	Phenylacetylglycine	192.0666	ESI-	5.08	74/91	C10H11NO3
121	Glucosamine 6-phosphate	258.0384	ESI-	0.90	79/97	C6H14NO8P
122	Glutaminylalanine	216.0990	ESI-	1.51	73/101/117/127/143/200	C8H15N3O4
123	Phosphorylcholine	183.0666	ESI-	0.88	79/81/86	C5H15NO4P
124	LPE22:5	526.2939	ESI-	10.75	140/387/467	C27H46NO7P
125	Itaconic acid	129.0193	ESI-	0.82	73/83/85/111	C5H6O4
126	Glutarylglycine	188.0564	ESI-	1.05	87/102/115/144	C7H11NO5
127	Suberic acid	173.0819	ESI-	3.62	111/155	C8H14O4
128	Ferulic acid	193.0506	ESI-	4.16	58/136/149	C10H10O4
129	Glutaric acid	131.0350	ESI-	2.68	69/87/113	C5H8O4
130	Cystathione	221.0601	ESI-	0.79	102/120/134	C7H14N2O4S
131	Hydroxyphenylacetylglycine	208.0615	ESI-	4.98	59/102/135	C10H11NO4
132	Pimelic acid	159.0663	ESI-	3.62	54/97/115/141	C7H12O4
133	Glyceric acid	105.0193	ESI-	0.91	59/75	C3H6O4
134	Hydroxypyruvic acid	103.0037	ESI-	0.83	57/59/72/75	C3H4O4
135	Cholic acid	407.2803	ESI-	8.58	289/325/343/389	C24H40O5
136	Deoxycholic acid	391.2854	ESI-	10.15	327/345/355/373	C24H40O4
137	Tauro-alpha-muricholic acid	514.2844	ESI-	6.31	79/124	C26H45NO7S
138	Taurocholic acid	514.2844	ESI-	7.09	79/124	C26H45NO7S
139	Taurochenodesoxycholic acid	498.2895	ESI-	7.95	79/124	C26H45NO6S
140	Lithocholyltaurine	482.2946	ESI-	9.53	79/124	C26H45NO5S

Table S3. Sunitinib influenced 126 mouse cecum content metabolites using non-target metabolomics analysis.

No.	Compound	Rt(min)	Mass M+H/M-H	Mode	Formula	MSMS
1	Putrescine	0.66	89.1073	ESI+	C4H12N2	55/72
2	1,3-Diaminopropane	0.69	75.0917	ESI+	C3H10N2	58/62
3	Spermidine	0.64	146.1652	ESI+	C7H19N3	58/72/84/112
4	Aminomethanesulfonic acid	0.65	112.0063	ESI+	CH5NO3S	94/95
5	Taurine	0.77	126.0219	ESI+	C2H7NO3S	65/80/108
6	N-Palmitoyl lysine	7.80	385.3425	ESI+	C22H44N2O3	350/367
7	Cyclopentenyl cytosine	4.79	240.0979	ESI+	C10H13N3O4	110/222
8	Glutamylleucine	1.02	261.1445	ESI+	C11H20N2O5	84/86/130/148
9	4-Pyridoxic acid	2.31	184.0604	ESI+	C8H9NO4	148/166
10	alpha-Linolenic acid	12.7	279.2318	ESI+	C18H30O2	67/81/95/109/123/137
11	N-Stearoyl lysine	8.69	413.3737	ESI+	C24H48N2O3	145/367/395
12	5'-Methylthioadenosine	3.50	298.0968	ESI+	C11H15N5O3S	61/75/97/136
13	Xanthine	1.02	153.0407	ESI+	C5H4N4O2	55/82/92/107/110/136
14	Tyrosine methylester	1.02	196.0968	ESI+	C10H13NO3	119/136/179
15	Dihydroxyphenylacetic acid sulfate	6.04	249.0063	ESI+	C8H8O7S	95/231
16	Indole-3-carboxylic acid	4.60	162.0550	ESI+	C9H7NO2	64/89/116/117
17	Methyl-3H-thymidine	1.01	257.1132	ESI+	C11H16N2O5	125/239
18	Prolylproline	1.02	213.1234	ESI+	C10H16N2O3	70/116
19	N-Hydroxy-L-tyrosine	1.01	198.0761	ESI+	C9H11NO4	136/154/180
20	Bilirubin	7.34	585.2707	ESI+	C33H36N4O6	225/299
21	Acetylcholine	0.85	146.1175	ESI+	C7H15NO2	60/87
22	7Z,10Z-Hexadecadienoic acid	7.70	253.2162	ESI+	C16H28O2	209/235
23	Monoketocholic acid	6.40	423.2741	ESI+	C24H38O6	351/369/387
24	Hydroxyoctadecadienoic acid	7.62	297.2424	ESI+	C18H32O3	235/279
25	Indoleacetaldehyde	2.58	160.0757	ESI+	C10H9NO	91/115/117/118/132
26	Indole-3-propionic acid	6.77	190.0863	ESI+	C11H11NO2	55/130
27	N1,N8-Diacetylspermidine	1.02	230.1863	ESI+	C11H23N3O2	55/70/100/114/143
28	Arginyltyrosine	0.76	338.1823	ESI+	C15H23N5O4	60/70/95/136/147/158/175
29	Argininosuccinic acid	0.78	291.1299	ESI+	C10H18N4O6	116/133/158/176/273
30	Indolelactic acid	5.45	206.0812	ESI+	C11H11NO3	130/188
31	3-Methylhistamine	0.66	126.1026	ESI+	C6H11N3	68/96/109
32	5Z-Dodecenoic acid	11.90	199.1692	ESI+	C12H22O2	55/69/83/181
33	Myristoylglycine	5.63	286.2377	ESI+	C16H31NO3	226/268
34	Glutamine-glutamate	0.87	292.1139	ESI+	C10H17N3O7	84/102/131/148
35	Asparagine glutamate	0.79	278.0983	ESI+	C9H15N3O7	74/84/88/102/130/148
36	Protoporphyrin IX	13.23	563.2653	ESI+	C34H34N4O4	401/415/430/445/459/504
37	Oleoylethanolamide	12.81	326.3053	ESI+	C20H39NO2	62/83/95/309
38	Flavin mononucleotide	3.68	457.1119	ESI+	C17H21N4O9P	117/243/359/439
39	gamma-Glutamyllysine	3.32	276.1554	ESI+	C11H21N3O5	84/102/129
40	N-Acetylhistidine	0.90	198.0873	ESI+	C8H11N3O3	83/95/110/152/156
41	Pantothenic acid	1.05	220.1179	ESI+	C9H17NO5	72/90/98/124/142
42	Stearoylethanolamide	13.71	328.3210	ESI+	C20H41NO2	71/267/311
43	Valylthreonine	3.23	219.1339	ESI+	C9H18N2O4	60/86/87/132
44	N-Methylphenylalanine	6.77	180.1019	ESI+	C10H13NO2	119/134/162
44	1-Methylnicotinamide	0.79	137.0709	ESI+	C7H8N2O	67/92/94
46	Alanylproline	0.87	187.1077	ESI+	C8H14N2O3	70/72/116
47	LPE16:1	9.76	452.2772	ESI+	C21H42NO7P	311/391/434
48	Leu-Glu	0.79	277.1394	ESI+	C11H20N2O6	84/86/130/132/148
49	Agmatine	0.68	131.1291	ESI+	C5H14N4	72/114
50	Sphingosine	12.48	300.2897	ESI+	C18H37NO2	57/71/85/95/264/283
51	Methyl 5-hydroxyoxindole-3-acetate	4.46	222.0761	ESI+	C11H11NO4	58/130/148/163/204
52	3-Dehydrocarnitine	3.07	160.0968	ESI+	C7H13NO3	83/101
53	Dodecanedioic acid	4.79	229.1490	ESI-	C12H22O4	57/139/167/211
54	Cysteinyl-Proline	5.95	217.0697	ESI-	C8H14N2O3S	100/200
55	5-Thymidylic acid	1.52	321.0538	ESI-	C10H15N2O8P	78/96/125/176/195
56	Tyramine-O-sulfate	3.76	216.0381	ESI-	C8H11NO4S	119/137/200
57	Riboflavin cyclic-4',5'-phosphate	3.98	437.0913	ESI-	C17H19N4O8P	180/255/402
58	N-Acetyltaurine	0.94	166.0225	ESI-	C4H9NO4S	58/80/94/108/124
59	4-Methoxycinnamic acid	4.47	177.0602	ESI-	C10H10O3	103/133
60	Dihydroxybenzylamine	1.02	138.0606	ESI-	C7H9NO2	91/102/120
61	Xanthurenic acid 8-O-sulfate	4.84	283.9916	ESI-	C10H7NO7S	80/96/204
62	12-Hydroxydodecanoic acid	9.60	215.1698	ESI-	C12H24O3	59/144/167
63	Mesobilirubinogen	8.87	591.3233	ESI-	C33H44N4O6	255/290/422/466
64	7-Ketodeoxycholic acid	7.91	405.2691	ESI-	C24H38O5	369/387
65	Decanoylcarnitine	10.42	314.2382	ESI-	C17H33NO4	74/171
66	Trihydroxystearic acid	7.78	331.2535	ESI-	C18H36O5	277/295/313
67	Norepinephrine sulfate	1.02	248.0279	ESI-	C8H11NO6S	80/168
68	Phthalamic acid	5.33	164.0398	ESI-	C8H7NO3	76/103/120
69	Tetradecanedioic acid	8.73	257.1803	ESI-	C14H26O4	195/239
70	Udecanoylcarnitine	11.19	328.2538	ESI-	C18H35NO4	74/116/310
71	3-Sulfodeoxycholic acid	7.26	457.2311	ESI-	C23H38O7S	80/97
72	3-Indoleglyoxylic acid	5.31	188.0398	ESI-	C10H7NO3	144
73	Taurocholic acid 3-sulfate	6.10	594.2457	ESI-	C26H45NO10S2	80/498/514
74	Dihydrocaffeic acid 3-sulfate	3.32	261.0120	ESI-	C9H10O7S	80/97/148
75	Glutaric acid	2.67	131.0395	ESI-	C5H8O4	69/87
76	Azelaic acid	5.80	187.1021	ESI-	C9H16O4	57/97/123/125/169

77	Benzyl alcohol	4.56	107.0548	ESI-	C7H8O	65/79
78	Uric acid	1.01	167.0256	ESI-	C5H4N4O3	69/96/124
79	Cysteic acid	0.78	168.0017	ESI-	C3H7NO5S	72/80/86
80	Suberic acid	5.05	173.0864	ESI-	C8H14O4	57/81/83/109/111/154
81	Sebacic acid	6.48	201.1177	ESI-	C10H18O4	57/111/137/139/183
82	Indole-3-carboxaldehyde	4.67	144.0500	ESI-	C9H7NO	116/126
83	N-Acetylaspartylglutamic acid	1.02	303.0879	ESI-	C11H16N2O8	96/128/226
84	Uridine	1.50	243.0668	ESI-	C9H12N2O6	110/140/151/200
85	Indole-3-carboxylic acid-O-sulphate	4.04	240.0017	ESI-	C9H7NO5S	80/160
86	Epinephrine sulfate	2.74	262.0436	ESI-	C9H13NO6S	80/182/189
87	Inosine	1.02	267.0780	ESI-	C10H12N4O5	135
88	3-Hydroxydodecanedioic acid	5.80	245.1440	ESI-	C12H22O5	183/201/227
89	N-Acetylthreonine	1.02	160.0660	ESI-	C6H11NO4	74/98/116
90	3-Hydroxysuberic acid	4.92	189.0814	ESI-	C8H14O5	99/127/129
91	LPE14:0	9.41	424.2515	ESI-	C19H40NO7P	140/214/227
92	Stercobilin	8.82	593.3390	ESI-	C33H46N4O6	257/291/424/468
93	L-Phenylalanine	0.83	164.0762	ESI-	C9H11NO2	72/119/147
94	Oleoyl glycine	12.77	338.2746	ESI-	C20H37NO3	59/74
95	Ascorbic acid	1.01	175.0293	ESI-	C6H8O6	69/85/87/113
96	Glucose 6-phosphate	0.78	259.0270	ESI-	C6H13O9P	79/97/139/169/199
97	Isocoproporphyrin	8.07	653.2662	ESI-	C36H38N4O8	521/565/609
98	Leucyl-Serine	3.23	217.1239	ESI-	C9H18N2O4	87/103/116/130
99	N-Oleoyl phenylalanine	13.87	428.3215	ESI-	C27H43NO3	91/147/164/280
100	Ribonic acid	0.80	165.0450	ESI-	C5H10O6	121/129/147
101	Palmitoylglycine	12.48	312.2589	ESI-	C18H35NO3	74/268
102	Galactonic acid	0.79	195.0555	ESI-	C6H12O7	159/177
103	Galacturonic acid	0.80	193.0399	ESI-	C6H10O7	59/71/85/175
104	Glutaminylserine	0.81	232.0984	ESI-	C8H15N3O5	132/189
105	Glutamylserine	0.85	233.0824	ESI-	C8H14N2O6	89/130/172/189/217
106	p-Methylhippuric acid	5.06	192.0711	ESI-	C10H11NO3	91/134/148
107	LPE14:1	8.76	422.2358	ESI-	C19H38NO7P	124/225
108	Acetylglycine	1.01	116.0398	ESI-	C4H7NO3	72/74
109	Indole-3-acetylglycine	5.32	231.0820	ESI-	C12H12N2O3	102/116/130/158/187
110	Alanine lactate	0.92	176.0610	ESI-	C6H11NO5	73/88/104/158
111	Threonylserine	0.82	205.0875	ESI-	C7H14N2O5	74/117/132/161/187
112	Threonylvaline	1.05	217.1239	ESI-	C9H18N2O4	100/116/144/173
113	Dihydroxybutyric acid	0.97	119.0395	ESI-	C4H8O4	83/101
114	Glucose	0.88	179.0606	ESI-	C6H12O6	59/71/89/119
115	N-Lactoyl-tryptophan	4.23	275.1082	ESI-	C14H16N2O4	160/188/231
116	ω-Muricholic acid	7.64	407.2848	ESI-	C24H40O5	389/371
117	β-Muricholic acid	7.97	407.2848	ESI-	C24H40O5	389/371
118	Cholic acid	8.58	407.2848	ESI-	C24H40O5	389/343/325/289/251
119	Ursodeoxycholic acid	8.75	391.2899	ESI-	C24H40O4	373/355
120	Deoxycholic acid	10.15	391.2899	ESI-	C24H40O4	373/355/327
121	Tauro-b-muricholic acid	6.31	514.2889	ESI-	C26H45NO7S	124/80
122	Tauroursodeoxycholic acid	7.05	498.2940	ESI-	C26H45NO6S	124/80
123	Taurodeoxycholic acid	8.23	498.2940	ESI-	C26H45NO6S	124/80
124	Isolithocholic acid	11.60	375.2950	ESI-	C24H40O3	357
125	Dehydrolithocholic acid	11.48	373.2793	ESI-	C24H38O3	355
126	Taurochenodeoxycholic acid	7.95	498.2940	ESI-	C26H45NO6S	124/80

Table S4. Sunitinib influenced 96 mouse serum metabolites using non-target metabolomics analysis.

No.	Compound	RT (min)	Mass M+H/M-H	Mode	Formula	MSMS
1	N6-Methyladenosine	1.01	282.1197	ESI+	C11H15N5O4	150
2	Indole-3-carboxaldehyde	4.78	146.0600	ESI+	C9H7NO	118
3	Glutamylarginine	0.82	304.1615	ESI+	C11H21N5O5	158/116/70
4	Pyroglutamylglycine	0.96	187.0713	ESI+	C7H10N2O4	141/112/102/84
5	5'-Methylthioadenosine	3.49	298.0968	ESI+	C11H15N5O3S	136/97
6	Indole-3-acetylglycine	5.10	233.0921	ESI+	C12H12N2O3	187/173/159/130/117
7	L-Cystine	0.78	241.0311	ESI+	C6H12N2O4S2	195/152/122/120
8	2-Methylcitric acid	0.72	207.0499	ESI+	C7H10O7	161/147
9	5,6-Dihydrouridine	0.88	247.0925	ESI+	C9H14N2O6	132/116/114
10	Caffeic acid 3-O-sulfate	0.64	261.0063	ESI+	C9H8O7S	245/217
11	N-Methyl-L-proline	0.67	130.0863	ESI+	C6H11NO2	84
12	Cytosine	1.01	112.0505	ESI+	C4H5N3O	95/69/67
13	Diaminopimelic acid	0.85	191.1026	ESI+	C7H14N2O4	173/145
14	LPC18:4	9.89	516.3084	ESI+	C26H46NO7P	184/104
15	Octadecanedioic acid	9.54	315.2530	ESI+	C18H34O4	279
16	alpha-Linolenic acid	9.54	279.2318	ESI+	C18H30O2	109/95/81/67
17	Creatine	0.84	132.0767	ESI+	C4H9N3O2	90/87
18	Trimethylamine N-oxide	0.81	76.0757	ESI+	C3H9NO	60/58
19	Alanylserine	0.94	177.0870	ESI+	C6H12N2O4	132/102/88
20	LPE18:1	10.71	480.3084	ESI+	C23H46NO7P	462/339
21	5-Methylcytosine	1.01	126.0662	ESI+	C5H7N3O	109/83/56
22	Citric acid	0.71	193.0343	ESI+	C6H8O7	149/105
23	Glutamylalanine	1.00	219.0975	ESI+	C8H14N2O5	145/130/116
24	Sphinganine 1-phosphate	9.62	382.2717	ESI+	C18H40NO5P	364/284/266
25	Sphinganine	9.43	302.3053	ESI+	C18H39NO2	266/112/108
26	Glutamylmethionine	1.02	279.1009	ESI+	C10H18N2O5S	133/130/102
27	Indole-3-carbinol	5.08	148.0757	ESI+	C9H9NO	120/92
28	Guanine	1.01	152.0567	ESI+	C5H5N5O	135/110
29	Urocanic acid	1.01	139.0502	ESI+	C6H6N2O2	121/95/93
30	Glutamylserine	0.87	235.0925	ESI+	C8H14N2O6	189/132/130/104/102
31	Indoleacrylic acid	5.35	188.0706	ESI+	C11H9NO2	170/142/115
32	7-Ketocholesterol	9.59	401.3414	ESI+	C27H44O2	175/95/81
33	Valerylcarnitine	4.07	246.1700	ESI+	C12H23NO4	187/85
34	Adenine	15.82	136.0618	ESI+	C5H5N5	119/108/94/92
35	LPC20:5	9.52	542.3241	ESI+	C28H48NO7P	258/184/104
36	Glutathione	0.94	308.0911	ESI+	C10H17N3O6S	162/130/116/84/76
37	Alanine	0.84	90.0550	ESI+	C3H7NO2	73/71/62
38	Arginine	0.82	175.1189	ESI+	C6H14N4O2	157/116/70/60
39	Asparagine	0.84	133.0608	ESI+	C4H8N2O3	87/74/59
40	Aspartic acid	0.99	134.0448	ESI+	C4H7NO4	116/88/74
41	Glutamate	0.84	148.0605	ESI+	C5H9NO4	130/102/84
42	Glycine	0.86	76.03930	ESI+	C2H5NO2	58
43	Histidine	0.80	156.0768	ESI+	C6H9N3O2	110/95/83
44	Lysine	0.76	147.1128	ESI+	C6H14N2O2	130/84
45	Serine	0.84	106.0499	ESI+	C3H7NO3	70/60
46	Threonine	0.84	120.0655	ESI+	C4H9NO3	102/84/74/56
47	Tyrosine	0.97	182.0812	ESI+	C9H11NO3	164/136
48	Valine	0.94	118.0863	ESI+	C5H11NO2	72/55
49	LPE16:0	10.75	454.2928	ESI+	C21H44NO7P	436/313
50	LPE18:0	12.15	482.3241	ESI+	C23H48NO7P	464/341
51	LPC15:0	10.18	482.3241	ESI+	C23H48NO7P	258/184/104
52	LPC16:0	10.66	496.3398	ESI+	C24H50NO7P	258/184/104
53	LPC16:1	10.05	494.3241	ESI+	C24H48NO7P	258/184/104
54	LPC17:0	11.63	510.3554	ESI+	C25H52NO7P	258/184/104
55	LPC18:1	11.26	522.3554	ESI+	C26H52NO7P	258/184/104
56	LPC19:0	12.77	538.3867	ESI+	C27H56NO7P	258/184/104
57	LPC20:0	13.67	552.4024	ESI+	C28H58NO7P	184/104
58	LPC20:1	12.72	550.3867	ESI+	C28H56NO7P	258/184/104
59	LPC20:2	11.65	548.3711	ESI+	C28H54NO7P	258/184/104
60	LPC20:3	10.71	546.3554	ESI+	C28H52NO7P	258/184/104
61	LPC22:1	10.50	578.4180	ESI+	C30H60NO7P	184/104
62	Allantoin	0.83	157.0367	ESI-	C4H6N4O3	114/99
63	N-Acetyltryptophan	5.45	245.0932	ESI-	C13H14N2O3	203/159/116/98/74/58
64	Adenosine 3',5'-diphosphate	0.88	426.0221	ESI-	C10H15N5O10P2	408/328/272/158/134/79
65	5-Sulfosalicylic acid	3.60	216.9812	ESI-	C7H6O6S	199/171
66	3-Sulfinoalanine	0.84	152.0023	ESI-	C3H7NO4S	107/88
67	N-Nonanoylglycine	6.12	214.1449	ESI-	C11H21NO3	170/59
68	N-Formyl-L-methionine	3.62	176.0387	ESI-	C6H11NO3S	128/98
69	Cysteine-S-sulfate	0.81	199.9693	ESI-	C3H7NO5S2	136/81/74
70	Dodecanoylcarnitine	11.96	342.2650	ESI-	C19H37NO4	199/62
71	Valylserine	0.88	203.1037	ESI-	C8H16N2O4	159/115/132
72	N-Acetylglutamine	0.88	187.0724	ESI-	C7H12N2O4	143/132
73	Glutamylthreonine	0.88	247.0936	ESI-	C9H16N2O6	203/159/145
74	Guanosine 2'-monophosphate	1.01	362.0507	ESI-	C10H14N5O8P	211/97/79
75	L-Tryptophan	3.68	203.0826	ESI-	C11H12N2O2	159/142/116/74
76	Undecanoylglycine	7.33	242.1762	ESI-	C13H25NO3	198/74/59
77	Pyroglutamic acid	1.02	128.0353	ESI-	C5H7NO3	84/82
78	Gluconic acid	0.79	195.0510	ESI-	C6H12O7	160/129/99/75
79	Glucuronic acid	0.78	193.0354	ESI-	C6H10O7	157/131/113/85/72/59

80	5-Hydroxyindoleacetic acid	4.78	190.0510	ESI-	C10H9NO3	157/146/144
81	Benzoic acid	4.81	121.0295	ESI-	C7H6O2	92/77
82	Indoxyl sulfate	4.79	212.0023	ESI-	C8H7NO4S	132/80
83	Dihydroferulic acid	5.53	195.0663	ESI-	C10H12O4	151
84	Azelaic acid	5.82	187.0976	ESI-	C9H16O4	169/124/123/97/57
85	Pimelic acid	4.38	159.0663	ESI-	C7H12O4	140/115/97/95
86	Sebacic acid	6.50	201.1132	ESI-	C10H18O4	183/139/111/57
87	ω-Muricholic acid	7.64	407.2803	ESI-	C24H40O5	389/371
88	β-Muricholic acid	7.97	407.2803	ESI-	C24H40O5	389/371
89	Cholic acid	8.58	407.2803	ESI-	C24H40O5	389/343/325/289/251
90	Ursodeoxycholic acid	8.75	391.2854	ESI-	C24H40O4	373/355
91	Tauro-β-muricholic acid	6.31	514.2843	ESI-	C26H45NO7S	124/80
92	Taurocholic acid	7.09	514.2843	ESI-	C26H45NO7S	124/80
93	Tauroursodeoxycholic acid	7.05	498.2895	ESI-	C26H45NO6S	124/80
94	Taurochenodesoxycholic acid	7.95	498.2895	ESI-	C26H45NO6S	124/80
95	Chenodeoxycholic acid	9.95	391.2854	ESI-	C24H40O4	373/354
96	Taurodeoxycholic acid	8.23	498.2895	ESI-	C26H45NO6S	124/80

Table S5. Primer sequences for QPCR.

Primers	Sequence	Sequence
ZO-1	CCATCTTTGGACCGATTGCTG	TAATGCCCGAGCTCCGATG
Occludin	TTGGGAGCCTTGACATCTTGTTC	GCCATACATGTCATTGCTTGGTG
Sqstm1	GAACTCGCTATAAGTGCAGTGT	AGAGAAGCTATCAGAGAGGTGG
Ctsd	GCTTCCGGTCTTTGACAACCT	CACCAAGCATTAGTTCTCCTCC
MapLC3b	TTATAGAGCGATACAAGGGGGAG	CGCCGTCTGATTATCTTGATGAG
Lamp1	TGCTCCGGGATGCCACTAT	TGTTGTCCTTTTTCAGGTAGGTG
Caspase3	AACGGACCTGTGGACCTGAA	TCAATACCGCAGTCCAGCTC
Bcl2	CACGGTGGTGGAGGAACTCT	GATGCCGGTTCAGGTACTCA
Bax	AGGACGCATCCACCAAGAAG	GCCACACGGAAGAAGACCTC
Mt-nd6	CACAACTATATATTGCCGCTACC	GCTACTGAGGAATATCCAGAGAC
Sdha	TGCTGGAGAAGAATCGGTTATG	ACAGCATCAGATTCTGCAGCTCC
Sdhb	AATTTGCCATTTACCGATGGGA	AGCATCCAACACCATAGGTCC
Uqcrc1	AGACCCAGGTCAGCATCTTG	GCCGATTCTTTGTTCCCTTGA
Mt-co1	GTCTGATCCGTACTTATTACAG	GCTCATACTATTCCTATATAGCCG
Atp5a	GAGACTGGGCGTGTGTTAAGCA	CATTACCGAGGGCGTCAACCAC
Nrf1	CAGACGACGCAAGCATCAGAG	GCTCCGACGGCTGCTGCGGTTTC
Tfam	ATTCCGAAGTGTTTTTCCAGCA	TCTGAAAGTTTTGCATCTGGGT
Il1b	CCCTGCAGCTGGAGAGTGTGGA	TGTGCTCTGCTTGTGAGGTGCTG
Il6	CGGAGAGGAGACTTCACAGAGGA	TTTCCACGATTTCCCAGAGAACA
Tnfa	CCACCACGCTCTTCTGTCTAC	AGGGTCTGGGCCATAGAACT
Vcam	CCGGCATATACGAGTGTGAA	GGAGTTCGGGCGAAAAATAG
Ccl2	AGGTCCCTGTCATGCTTCTG	GGGATCATCTTGCTGGTGAA
F4/80	TGCATCTAGCAATGGACAGC	GCCTTCTGGATCCATTTGAA
Caspase11	ACAAACACCCTGACAAACCAC	CACTGCGTTCAGCATTGTTAAA
Caspase1	ATGCCGTGGAGAGAAACAAG	CCAGGACACATTATCTGGTG
Nlrp3	GCTCCAACCATTCTCTGACC	AAGTAAGGCCGGAATTCACC
Gpx4	ACGATGCCCACCCACT	CCACGCAGCCGTTCTT
Cox2	TGACCCCCAAGGCTCAAATAT	TGAACCCAGGTCCTCGCTTA
Acta2	GTCCCAGACATCAGGGAGTAA	TCGGATACTTCAGCGTCAGGA
Col1a1	CATGTTCAGCTTTGTGGACCT	GCAGCTGACTTCAGGGATGT
Tgfb	GGAGAGCCCTGGATACCAAC	CAACCCAGGTCCTTCCTAAA
Cd31	ACTTCTGAACTCCAACAGCGA	CCATGTTCTGGGGGTCTTTAT
Lyve1	GCTCTCCTCTTCTTTGGTGCT	TGACGTCATCAGCCTTCTCTT
Icam	AACAGTTCACCTGCACGGAC	GTCACCGTTGTGATCCCTG
Pai1	CTCCACAGCCTTTGTCATCTC	GATTGTCTCTGTCGGGTTGTG
Tbxa2r	TTGGTGCTTCCTGACACTCG	CCACACTAACGGTGTTGAGC
Tbxas1	TACCATAGTGACTGTGACTCTGC	GGTGCCTGATGCCCAACTT
Coagulation factor II	CCGAAAGGGCAACCTAGAGC	GGCCCAGAACACGTCTGTG
Coagulation factor III	CTGGAAAAACAAGTGCTTCTCG	ACAGAGAGGACCTTTGCTTCA
Coagulation factor IX	ATGCTGGTGCCAAGTTGGATT	CTCAGTGCAGGAACAAATTACCT
Coagulation factor V	GAAAAGCCACGATCTAGCAACT	TGAGGATGGATGCTTAGTGGT
Coagulation factor VII	AAAGGCGTGCCAACTCACTC	CTCCGTTCTGACATGGATTCG
Coagulation factor VIII	TGACCCTCCATGTCTCACTTAC	GTGCATTTTAGGCCAGTCTCT
Coagulation factor X	AGGACTCGGAGGGCAAACT	TCACGGACCTCTTCATAAGAACA
CK19	CTCGGATTGAGGAGCTGAAC	TCACGCTCTGGATCTGTGAC
Fxr	TGGGCTCCGAATCCTCTTAGA	TGGTCCTCAAATAAGATCCTTGG
Shp	TCTGCAGGTCGTCCGACTATTC	AGGCAGTGGCTGTGAGATGC
Cyp7a1	GGGAATGCCATTTACTTGGA	GTCCGGATATTCAAGGATGC
Cyp8b1	TCCTCAGGGTGGTACAGGAG	GATAGGGGAAGAGAGCCACC
Ntcp	AGGGGGACATGAACCTCAG	TCCGTCGTAGATTCCTTTGC
Oatp1	ACTCCCATAATGCCCTTGG	TAATCGGGCCAACAATCTTC
Oatp4	ACCAAACTCAGCATCCAAGC	TAGCTGAATGAGAGGGCTGC
Ostb	GTATTTTCGTGCAGAAGATGCG	TTTCTGTTTGCCAGGATGCTC
Mrp3	CTGGGTCCCCTGCATCTAC	GCCGTCTTGAGCCTGGATAAC
Mrp4	AGCTTCAACGGTACTGGGATA	TCGTCGGGGTCATACTTCTC
Bsep	CCAGAACATGACAAACGGAA	AAGGACAGCCACACCAACTC
Mrp2	TCCAGGACCAAGAGATTTGC	TCTGTGAGTGCAAGAGACAGGT
Fgf15	ATGGCGAGAAAGTGGAACGG	CTGACACAGACTGGGATTGCT
Ibabp	CCCCAACTATCACCAGACTTC	ACATCCCCGATGGTGGAGAT
Osta	CACTGGCTCAGTTGCCATTT	GCATACGGCATAAAACGAGGT

Cyp27a1	GGAAGGTGCCCCAGAACAA	GCGCAGGGTCTCCTTAATCA
Cyp7b1	GGAGCCACGACCCTAGATG	GCCATGCCAAGATAAGGAAGC
Clock	ATGGTGTTTACCGTAAGCTGTAG	CTCGCGTTACCAGGAAGCAT
Bmal1	CGCCGCTCTCTGTTCTGTAG	GTGTCGAGAAACGTACTCCATAG
Dbp	CTCGCGTTACCAGGAAGCAT	CCAGGTCCACGTATTCCACG
18S	ATTACCGCGGCTGCTGGC	CGGCTACCACATCCAAGGAA
Eubacteria	ACTCCTACGGGAGGCAGCAG	ATTACCGCGGCTGCTGG
Bacteroides	GAGAGGAAGGTCCCCCAC	CGCTACTTGGCTGGTTCAG
Firmicutes	GGAGYATGTGGTTTAATTCGAAGCA	AGCTGACGACAACCATGCAC
Proteobacteria	TCGTCAGCTCGTGTYGTGA	CGTAAGGGCCATGATG
B. bifidum	AAGACACCCCCGAAAGGCGT	TCAAGGCGGAGTCGCTAGTAA
P. distasonis	GGACACGTCCCGCACTTTAT	TTCTGAGAGGAAGGTCCCCC
Helicobacter pullorum	GTCTTTTGAGTGGATTGGCT	CACTCCGGGTGCTTGAT
Aeromonas sp	GAAGGCCAAGTCGGCCGCCAG	ATCTTGGCATCGCCCGGGTTTTC
Campylobacter jejuni	CTTGCGGTCATGCTGGACATAC	AGCACCACCCAAACCCTCTTCA
Streptococcus uberis	CGAAATTGGAACAGGTAAAGG	CTGACGATAAGTTGAATAGATGAC
Human-Sqstm1	GACTACGACTTGTGTAGCGTC	AGTGTCCGTGTTTCACCTTCC
Human-Icam	ATGCCCAGACATCTGTGTCC	GGGGTCTCTATGCCCAACAA
Human-Cd31	AACAGTGTTGACATGAAGAGCC	TGTAAAACAGCACGTCATCCTT
Human-Gapdh	GGAGCGAGATCCCTCCAAAAT	GGCTGTTGTCATACTTCTCATGG
